# Unexpected Noncovalent Off-Target Activity of Clinical BTK Inhibitors Leads to Discovery of a Dual NUDT5/14 Antagonist

**DOI:** 10.1021/acs.jmedchem.4c00072

**Published:** 2024-04-18

**Authors:** Esra Balıkçı, Anne-Sophie M. C. Marques, Ludwig G. Bauer, Raina Seupel, James Bennett, Brigitt Raux, Karly Buchan, Klemensas Simelis, Usha Singh, Catherine Rogers, Jennifer Ward, Carol Cheng, Tamas Szommer, Kira Schützenhofer, Jonathan M. Elkins, David L. Sloman, Ivan Ahel, Oleg Fedorov, Paul E. Brennan, Kilian V. M. Huber

**Affiliations:** † Centre for Medicines Discovery, Nuffield Department of Medicine, University of Oxford, Old Road Campus, Roosevelt Drive, Oxford OX3 7FZ, U.K.; ‡ Target Discovery Institute, Nuffield Department of Medicine, University of Oxford, Old Road Campus, Roosevelt Drive, Oxford OX3 7FZ, U.K.; § Alzheimer’s Research UK Oxford Drug Discovery Institute, Nuffield Department of Medicine, University of Oxford, Old Road Campus, Roosevelt Drive, Oxford OX3 7FZ, U.K.; ∥ Sir William Dunn School of Pathology, University of Oxford, South Parks Road, Oxford OX1 3RE, U.K.; ⊥ Departments of Discovery Chemistry, Merck & Co. Inc., 33 Avenue Louis Pasteur, Boston, Massachusetts 02115, United States

## Abstract

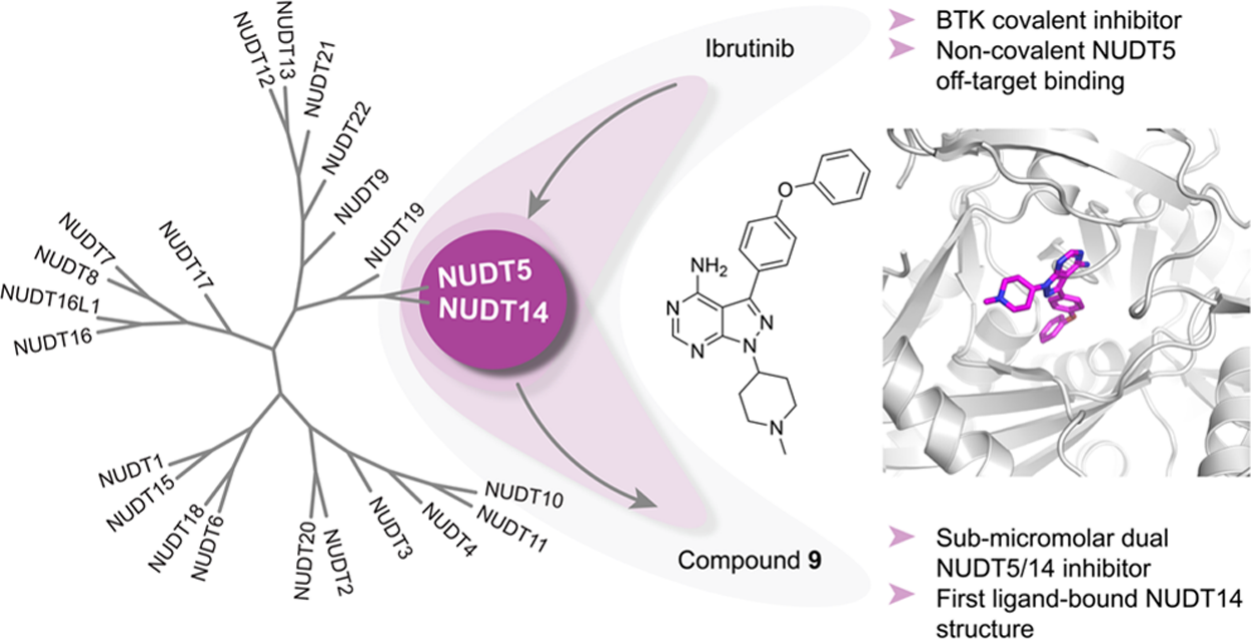

Cofactor mimicry represents an attractive strategy for the development of enzyme inhibitors but can lead to off-target effects due to the evolutionary conservation of binding sites across the proteome. Here, we uncover the ADP-ribose (ADPr) hydrolase NUDT5 as an unexpected, noncovalent, off-target of clinical BTK inhibitors. Using a combination of biochemical, biophysical, and intact cell NanoBRET assays as well as X-ray crystallography, we confirm catalytic inhibition and cellular target engagement of NUDT5 and reveal an unusual binding mode that is independent of the reactive acrylamide warhead. Further investigation of the prototypical BTK inhibitor ibrutinib also revealed potent inhibition of the largely unstudied NUDIX hydrolase family member NUDT14. By exploring structure–activity relationships (SARs) around the core scaffold, we identify a potent, noncovalent, and cell-active dual NUDT5/14 inhibitor. Cocrystallization experiments yielded new insights into the NUDT14 hydrolase active site architecture and inhibitor binding, thus providing a basis for future chemical probe design.

## Introduction

NUDIX hydrolases are conserved throughout eukaryotes, bacteria, archaea, and viruses. Their name derives from a common substrate structure, **nu**cleoside **di**phosphate linked to another moiety **X**.^1^ The characteristic NUDIX box is shared between family members and contains a sequence motif “GX_5_EX_5_ [UA] XREX_2_EEXGU”, where U is usually valine, leucine, or isoleucine and X is any amino acid. NUDIX enzymes recognize a wide range of substrates including canonical and oxidized forms of (d)NTPs, dinucleoside polyphosphates (NpnN), nucleotide sugars, alcohols, and capped RNAs.^2^ To date, more than 20 family members have been identified in mammals but their distinct biological functions and cellular roles remain largely unexplored.^3^ In particular, the nature of their physiological substrates remains an object for debate, e.g., NUDT5 not only predominantly hydrolyzes ADP-ribose (ADPr) to AMP and ribose-5′-phosphate but also exhibits activity against 8-oxo-dGDP, 8-oxo-GDP, 8-oxo-dADP, 2-oxo-dADP, and 5-CHO-dUDP.^4,5^ Attachment of ADPr to proteins serves as a trigger signal for essential biological processes such as DNA repair, gene transcription, protein degradation, and cell death.^6,7^ In cells, free ADPr levels are controlled by NUDT5 and NUDT9 to maintain NAD^+^ pools after DNA damage^8,9^ and prevent deleterious nonenzymatic ADP-ribosylation of proteins.^10^ Notably, NUDT5 has recently been suggested as a key enzyme for nuclear ATP synthesis required for progestin-mediated chromatin remodeling, transcription, and tumor cell proliferation.^11^ Suppression of NUDT5 activity by potent inhibitors was found to impair breast cancer cell growth in various model systems in line with reports suggesting NUDT5 overexpression as a potential prognostic marker for this tumor type.^12,13^ A recent systematic exploration of the NUDIX family confirmed that both NUDT5 and NUDT14 are able to hydrolyze ADPr and ADP-glucose,^3^ yet the function and cellular roles of NUDT14 remain elusive. Given the importance of ADPr for cellular signaling, there is a strong need for new tool compounds to facilitate further investigation of NUDIX proteins in these and other yet-to-be-discovered pathways. Here, by screening a small kinase inhibitor library, we identify ibrutinib (**1**) as a dual inhibitor of NUDT5 and NUDT14 catalytic activities. By investigating structure–activity relationship (SAR) around **1**, we discover a potent dual cell-active NUDT5/NUDT14 inhibitor, which, together with novel cocrystal structures of NUDT5 and NUDT14, should pave the way for future chemical probe development.

## Results and Discussion

**Figure 1:**
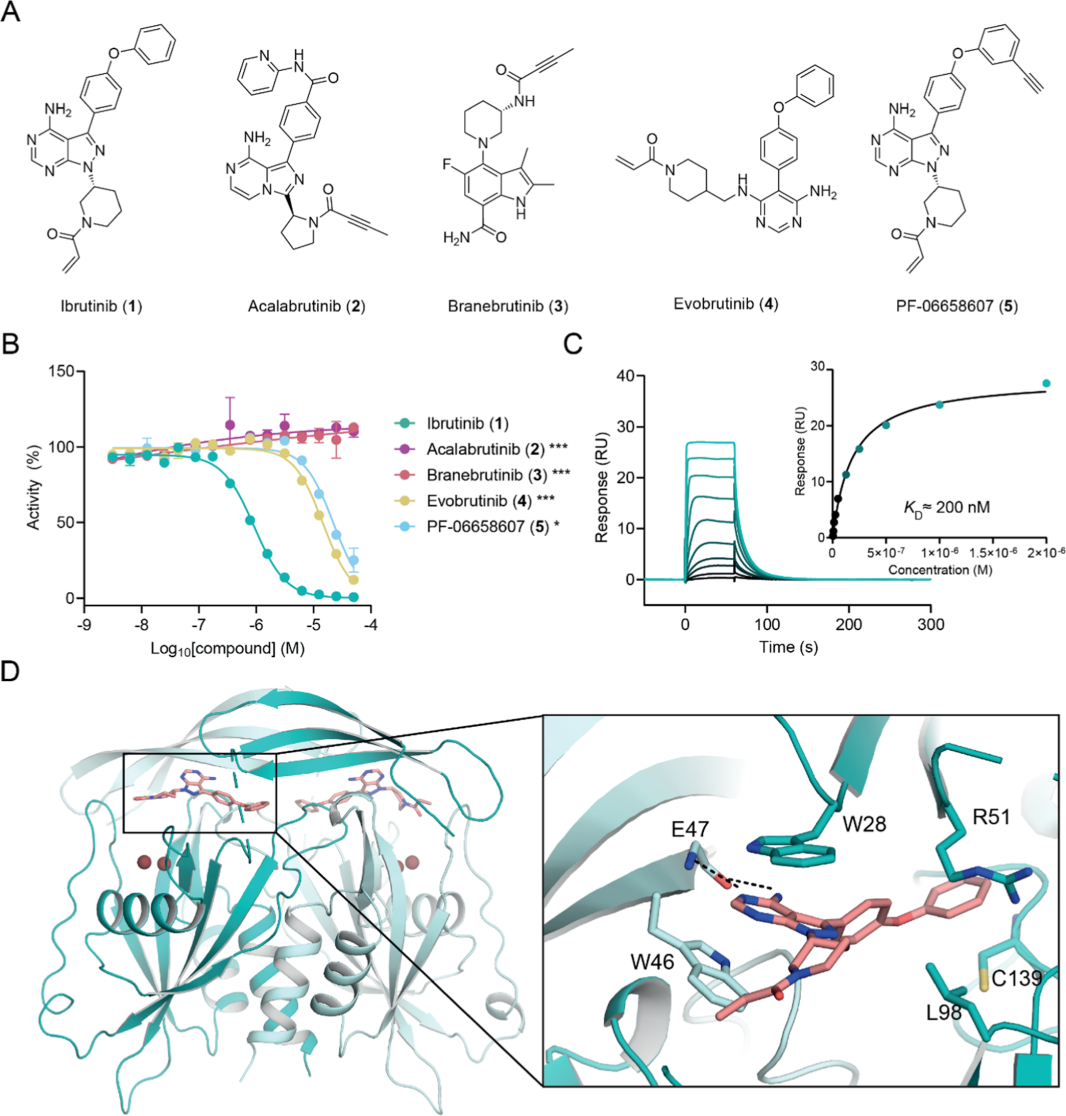
Identification and validation of ibrutinib (**1**) as a noncovalent NUDT5 inhibitor. (A) Chemical structures of BTK inhibitors. (B) NUDT5 catalytic activity assay results (**1**: IC_50_ = 0.837 ± 0.329 μM, **4**: IC_50_ = 13.9 ± 0.6 μM, **5**: IC_50_ = 21.2 ± 1.0 μM). Data are shown as mean ± standard deviation (SD) and are based on three technical replicates. Graph is representative of two independent biological replicates (*n* = 2). Stars indicate Student’s *t* test *p*-value comparing compound activity at the highest concentration against **1** (* < 0.05, *** < 0.001). (C) SPR sensorgram showing binding of **1** to NUDT5 (*K*_D_ ≈ 200 nM). (D) Crystal structure of ibrutinib (**1**) bound to NUDT5 (PDB: 8RDZ). Compound **1** occupies the active site of the NUDT5 dimer where it mediates π–π stacking interactions with W46 of chain A (teal) and W28 of chain B (pale teal). An additional hydrophobic interaction with R51 in chain B and a hydrogen bond with the main chain of E47 in chain A can be observed. Compound **1** (salmon) and interacting residues are shown in stick representation.

Building on previous efforts that identified highly potent and selective NUDT1 (MTH1) inhibitors from kinase inhibitor screening,^14−16^ we hypothesized that this approach could be exploited further for the discovery of novel tool compounds for other NUDIX proteins. Structures of human NUDT5 bound to ADPr and AMP indicate the importance of the adenine moiety for substrate binding, with strong π–π stacking interactions observed in the enzymatic pocket.^17^ We screened a small set of kinase inhibitors in an AMP-Glo assay to monitor the NUDT5-mediated conversion of ADPr into AMP and ribose-5-phosphate. Surprisingly, we identified ibrutinib (**1**), an irreversible covalent BTK inhibitor, as a hit. Ibrutinib (**1**) is an FDA-approved Bruton’s tyrosine kinase (BTK) antagonist used for the treatment of various cancers including B-cell leukemias and lymphomas.^18^ To rationalize this observation and investigate SAR, we extended our efforts toward an expanded selection of four commercially available BTK inhibitors including acalabrutinib (**2**), branebrutinib (**3**), evobrutinib (**4**), and PF-06658607 (**5**) (Figure 1A,B). Among the compounds tested, **1** stood out as the most potent inhibitor (IC_50_ = 0.837 ± 0.329 μM), yet the other two acrylamide-based molecules **4** and **5** also exhibited weak activity (IC_50_ > 10 μM). Compounds **2** and **3**, both containing ynamide moieties, did not show any inhibition at concentrations of up to 50 μM. We next confirmed direct binding of **1** to NUDT5 by surface plasmon resonance (SPR) and determined a *K*_D_ value of approximately 200 nM (Figure 1C). To understand the binding mode of **1**, we solved a cocrystal structure with NUDT5 at a 2 Å resolution (Figure 1D). This suggested that no covalent bond was formed between the acrylamide warhead and the protein despite the presence of a potentially accessible proximal cysteine (C139) due to the acrylamide moiety pointing out of the protein toward the solvent. Similar to previously determined NUDT5 inhibitor-bound structures,^12^ the main heterocyclic ring of **1** is stabilized by π–π stacking interactions between W46 of chain A and W28 of chain B in the active site (Figure 1D). In addition, the aminopyrimidine moiety is stabilized by hydrogen bonds with E47 and the phenoxy group protrudes deep into the hydrophobic pocket where interactions are mediated with the side chain of R51.

**Figure 2:**
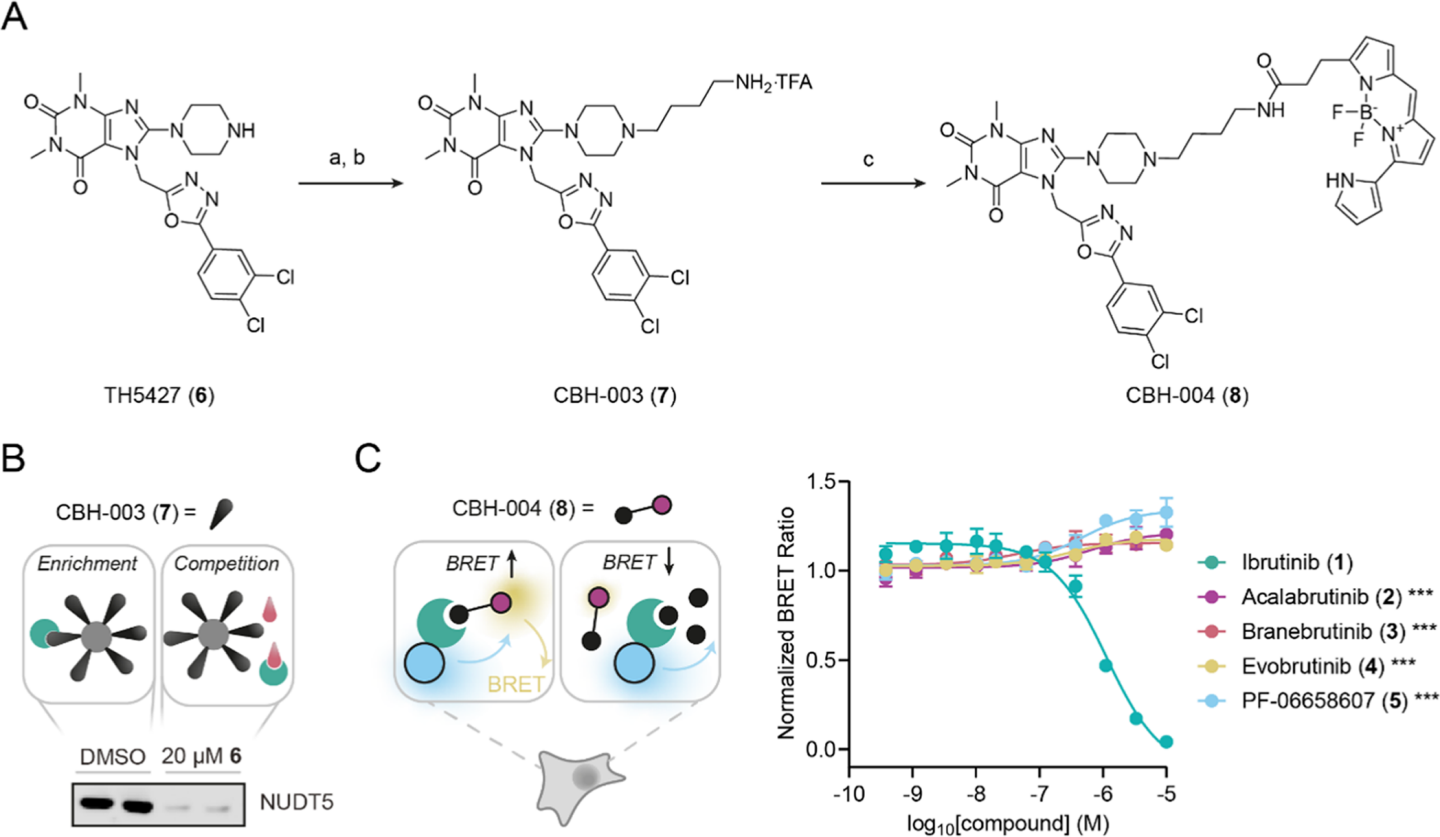
Live-cell NanoBRET target engagement (TE) assay for NUDT5. (A) Synthesis of affinity probe CBH-003 (**7**) and energy-transfer probe (ETF) CBH-004 (**8**). Reagents and conditions: (a) 4-(Boc-amino) butyl bromide, DIPEA, dimethyl sulfoxide (DMSO), 80 °C, 2 h, 31%; (b) TFA, dichloromethane (DCM), rt, overnight, quantitative (c) succinimidyl ester, DIPEA, *N*,*N*-dimethylformamide (DMF), rt, 4 h, 15%. (B) Western blot using specific NUDT5 antibody confirms enrichment by CBH-003 (**7**) affinity matrix and competition of NUDT5 by **6** (20 μM). (C) NUDT5 NanoBRET TE assay results for compounds **1**–**5** in HEK293 cells. Addition of NUDT5 inhibitor test compounds leads to displacement of the ETF resulting in reduced BRET. Data are shown as mean ± SD and are based on three technical replicates. Graph is representative of two independent biological replicates (*n* = 2). Stars indicate Student’s *t* test *p*-value comparing compound activity at the highest concentration against **1** (*** < 0.001).

We next asked if **1** is able to engage NUDT5 in cells and developed an NUDT5 live-cell target engagement (TE) assay based on the NanoBRET system (Figure 2).^19^ Taking advantage of the cocrystal structure published for TH5427 (**6**) bound to NUDT5, we designed and synthesized an affinity probe, CBH-003 (**7**), with a free primary amine appended at the solvent-exposed piperazine N4-position of **6** (Figure 2A). The functional probe **7** was obtained by reacting **6** with *N*-Boc butyl bromide in the presence of *N*,*N*-diisopropylethylamine (DIPEA) followed by Boc group removal using trifluoroacetic acid (TFA). We next confirmed that **7** retained binding to endogenous NUDT5 by performing a pull-down experiment followed by Western blotting using a specific NUDT5 antibody (Figure 2B). Subsequent chemoproteomics further corroborated the strong binding of **7** to NUDT5 (Figure S1). CBH-004 (**8**), which we envisioned as a NanoBRET-compatible energy-transfer probe (ETF) to assess target engagement, was prepared from **7** by amide coupling with the corresponding BODIPY succinimidyl ester under basic conditions (Figure 2A). Titration of **8** against N- and C-terminal NUDT5-NanoLuc fusions in the absence or presence of the parent inhibitor **6** in HEK293 cells suggested an optimal assay window at 2.5 nM in the case of the N-terminal NanoLuc-NUDT5 fusion (Figure S2). With this assay in hand, we confirmed in-cell NUDT5 target engagement by **1** (EC_50_ = 1.23 ± 0.10 μM), whereas the other BTK inhibitors did not exhibit any significant activity (EC_50_ > 10 μM) in line with the catalytic assay results (Figure 2C).

Within the NUDIX family, NUDT5 and NUDT14 are closely related, and consistent with this notion, previous reports have suggested that both enzymes are able to hydrolyze ADPr.^3^ Thus, we next expressed and purified recombinant human NUDT14 and established a catalytic assay to test if **1** exhibited any cross-reactivity. Interestingly, results indicated that ibrutinib (**1**) also potently suppresses the NUDT14 catalytic activity at submicromolar concentrations (IC_50_ = 0.990 ± 0.110 μM) (Table 1). We decided to perform an SAR study to investigate the potential of this scaffold for the development of new distinct selective chemical tools to study NUDT5 and NUDT14 biology. Our primary objectives were to improve potency and evaluate the possibility of developing covalent NUDT5 inhibitors targeting C139 as suggested by our cocrystal structure. Since the main heterocyclic core in **1** appeared essential for binding, we focused on modifying the ring at N1-position (compounds **9**–**11**) and placed an electrophilic acrylamide at the C3-position, which should position it within a reasonable distance of C139 in NUDT5 (compounds **12**–**15**).

**Table 1:**
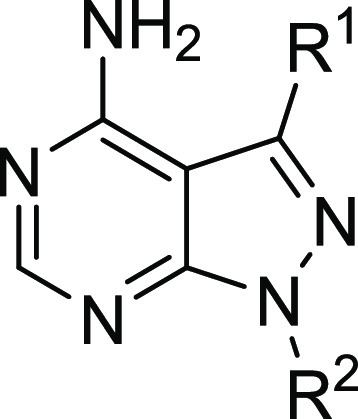

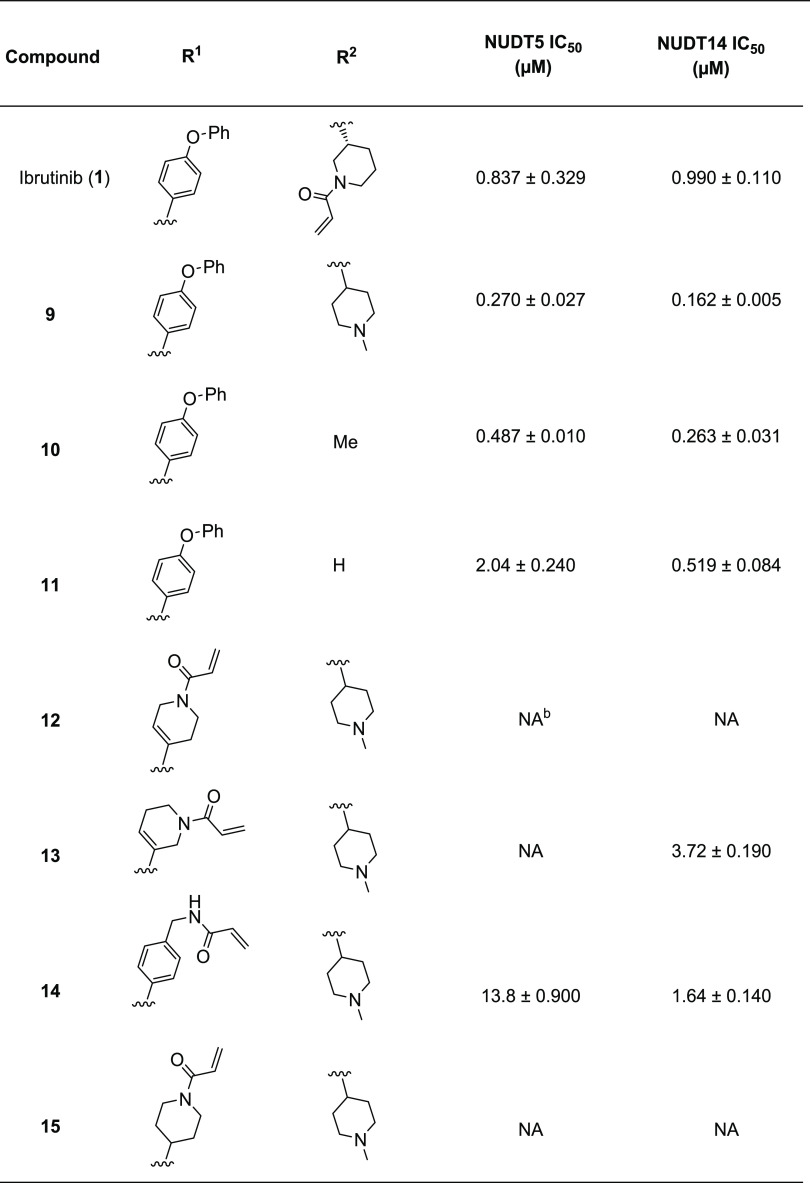
NUDT5 and NUDT14 Catalytic Assay Results^a^

a IC_50_ values and SD were calculated from two independent biological replicates (*n* = 2).

b NA, not active (IC_50_ > 50 μM).

**Scheme 1:**
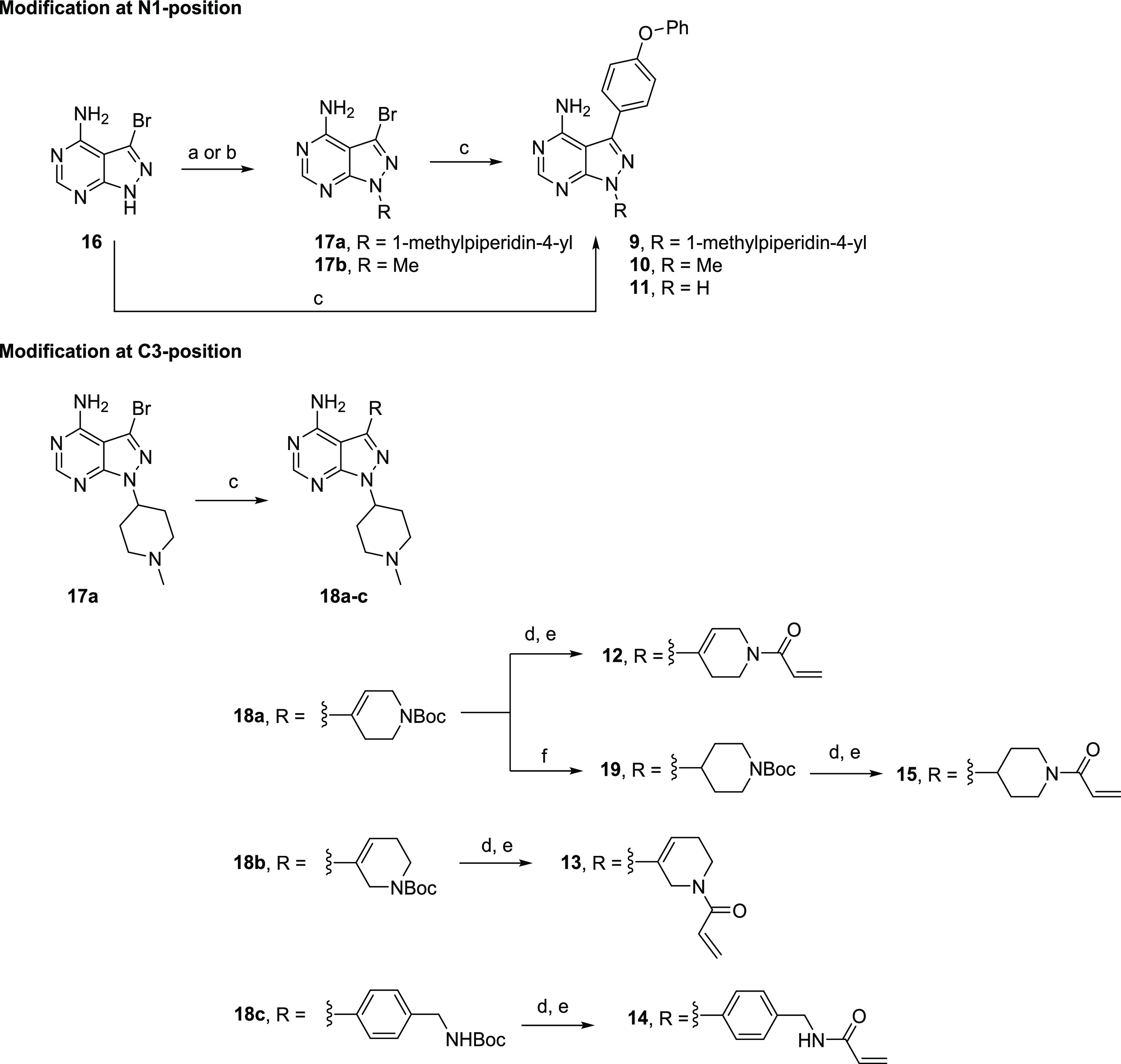
Synthesis of Ibrutinib Analogues Reagents and conditions: (a) 4-hydroxy-1-methylpiperidine, PS–PPh_3_, diisopropyl azodicarboxylate (DIAD), tetrahydrofuran (THF), rt, 12 h, 35%; (b) MeI, Cs_2_CO_3_, DMF, rt, 12 h, 23%; (c) R-B(OH)_2_ or R-BPin, Pd(dppf)Cl_2_·DCM, Na_2_CO_3_, 1,4-dioxane/H_2_O, 80 °C, overnight, 31–93%; (d) 4 N HCl in 1,4-dioxane/DCM, rt, 2 h; (e) acryloyl chloride, NEt_3_, DCM, rt, 30 min, 43–70% over two steps; and (f) H_2_, 10% Pd/C, MeOH, 45 °C, overnight, 93%.

Scheme 1 illustrates the synthetic procedure starting with the functionalization of the main building block 3-bromo-1*H*-pyrazolo[3,4-*d*]pyrimidin-4-amine (**16**). To replace the electrophilic acrylamide present in **1**, a methylpiperidin-4-yl moiety or a methyl group was added at the N1-position via Mitsunobu or S_N_2 reaction to afford intermediates **17a** and **17b**. A phenoxyphenyl moiety was added at the C3-position using the palladium-catalyzed Suzuki–Miyaura coupling, yielding compounds **9** and **10**. The free amine analogue **11** was accessed in the same manner in one step from **16**. To install the acrylamide warhead at the C3-position, intermediate **17a** was reacted with two different boronic esters via the Suzuki–Miyaura coupling to afford regioisomers **18a** and **18b**. Under the same reaction conditions, **17a** was treated with 4-[(*N*-Boc-amino)methyl]phenylboronic acid to yield **18c**. Subsequent HCl-mediated deprotection of the *N*-Boc group in **18a**–**c** followed by amide coupling with acryloyl chloride on the crude material afforded the acrylamide-containing products **12**–**14**. Compound **15**, a saturated analogue of **12**, was prepared by hydrogenation of **18a** to give intermediate **19** followed by Boc deprotection and amide coupling.

All compounds were tested against NUDT5 and NUDT14 in their respective catalytic assays (Table 1). The *N*-methylpiperidine analogue **9** lacking a reactive warhead exhibited significantly increased potency against both NUDT5 (IC_50_ = 0.270 ± 0.027 μM) and NUDT14 (IC_50_ = 0.162 ± 0.005 μM). This observation is in line with the cocrystal structure of **1** bound to NUDT5, suggesting that the warhead does not contribute to inhibitor binding (Figure 1D). Replacing the *N*-methylpiperidine by a methyl group (compound **10**) or hydrogen atom (compound **11**) led to reduced inhibitory activity for both proteins. Disappointingly, none of the compounds with electrophilic warheads exhibited any increased potency against NUDT5. This was further corroborated by intact protein MS analysis, which indicated that the compounds were not able to engage C139 and form covalent adducts with NUDT5 (Figure S3). Compounds **13** and **14** also exhibited NUDT14 activity in the micromolar range (IC_50_ = 3.72 ± 0.190 μM and 1.64 ± 0.140 μM, respectively) but **15** remained inactive. Similar to NUDT5, none of the compounds appeared to form any covalent adducts with NUDT14 (Figure S4).

**Figure 3:**
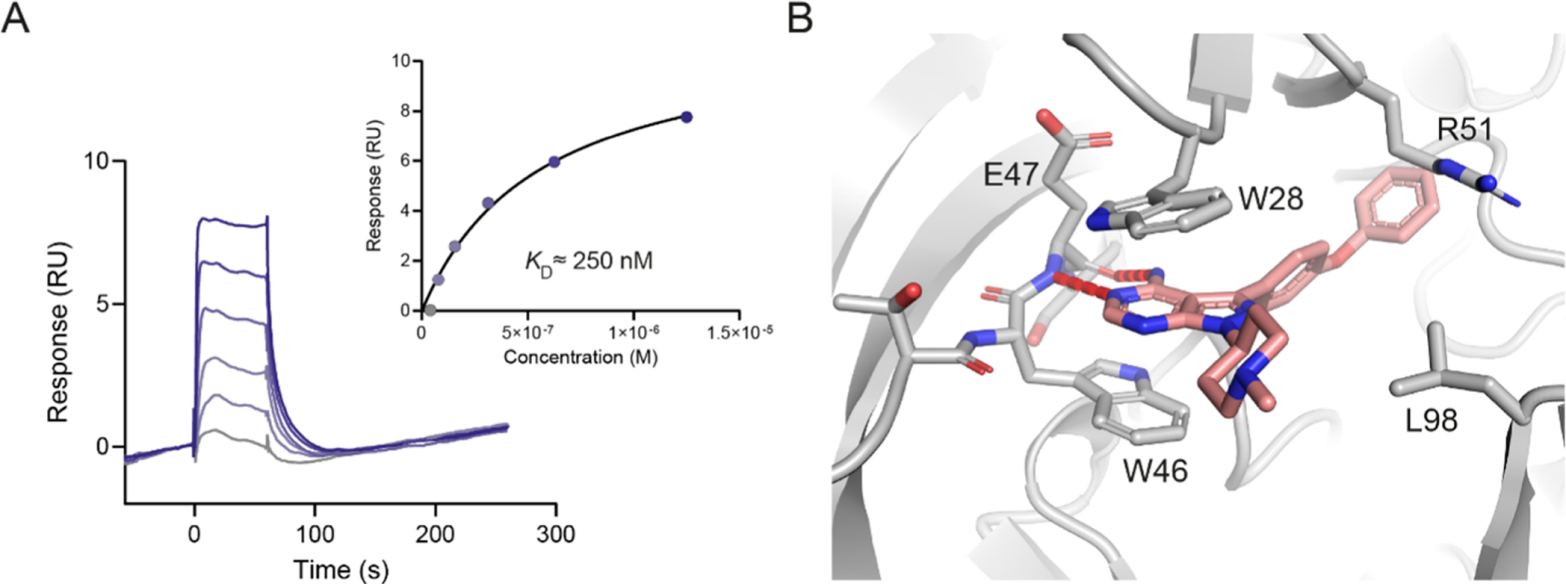
Biophysical assay results and cocrystal structure of compound **9** bound to NUDT5. (A) SPR results for compound **9** indicating a *K*_D_ value of approximately 250 nM for NUDT5. Data are from two independent experiments (*n* = 2). (B) Cocrystal structure of NUDT5 bound to compound **9** (PDB: 8RIY). Compound **9** occupies the active site of NUDT5 where it mediates π–π stacking interactions with W46 of chain A (dark gray) and W28 of chain B (light gray). An additional hydrophobic interaction with R51 in chain B and a hydrogen bond with the main chain of E47 in chain A can be observed. Compound **9** (salmon) and interacting residues are shown in stick representation.

Based on these results, **9** appeared as the most potent dual inhibitor of NUDT5 and NUDT14. We confirmed direct binding of **9** to NUDT5 by SPR (*K*_D_ ≈ 250 nM; Figure 3A) and solved a cocrystal structure of NUDT5 in complex with **9** at a 2.29 Å resolution, suggesting a similar binding mode as observed for compound **1** (Figure 3B–D).

**Figure 4:**
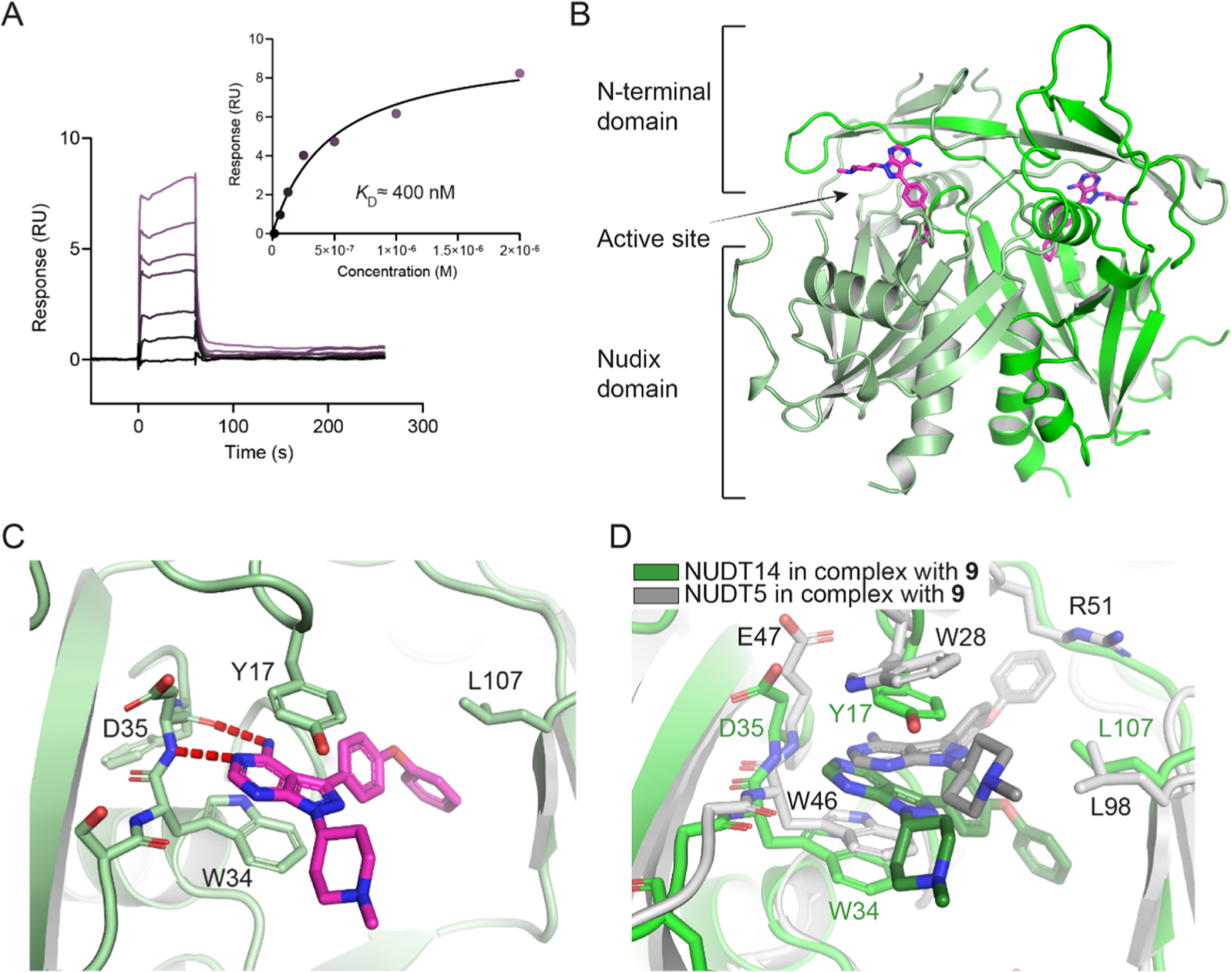
Biophysical assay results and cocrystal structure of compound **9** bound to NUDT14. (A) SPR results for compound **9** indicating a *K*_D_ value of approximately 400 nM for NUDT14. Data are from two independent experiments (*n* = 2). (B) Overall structure of NUDT14 depicting the N-terminal domain, the NUDIX domain, and the active site (PDB: 8OTV). Chain A (pale green) and chain B (green) constitute a dimer. Compound **9** (violet) is located in the catalytic site. (C) Compound **9** binds to NUDT14 at the dimer interface between W34 of chain A and Y17 of chain B, enabling π–π stacking interactions. The phenoxy substituent engages in a hydrophobic interaction with L107 in chain B, whereas the purine core forms hydrogen bonds with D35 in chain A. (D) Superposition of NUDT5 and -14 active sites bound to compound **9**. NUDT5 in complex with **9** (black) is shown in gray tones (chain A—dark gray and chain B—light gray), while NUDT14 in complex with compound **9** (dark green) is in green tones (chain A—green and chain B—pale green).

To explore the potential of **9** as a dual inhibitor for NUDT5 and NUDT14, we next performed SPR with a purified human NUDT14 protein and confirmed potent direct binding (*K*_D_ ≈ 400 nM; Figure 4A). Since compound **9** is the first inhibitor of human NUDT14 to the best of our knowledge, we attempted cocrystallization to gain insights into the binding mode. Extensive crystallization trials yielded the first inhibitor-bound structure of NUDT14 in complex with **9** at a 1.82 Å resolution. Notably, this structure also revealed the hitherto unresolved conformation of the N-terminal domain in NUDT14, which consists of β-sheets intertwined with the NUDIX domain of the second subunit (Figure 4B). Similar to NUDT5, compound **9** occupies the active site of NUDT14 where the heterocyclic core is involved in π–π stacking interactions with W34 of chain A and Y17 of chain B (Figure 4C). In addition, the aminopyrimidine moiety is stabilized by hydrogen bonds with D35 and the aromatic ring of the phenoxy substituent interacts with L107 via hydrophobic interaction. By contrast, superimposition of the NUDT5 and NUDT14 cocrystal structures in complex with **9** (Figure 4D) revealed some notable differences: although the main π stacking residues of NUDT14 (Y17 and W34) appear to be conserved in NUDT5 (Y36 and W46, Figure S5), **9** is sandwiched between W46 and W28 in the NUDT5 active site. The hydrogen bond to D35 for NUDT14 is retained with E47 in the NUDT5 structure. In contrast to NUDT5 where the phenoxy group of the inhibitor engages with the hydrophobic side chain of R51, the NUDT14 structure revealed an interaction with L107 (Figure 4D). R51 is not conserved in NUDT14 and seems particularly important for the establishment of ligand H-bond interactions in NUDT5 as shown for ADP-ribose^17^ and TH5427 binding.^12^ This may also explain the exquisite selectivity of TH5427 (**6**) for NUDT5 over NUDT14.

**Figure 5:**
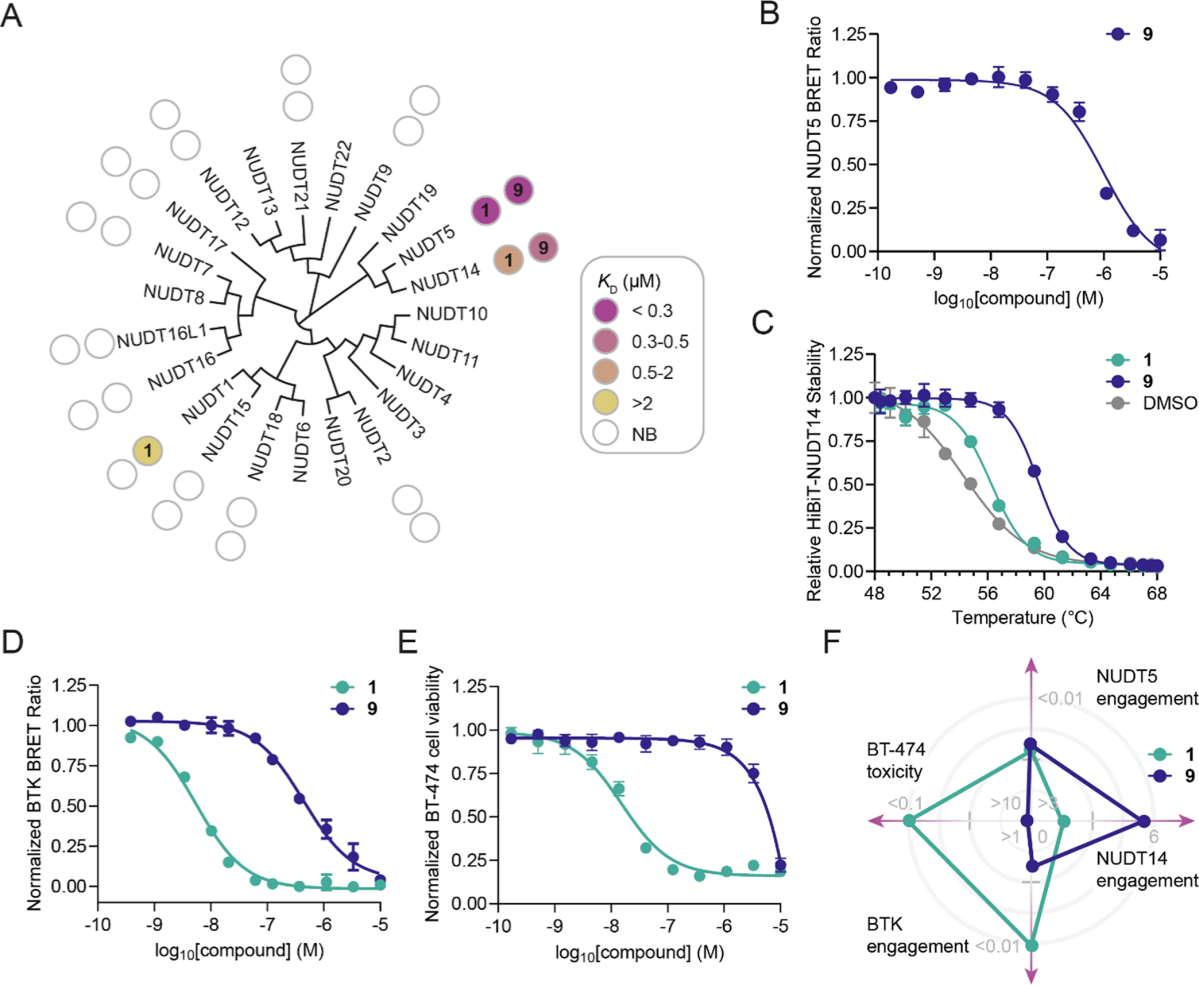
NUDIX selectivity, NUDT5 and NUDT14 cell target engagement and cell viability assay results. (A) NUDIX selectivity for compound **9** and ibrutinib (**1**) determined by SPR. NB, no binding (*K*_D_ > 100 μM) (*n* = 2). (B) NUDT5 NanoBRET TE assay result for the dual NUDT5/NUDT14 inhibitor **9** (EC_50_ = 1.08 ± 0.07 μM). (C) Compounds **1** (Δ*T*_m_ = 1.6 ± 0.3) and **9** (Δ*T*_m_ = 5.5 ± 0.3) stabilize HiBiT-NUDT14 measured by CETSA in intact HEK293 cells upon 30 μM treatment for 1 h. (D) BTK NanoBRET TE assay for evaluation of **1** (EC_50_ = 0.005 ± 0.001 μM) and **9** (EC_50_ = 0.377 ± 0.062 μM). (E) Cell viability assay of **1** (IC_50_ = 0.027 ± 0.011 μM) and **9** (>10 μM) in BT-474 cells after 72 h of incubation. (F) Radar chart summary of performance of **1** and **9** in different cell-based assays. Cell-based assay data are shown as mean ± SD and are based on three technical replicates or four technical replicates in the case of the HiBiT-NUDT14 CETSA. Graphs are representative of two independent biological replicates (*n* = 2).

We confirmed compound **9** as a selective NUDT5/NUDT14 inhibitor by extended biophysical SPR screening of the NUDIX family (Figure 5A and Table S1). Importantly, we validated NUDT5 cellular target engagement for **9** by NanoBRET (EC_50_ = 1.08 ± 0.07 μM; Figure 5B). To interrogate NUDT14 binding in live cells, we established a novel NUDT14 HiBiT CETSA (Figure S6). Compared to DMSO control, **9** led to a strong increase in NUDT14 thermal stability (Δ*T*_m_ = 5.5 ± 0.3; Figure 5C). Notably, **9** showed significantly lower potency in binding to BTK in cells (EC_50_ = 0.377 ± 0.062 μM) with an approximately 75-fold decrease compared to the parent inhibitor ibrutinib (**1**) (EC_50_ = 0.005 ± 0.001 μM; Figure 5D). This is consistent with our observation that compound **9** does not affect cell viability of BT-474 cells, which are highly sensitive toward **1**.^20^ Taken together, these data suggest a suitable window for the development of selective NUDT14 chemical probes (Figure 5F). Since both NUDT5 and NUDT14 are known to hydrolyze free ADP-ribose, we wondered whether **9** could affect global protein ADP-ribosylation in cells. We selected human osteosarcoma U2OS ARH3 KO cells as an established model^21^ and treated them with **9** in the absence or presence of a poly(ADP-ribose) glycohydrolase (PARG) inhibitor that promotes excessive ADP-ribosylation and allows visualization of transient long poly(ADP-ribose) chains on proteins. In both instances, we did not observe any significant effect on protein-bound ADP-ribose, suggesting that redundant or alternative mechanisms are able to supplement this pathway (Figure S7). This warrants further investigation, and we hope our work will thus inspire the development of additional tools.

## Conclusions

Increasing evidence in recent years suggests that, contrary to previous perceptions, covalent inhibitors can be optimized into targeted pharmacological agents with remarkable selectivity on a proteome-wide level.^22,23^ However, most studies are focused on approaches such as activity-based protein profiling (ABPP) interrogating the formation of covalent bonds between the inhibitor and its targets. Here, we show that noncovalent interactions of such compounds can still lead to potentially relevant off-target binding even outside of the respective target class. Re-engineering of the FDA-approved BTK inhibitor ibrutinib via a small SAR study enabled us to identify compound **9** as a new and potent dual NUDT5 and NUDT14 inhibitor. Using bespoke NanoBRET and HiBiT CETSA assays, we confirm inhibitor target engagement in live cells. Our chemoproteomic results further suggest that purine-mimetic NUDT5, and possibly NUDT14 inhibitors, can be optimized toward potent tool compounds with high selectivity. Compound **9** also enabled resolution of the first NUDT14 cocrystal structure, revealing insights into the so-far unresolved N-terminal domain of NUDT14.^24^ These results explain the strong selectivity observed for previous NUDT5 inhibitors, and when taken together with our other data, they should help pave the way for future chemical probe development. Importantly, NUDT14 is frequently expressed at elevated levels in cancer cells^3^ and has been described as a challenging protein to target primarily due to its smaller and more exposed active site.^25^ We anticipate that our work will facilitate further research into NUDT14 biology and help elucidate its role in physiology and disease.

## Experimental Section

### Materials and Methods

Commercial reagents and solvents were purchased from commercial suppliers and used without further purification. All reactions involving moisture-sensitive reagents were carried out under a nitrogen atmosphere using standard vacuum line techniques and dry solvents. An Elga DV 25 system was used for deionizing water. Thin-layer chromatography was performed on aluminum plates coated with 60 F254 silica gel. Plates were visualized using UV light (254 nm). Flash column chromatography was performed on a Biotage Isolera one flash column chromatography platform. ^1^H and ^13^C NMR spectra were obtained using Bruker NMR spectrometers (400 MHz). The proton and carbon chemical shift values are reported in parts per million (ppm, δ scale) downfield from tetramethylsilane (TMS) and the indicated solvent. NMR spectra were processed and analyzed using MestReNova software. Spin multiplicities are given as s (singlet), d (doublet), t (triplet), q (quartet), dd (doublet of doublets), dt (doublet of triplets), td (triplet of doublets), m (multiplet), and b (broad); coupling constants *J* are given in hertz (Hz) and signal area integration in natural numbers. LC-MS was performed with a Kinetex 5 μm EVO C18 100A 100 × 3.0 mm column on a Waters SFO and 515 HPLC pump and Waters Binary Gradient 2545 device using linear gradient of solvent A (93% water, 5% acetonitrile, and 2% of 0.5 M ammonium acetate pH 6.0) and solvent B (18% water, 80% acetonitrile, and 2% ammonium acetate pH 6.0), eluting at a flow rate of 2 mL/min: 5% B for 0.35 min, 5% B to 95% B for 1 min, 95% to 5% B for 0.1 min, and 5% B for 0.8 min. LC-MS was used as a measure of compound purity using either UV absorbance (Waters UV/visible Detector 2489), ELSD signal (Waters ELS Detector 2424), or ESI + TIC (SQ Detector 2). Preparative HPLC was performed on the same system with a Kinetex 5 μm EVO C18 100A 150 × 21.2 mm column using a linear gradient of solvent A over 20 min from 85 to 10%, eluting at a flow rate of 20 mL/min. LC-MS was acquired using Waters FractionLynx software and processed using MestReNova software. The purity for all final compounds was determined to be >95% by HPLC.

#### 3-Bromo-1-(1-methylpiperidin-4-yl)-1*H*-pyrazolo[3,4-*d*]pyrimidin-4-amine (**17a**)

To a solution of **16** (1.53 g, 7.13 mmol, 1.0 equiv) in THF (250 mL) were added 4-hydroxy-1-methylpiperidine (1.23 g, 10.7 mmol, 1.5 equiv), polymer-supported triphenylphosphine (2.30 mmol/g loading, 7.75 g, 17.8 mmol, 2.5 equiv), and DIAD (3.5 mL, 17.8 mmol, 2.5 equiv). The reaction mixture was stirred at rt overnight, then filtered, concentrated, and purified by automated flash chromatography (amine column), using a gradient elution of 0–10% methanol in DCM to provide **17a** as beige solid (767 mg, 2.47 mmol, 35% yield). ^1^H NMR (400 MHz, DMSO-*d*_6_) δ 8.19 (s, 1H), 4.58–4.50 (m, 1H), 2.88–2.85 (m, 2H), 2.20 (s, 3H), 2.13–2.01 (m, 4H), 1.86–1.81 (m, 2H); ^13^C NMR (101 MHz, DMSO-*d*_6_) δ 157.4, 156.5, 153.3, 116.8, 99.5, 54.3 (2C), 54.1, 45.8, 30.9 (2C); HPLC: *t*_R_ = 1.59 min (98.6%); MS (ESI+): *m*/*z* 311.234/313.219 [M + H]^+^.

#### 1-(1-Methylpiperidin-4-yl)-3-(4-phenoxyphenyl)-1*H*-pyrazolo[3,4-*d*]pyrimidin-4-amine (**9**)

A solution of **17a** (91.6 mg, 0.294 mmol, 1.0 equiv), 4-phenoxyphenylboronic acid (94.5 mg, 0.442 mmol, 1.5 equiv), and sodium carbonate (68.6 mg, 0.648 mmol, 2.2 equiv) in 1,4-dioxane/water (21/3 mL) was degassed. After addition of Pd(dppf)Cl_2_·DCM (13.2 mg, 0.0162 mmol, 0.055 equiv), the reaction mixture was again degassed and stirred overnight with vigorous stirring at 80 °C. After cooling to rt, the mixture was diluted in ethyl acetate, filtered through a short pad of celite, and concentrated *in vacuo*. The purification of the residue by automated flash chromatography (silica column) using a gradient elution of 0–10% methanol in DCM afforded **9** as a beige solid (81.0 mg, 0.202 mmol, 69% yield). ^1^H NMR (400 MHz, DMSO-*d*_6_) δ 8.23 (s, 1H), 7.65 (d, *J* = 8.5 Hz, 2H), 7.43 (t, *J* = 7.9 Hz, 2H), 7.20–7.11 (m, 5H), 4.68–4.60 (m, 1H), 2.92 (d, *J* = 10.5 Hz, 2H), 2.24 (s, 3H), 2.20–2.09 (m, 4H), 1.89 (d, *J* = 11.4, 2H); ^13^C NMR (101 MHz, DMSO-*d*_6_) δ 158.2, 157.1, 156.3, 155.5, 153.7, 142.8, 130.1 (2C), 130.0 (2C), 128.1, 123.8, 118.98 (2C), 118.96 (2C), 97.5, 54.4 (2C), 53.4, 45.7, 30.9 (2C); HPLC: *t*_R_ = 1.36 min (96.3%); MS (ESI+): *m*/*z* 401.615 [M + H]^+^.

#### 1-Methyl-3-(4-phenoxyphenyl)-1*H*-pyrazolo[3,4-*d*]pyrimidin-4-amine (**17b**)

A suspension of **16** (895 mg, 4.18 mmol, 1.0 equiv), methyl iodide (286 μL, 4.6 mmol, 1.1 equiv), and cesium carbonate (3.41 g, 10.5 mmol, 2.5 equiv) in dry DMF (50 mL) was stirred at rt overnight. After filtration, the solvent was removed under reduced pressure. Water was added to the reaction mixture followed by dilution with DCM. The aqueous layer was extracted with DCM, and the combined organic layers were dried over sodium sulfate, filtered, and concentrated *in vacuo*. The crude product was purified by automated flash chromatography (amine column), using a gradient elution of 0–10% methanol in DCM to afford **17b** as a colorless solid (223 mg, 0.978 mmol, 23% yield). ^1^H NMR (400 MHz, MeOD) δ 8.20 (s, 1H), 3.93 (s, 3H); ^13^C NMR (101 MHz, MeOD) δ 159.3, 157.7, 155.1, 118.7, 101.2, 34.4; HPLC: *t*_R_ = 1.09 min (97.1%); MS (ESI+): *m*/*z* 228.152/230.133 [M + H]^+^.

#### 1-Methyl-3-(4-phenoxyphenyl)-1*H*-pyrazolo[3,4-*d*]pyrimidin-4-amine (**10**)

A solution of **17b** (168 mg, 0.737 mmol, 1.0 equiv), 4-phenoxyphenylboronic acid (237 mg, 1.11 mmol, 1.5 equiv), and sodium carbonate (171 mg, 1.62 mmol, 2.2 equiv) in 1,4-dioxane/water (53/7.5 mL) was degassed. After addition of Pd(dppf)Cl_2_·DCM (33.1 mg, 0.041 mmol, 0.055 equiv), the reaction mixture was again degassed and stirred overnight with vigorous stirring at 80 °C. After cooling to rt, the mixture was diluted in ethyl acetate, filtered through a short pad of celite, and concentrated *in vacuo*. The purification of the residue by automated flash chromatography (silica column) using a gradient elution of 0–10% methanol in DCM afforded compound **10** as a beige solid (173 mg, 0.545 mmol, 74% yield). ^1^H NMR (400 MHz, MeOD) δ 8.25 (s, 1H), 7.66–7.63 (m, 2H), 7.41–7.37 (m, 2H), 7.18–7.07 (m, 5H), 4.00 (s, 3H); ^13^C NMR (101 MHz, MeOD) δ 160.0, 159.9, 157.9, 156.9, 155.2, 145.7, 131.2 (2C), 131.1 (2C), 128.7, 125.1, 120.6 (2C), 120.0 (2C), 99.0, 34.1; HPLC: *t*_R_ = 1.53 min (99.8%); MS (ESI+): *m*/*z* 318.569 [M + H]^+^.

#### 3-(4-Phenoxyphenyl)-1*H*-pyrazolo[3,4-*d*]pyrimidin-4-amine (**11**)

A solution of **16** (770 mg, 3.60 mmol, 1.0 equiv), 4-phenoxyphenylboronic acid (1.16 g, 5.40 mmol, 1.5 equiv), and sodium carbonate (840 mg, 7.92 mmol, 2.2 equiv) in 1,4-dioxane/water (258/36 mL) was degassed. After the addition of Pd(dppf)Cl_2_·DCM (162 mg, 0.198 mmol, 0.055 equiv), the reaction mixture was again degassed and stirred for 2 h with vigorous stirring at 80 °C. After cooling to rt, the mixture was diluted in ethyl acetate, filtered through a short pad of celite, and concentrated *in vacuo*. The crude product was purified by preparative HPLC to afford compound **11** as a colorless solid (340 mg, 1.12 mmol, 31% yield). ^1^H NMR (400 MHz, DMSO-*d*_6_) δ 13.55 (bs, 1H), 8.21 (s, 1H), 7.68–7.65 (m, 2H), 7.45–7.41 (m, 2H), 7.21–7.12 (m, 5H); ^13^C NMR (101 MHz, DMSO-*d*_6_) δ 158.1, 157.0, 156.3, 156.1, 155.8, 143.9, 130.1 (2C), 130.0 (2C), 128.4, 123.8, 119.0 (2C), 118.9 (2C), 96.9; HPLC: *t*_R_ = 1.38 min (97.8%); MS (ESI+): *m*/*z* 304.218 [M + H]^+^.

#### *tert*-Butyl 4-(4-Amino-1-(1-methylpiperidin-4-yl)-1*H*-pyrazolo[3,4-*d*]pyrimidin-3-yl)-3,6-dihydropyridine-1(2*H*)-carboxylate (**18a**)

A solution of **17a** (318 mg, 1.02 mmol, 1.0 equiv), *tert*-butyl 4-(4,4,5,5-tetramethyl-1,3,2-dioxaborolan-2-yl)-5-6-dihydropyridine-1(2*H*)-carboxylate (474 mg, 1.53 mmol, 1.5 equiv), and sodium carbonate (238 mg, 2.25 mmol, 2.2 equiv) in 1,4-dioxane/water (72/10 mL) was degassed. After the addition of Pd(dppf)Cl_2_·DCM (45.9 mg, 0.056 mmol, 0.055 equiv), the reaction mixture was again degassed and stirred overnight with vigorous stirring at 80 °C. After cooling to rt, the mixture was diluted in ethyl acetate, filtered through a short pad of celite, and concentrated *in vacuo*. The purification of the residue by automated flash chromatography (silica column) using a gradient elution of 0–10% methanol in DCM afforded compound **18a** as a dark brown solid (394 mg, 0.953 mmol, 93% yield). ^1^H NMR (400 MHz, MeOD) δ 8.19 (s, 1H), 6.12–6.10 (m, 1H), 4.84–4.82 (m, 1H), 4.16–4.15 (m, 2H), 3.69–3.66 (m, 2H), 3.26–3.21 (m, 2H), 2.71–2.65 (m, 2H), 2.63–2.59 (m, 2H), 2.55 (s, 3H), 2.44–2.34 (m, 2H), 2.07–2.02 (m, 2H), 1.50 (s, 9H); ^13^C NMR (101 MHz, MeOD) δ 159.9, 156.6, 156.4, 154.6, 146.3, 131.9, 127.6, 98.6, 81.4, 55.2 (2C), 53.6, 45.4, 44.3, 40.8, 31.2 (2C), 28.7 (3C), 28.6; HPLC: *t*_R_ = 1.21 min (97.2%); MS (ESI+): *m*/*z* 414.404 [M + H]^+^.

#### 1-(4-(4-Amino-1-(1-methylpiperidin-4-yl)-1*H*-pyrazolo[3,4-*d*]pyrimidin-3-yl)-3,6-dihydropyridin-1(2*H*)-yl)prop-2-en-1-one (**12**)

To a solution of **18a** (98.0 mg, 0.237 mmol, 1.0 equiv) in DCM (7 mL) was added 4 N hydrochloric acid in 1,4-dioxane (3 mL). The reaction mixture was stirred at rt for 2 h and then evaporated *in vacuo*. The residue was dissolved in DCM (15 mL), and then triethylamine (0.165 mL, 1.19 mmol, 5.0 equiv) and a solution of acryloyl chloride (23 μL, 0.284 mmol, 1.2 equiv) in DCM (1 mL) were added. The resulting mixture was stirred for 30 min. After evaporation of the solvent, the crude product was purified by automated flash chromatography (amine column), using a gradient elution of 0–10% of methanol in DCM to afford **12** as a beige solid (37.4 mg, 0.102 mmol, 43% yield). ^1^H NMR for both rotamers (400 MHz, MeOD) δ 8.19 (s, 1H), 6.90–6.76 (m, 1H), 6.26 (d, *J* = 16.8 Hz, 1H), 6.14 (d, *J* = 13.9 Hz, 1H), 5.79 (d, *J* = 10.7 Hz, 1H), 4.73–4.66 (m, 1H), 4.38 (dd, *J* = 18.1, 3.1 Hz, 2H), 3.92–3. 89 (m, 2H), 3.02 (d, *J* = 9.3 Hz, 2H), 2.82–2.73 (m, 2H), 2.34 (s, 3H), 2.30–2.23 (m, 4H), 1.94 (d, *J* = 10.3 Hz, 2H); ^1^H NMR for both rotamers (400 MHz, MeOD) 49 °C δ 8.19 (s, 1H), 6.80 (bs, 1H), 6.25 (dd, *J* = 17.1, 1.9 Hz, 1H), 6.15 (bs, 1H), 5.77 (dd, *J* = 10.7, 1.9 Hz, 1H), 4.73–4.67 (m, 1H), 4.37 (bs, 2H), 3.90 (t, *J* = 5.6 Hz, 2H), 3.04–3.01 (m, 2H), 2.79 (bs, 2H), 2.34 (s, 3H), 2.32–2.25 (m, 4H), 1.97–1.94 (m, 2H); ^13^C NMR for the major rotamer (101 MHz, MeOD) δ 168.0, 156.3, 154.5, 132.0, 129.1, 128.7, 127.2, 98.5, 55.7 (2C), 54.8, 46.2, 46.1, 44.0, 43.5, 40.0, 31.9 (2C), 29.3; HPLC: *t*_R_ = 1.01 min (98.3%); MS (ESI+): *m*/*z* 368.290 [M + H]^+^.

#### *tert*-Butyl 5-(4-Amino-1-(1-methylpiperidin-4-yl)-1*H*-pyrazolo[3,4-*d*]pyrimidin-3-yl)-3,6-dihydropyridine-1(2*H*)-carboxylate (**18b**)

A solution of **17a** (525 mg, 1.69 mmol, 1.0 equiv), *tert*-butyl 3-(4,4,5,5-tetramethyl-1,3,2-dioxaborolan-2-yl)-5-6-dihydropyridine-1(2*H*)-carboxylate (783 mg, 2.53 mmol, 1.5 equiv), and sodium carbonate (715 mg, 6.75 mmol, 4 equiv) in 1,4-dioxane/water (33/7 mL) was degassed. After the addition of Pd(dppf)Cl_2_·DCM (75.8 mg, 0.093 mmol, 0.055 equiv), the reaction mixture was again degassed and stirred overnight with vigorous stirring at 80 °C. After cooling to rt, the mixture was diluted in ethyl acetate, filtered through a short pad of celite, and concentrated *in vacuo*. The purification of the residue by automated flash chromatography (amine column) using a gradient elution of 0–100% ethyl acetate in cyclohexane afforded **18b** as a dark brown solid (637 mg, 1.54 mmol, 91% yield). ^1^H NMR (400 MHz, MeOD) δ 8.20 (s, 1H), 6.29–6.26 (m, 1H), 4.83–4.79 (m, 1H), 4.39–4.37 (m, 2H), 3.65–3.62 (m, 2H), 3.29–3.26 (m, 2H), 2.71–2.68 (m, 2H), 2.58 (s, 3H), 2.45–2.35 (m, 4H), 2.11–2.06 (m, 2H), 1.50 (s, 9H); ^13^C NMR (101 MHz, MeOD) δ 159.8, 156.7, 156.4, 154.5, 144.8, 131.2, 129.5, 98.8, 81.4, 55.2 (2C), 53.6, 45.7, 45.3, 41.6, 31.1 (2C), 28.3 (3C), 26.2; HPLC: *t*_R_ = 1.21 min (97.2%); MS (ESI+): *m*/*z* 414.312 [M + H]^+^.

#### 1-(5-(4-Amino-1-(1-methylpiperidin-4-yl)-1*H*-pyrazolo[3,4-*d*]pyrimidin-3-yl)-3,6-dihydropyridin-1(2*H*)-yl)prop-2-en-1-one (**13**)

To a solution of **18b** (107 mg, 0.259 mmol, 1.0 equiv) in DCM (1.3 mL) was added 4 N hydrochloric acid in 1,4-dioxane (0.5 mL). The reaction mixture was stirred at rt for 2 h and then evaporated *in vacuo*. The residue was dissolved in DCM (15 mL), and then triethylamine (0.180 mL, 1.294 mmol, 5.0 equiv) and a solution of acryloyl chloride (25 μL, 0.311 mmol, 1.2 equiv) in DCM (1 mL) were added. The resulting mixture was stirred for 30 min. After evaporation of the solvent, the crude product was purified by automated flash chromatography (amine column), using a gradient elution of 0–10% of methanol in DCM to afford compound **13** as a beige solid (49.0 mg, 0.133 mmol, 52% yield). ^1^H NMR for both rotamers (400 MHz, MeOD) δ 8.20 (s, 1H), 6.89–6.78 (m, 1H), 6.36–6.28 (m, 1H), 6.28–6.23 (m, 1H), 5.79 (dt, *J* = 10.6, 2.0 Hz, 1H), 4.74–4.67 (m, 1H), 4.63–4.58 (m, 2H), 3.85 (q, *J* = 5.6 Hz, 2H), 3.05–3.00 (m, 2H), 2.52–2.43 (m, 2H), 2.35 (s, 3H), 2.33–2.25 (m, 4H), 1.98–1.95 (m, 2H); ^13^C NMR for the major rotamer (101 MHz, MeOD) δ 168.1, 159.9, 156.3, 154.4, 144.4, 131.0, 129.9, 129.1, 128.7, 98.8, 55.6 (2C), 54.8, 46.1, 44.9, 43.6, 31.9 (2C), 27.1; HPLC: *t*_R_ = 1.12 min (98.5%); MS (ESI+): *m*/*z* 368.291 [M + H]^+^.

#### *tert*-Butyl (4-(4-Amino-1-(1-methylpiperidin-4-yl)-1*H*-pyrazolo[3,4-*d*]pyrimidin-3-yl)benzyl)carbamate (**18c**)

A solution of **17a** (385 mg, 1.24 mmol, 1.0 equiv), 4-[(*N*-Boc-amino)methyl]phenylboronic acid (466 mg, 1.86 mmol, 1.5 equiv), and sodium carbonate (289 mg, 2.72 mmol, 2.2 equiv) in 1,4-dioxane/water (33/7 mL) was degassed. After the addition of Pd(dppf)Cl_2_·DCM (55.6 mg, 0.068 mmol, 0.055 equiv), the reaction mixture was again degassed and stirred overnight with vigorous stirring at 80 °C. After cooling to rt, the mixture was diluted in ethyl acetate, filtered through a short pad of celite, and concentrated *in vacuo*. The purification of the residue by automated flash chromatography (silica column) using a gradient elution of 0–10% methanol in DCM afforded **18c** as a beige solid (432 mg, 0.987 mmol, 80% yield). ^1^H NMR (400 MHz, MeOD) δ 8.23 (s, 1H), 7.64 (d, *J* = 7.8 Hz, 2H), 7.46 (d, *J* = 7.8 Hz, 2H), 4.79–4.71 (m, 1H), 4.32 (s, 2H), 3.07–3.03 (m, 2H), 2.45–2.38 (m, 2H), 2.36 (s, 3H), 2.33–2.26 (m, 2H), 2.03–1.98 (m, 2H), 1.47 (s, 9H); ^13^C NMR (101 MHz, MeOD) δ 159.8, 158.7, 156.5, 154.7, 145.9, 142.1, 133.1, 129.7 (2C), 128.9 (2C), 99.3, 80.3, 55.7 (2C), 55.0, 46.1, 44.8, 32.0 (2C), 28.8 (3C); HPLC: *t*_R_ = 1.21 min (99.3%); MS (ESI+): *m*/*z* 438.308 [M + H]^+^.

#### *N*-(4-(4-Amino-1-(1-methylpiperidin-4-yl)-1*H*-pyrazolo[3,4-*d*]pyrimidin-3-yl)benzyl)acrylamide (**14**)

To a solution of **18c** (77.7 mg, 0.178 mmol, 1.0 equiv) in DCM (0.9 mL) was added 4 N hydrochloric acid in 1,4-dioxane (0.34 mL). The reaction mixture was stirred at rt for 2 h and then evaporated *in vacuo*. The residue was dissolved in DCM (10 mL); then, triethylamine (0.124 mL, 0.888 mmol, 5.0 equiv) and a solution of acryloyl chloride (17 μL, 0.213 mmol, 1.2 equiv) in DCM (1 mL) were added. The resulting mixture was stirred for 30 min. After evaporation of the solvent, the crude product was purified by preparative HPLC to afford compound **14** as a colorless solid (48.2 mg, 0.123 mmol, 69% yield). ^1^H NMR (400 MHz, MeOD) δ 8.25 (s, 1H), 7.65 (d, *J* = 8.0 Hz, 2H), 7.48 (d, *J* = 8.0 Hz, 2H), 6.37–6.24 (m, 2H), 5.71 (dd, *J* = 9.4, 2.6 Hz, 1H), 5.01–4.97 (m, 1H), 4.54 (s, 2H), 3.53–3.47 (m, 2H), 3.07–3.00 (m, 2H), 2.76 (s, 3H), 2.60–2.50 (m, 2H), 2.23–2.19 (m, 2H); ^13^C NMR (101 MHz, MeOD) δ 168.2, 159.8, 156.7, 155.0, 146.1, 141.2, 133.1, 131.9, 129.8 (2C), 129.4 (2C), 127.1, 99.3, 54.4 (2C), 52.6, 44.2, 43.9, 30.2 (2C); HPLC: *t*_R_ = 1.06 min (100%); MS (ESI+): *m*/*z* 392.524 [M + H]^+^.

#### *tert*-Butyl 4-(4-Amino-1-(1-methylpiperidin-4-yl)-1*H*-pyrazolo[3,4-*d*]pyrimidin-3-yl)piperidine-1-carboxylate (**19**)

A solution of **18a** (420 mg, 1.02 mmol, 1.0 equiv) in methanol (7 mL) was treated with wet 10% palladium on carbon (250 mg). The reaction mixture was stirred under hydrogen at 45 °C overnight. The mixture was filtered over a pad of celite and washed with methanol. The filtrate was concentrated *in vacuo* to provide **19** as a beige solid (391 mg, 0.941 mmol, 93% yield). ^1^H NMR (400 MHz, MeOD) δ 8.16 (s, 1H), 4.81–4.73 (m, 1H), 4.13–4.07 (m, 2H), 3.42–3.36 (m, 1H), 3.32–3.27 (m, 2H), 3.09 (bs, 2H), 2.75–2.68 (m, 2H), 2.60 (s, 3H), 2.47–2.36 (m, 2H), 2.08–2.03 (m, 2H), 2.00–1.96 (m, 2H), 1.80–1.70 (m, 2H), 1.47 (s, 9H); ^13^C NMR (101 MHz, MeOD) δ 159.6, 156.4, 156.2, 154.5, 149.6, 99.5, 80.9, 55.1, 53.0, 45.2, 44.8, 44.2, 36.2, 32.3, 30.9, 28.7; HPLC: *t*_R_ = 1.19 min (98.7%); MS (ESI+): *m*/*z* 416.343 [M + H]^+^.

#### 1-(4-(4-Amino-1-(1-methylpiperidin-4-yl)-1*H*-pyrazolo[3,4-*d*]pyrimidin-3-yl)piperidin-1-yl)prop-2-en-1-one (**15**)

To a solution of **19** (82.8 mg, 199 mmol, 1.0 equiv) in DCM (7 mL) was added 4 N hydrochloric acid in 1,4-dioxane (3 mL). The reaction mixture was stirred at rt for 2 h and then evaporated *in vacuo*. The residue was dissolved in DCM (15 mL), and then triethylamine (0.139 mL, 0.997 mmol, 5.0 equiv) and a solution of acryloyl chloride (19 μL, 0.239 mmol, 1.2 equiv) in DCM (1 mL) were added. The resulting mixture was stirred for 30 min. After evaporation of the solvent, the crude product was purified by preparative HPLC to afford **15** as a colorless solid (51.2 mg, 0.138 mmol, 70% yield). ^1^H NMR (400 MHz, MeOD) δ 8.17 (s, 1H), 6.82 (dd, *J* = 16.8, 10.7 Hz, 1H), 6.21 (dd, *J* = 16.8, 2.0 Hz, 1H), 5.75 (dd, *J* = 10.7, 2.0 Hz, 1H), 4.87–4.80 (m, 1H), 4.55–4.52 (m, 1H), 4.20–4.17 (m, 1H), 3.52–3.45 (m, 2H), 3.43–3.39 (m, 2H), 3.13–3.06 (m, 1H), 2.96–2.89 (m, 2H), 2.70 (s, 3H), 2.50–2.40 (m, 2H), 2.14–2.05 (m, 4H), 1.88–1.75 (m, 2H); ^13^C NMR (101 MHz, MeOD) δ 167.5, 159.8, 156.3, 154.7, 149.4, 129.2, 128.4, 99.6, 54.6 (2C), 52.4, 46.8, 44.4, 43.1, 36.2, 33.1, 32.1, 30.3 (2C); HPLC: *t*_R_ = 1.01 min (100%); MS (ESI+): *m*/*z* 370.570 [M + H]^+^.

#### *tert*-Butyl (4-(4-(7-((5-(3,4-Dichlorophenyl)-1,3,4-oxadiazol-2-yl)methyl)-1,3-dimethyl-2,6-dioxo-2,3,6,7-tetrahydro-1*H*-purin-8-yl)piperazin-1-yl)butyl)carbamate

To a mixture of TH5427 (**6**) (35 mg, 0.071 mmol, 1 equiv) in DMSO (1.2 mL), 4-(boc-amino) butyl bromide (36 mg, 0.142 mmol, 2 equiv) and DIPEA (37 μL, 0.214 mmol, 3 equiv) were added. The mixture was stirred at 80 °C for 2 h, and then water was added. The resulting precipitate was filtered off and then subsequently purified by preparative TLC (Si-35, DCM/methanol, 9/1) to afford the product as an off-white solid (14.6 mg, 0.022 mmol, 31% yield). ^1^H NMR (400 MHz, CDCl_3_) δ 8.09 (d, *J* = 2 Hz, 1H), 7.84 (dd, *J* = 8.4 Hz, 2.0 Hz, 1H), 7.58 (d, *J* = 8.4 Hz, 1H), 5.63 (s, 2H), 3.54 (s, 3H), 3.35 (s overlapping with m, 3H + 4H), 3.12–3.10 (m, 2H), 2.56 (bs, 4H), 2.41 (bs, 2H), 1.53 (bs, 4H), 1.42 (s, 9H).^13^C NMR (101 MHz, CDCl_3_) δ 164.0, 162.2, 156.6, 156.2, 154.9, 151.7, 148.0, 136.8, 133.9, 131.5, 128.9, 126.1, 123.2, 104.8, 79.2, 58.0, 52.4 (2C), 50.2, 40.5, 40.3 (2C), 30.0, 28.6 (3C), 28.0, 27.9, 24.1; HPLC: *t*_R_ = 1.70 min (99.4%), MS (ESI+): *m*/*z* 662.208/664.188 [M + H]^+^.

#### 4-(4-(7-((5-(3,4-Dichlorophenyl)-1,3,4-oxadiazol-2-yl)methyl)-1,3-dimethyl-2,6-dioxo-2,3,6,7-tetrahydro-1*H*-purin-8-yl)piperazin-1-yl)butan-1-aminium Trifluoroacetate, CBH-003 (**7**)

To a solution of *tert*-butyl (4-(4-(7-((5-(3,4-dichlorophenyl)-1,3,4-oxadiazol-2-yl)methyl)-1,3-dimethyl-2,6-dioxo-2,3,6,7-tetrahydro-1*H*-purin-8-yl)piperazin-1-yl)butyl)carbamate (14.6 mg, 0.022 mmol, 1 equiv) in DCM (1 mL) at 0 °C, trifluoroacetic acid (51 μL, 0.661 mmol, 30 equiv) was added and the solution was stirred at rt overnight. After concentration under reduced pressure, the expected product was obtained as a brown solid (15 mg, 0.022 mmol, quantitative). ^1^H NMR (400 MHz, MeOD) δ 8.16 (d, *J* = 2.0 Hz, 1H), 7.94 (dd, *J* = 8.4 Hz, 2.0 Hz, 1H), 7.74 (d, *J* = 8.40 Hz, 1H) 5.78 (s, 2H), 3.80–3.59 (m, 4H), 3.58–3.40 (s overlapping m, 3H + 4H), 3.29–3.24 (s overlapping m, 3H + 2H), 3.00 (t, *J* = 7.6 Hz, 2H) 1.92–1.84 (m, 2H), 1.79–1.71 (m, 2H); ^13^C NMR (100 MHz, MeOD) δ 165.2, 164.5, 156.2, 156.2, 153.1, 148.9, 137.5, 134.6, 132.8, 129.7, 127.5, 124.7, 106.4, 57.3, 52.2 (2C), 48.9 (2C), 41.2, 39.9, 30.2, 28.2, 25.5, 22.0; HPLC: *t*_R_ = 1.32 min (99.1%); MS (ESI+): *m*/*z* 562.165/564.025 [M + H]^+^.

#### *N*-(4-(4-(7-((5-(3,4-Dichlorophenyl)-1,3,4-oxadiazol-2-yl)methyl)-1,3-dimethyl-2,6-dioxo-2,3,6,7-tetrahydro-1*H*-purin-8-yl)piperazin-1-yl)butyl)-3-(5,5-difluoro-7-(1*H*-pyrrol-2-yl)-5H-5 λ^4^,6 λ^4^-dipyrrolo[1,2-c:2′,1′-*f*][1,3,2]diazaborinin-3-yl)propenamide, CBH-004 (**8**)

To a mixture of CBH-003 (**7**) (10.0 mg, 0.015 mmol, 1.0 equiv) in DMF (2.0 mL), DIPEA (8 μL, 0.044 mmol, 3.0 equiv) was added and the mixture was stirred for 10 min at rt. NanoBret 590SE (6.9 mg, 0.016 mmol, 1.1 equiv) was then added, and the mixture was stirred for 4 h in the dark at rt. Water was added, and the crude was purified by preparative HPLC to afford the product as a dark solid (2.3 mg, 0.002 mmol, 15% yield). ^1^H NMR (400 MHz, DMSO-*d*_6_) δ 8.15 (d, *J* = 8.2 Hz, 1H), 7.95–7.87 (m, 3H), 7.43 (bs, 1H), 7.37–7.33 (m, 2H), 7.28–7.27 (m, 1H), 7.17 (d, *J* = 7.2 Hz, 1H), 7.01 (d, *J* = 7.0 Hz, 1H), 6.35–6.33 (m, 2H), 5.68 (s, 2H), 3.40 (s, 3H), 3.38–3.37 (m, 2H), 3.27–3.24 (m, 6H), 3.15 (s, 3H), 3.14–3.11 (2H, m), 3.08–3.07 (2H, m), 2.44–2.42 (m, 4H), 2.33–2.28 (m, 2H), 1.43–1.39 (m, 2H). HPLC: *t*_R_ = 1.71 min (98.1%); MS (ESI+): *m*/*z* 874.405/876.345 [M + H]^+^.

### Protein Expression and Purification

Genes encoding NUDT5 (NCBI reference NP_054861, residues 1-208) and NUDT14 (NCBI reference NP_803877, residues 1-222) were amplified by PCR (primer information Table S3). The amplified PCR products were cloned into expression vector pNIC28-Bsa4^26^ by ligation-independent cloning. The resulting constructs express the proteins fused with an N-terminal 6× histidine tag followed by a TEV protease cleavage site. All plasmids were transformed into *Escherichia coli* (DE3), and protein expression was induced at a cell density of OD_600_ = 0.8 with 0.3 or 0.5 mM isopropyl-d-1-thiogalactopyranoside (IPTG) at 18 °C overnight for NUDT14 and NUDT5, respectively. Cell pellets were resuspended in lysis buffer containing 20 mM HEPES, 500 mM NaCl, 5% glycerol, and 0.5 mM TCEP pH 7.5 supplemented with lysozyme, protease inhibitor cocktail (Set III, EDTA-free, Calbiochem), and benzonase (2 μL of 10,000 U/μL, Merck). Cells were then lysed using an ultrasonic cell disruptor Vibra-Cell (Sonics). The clarified supernatant was injected into a Ni-NTA affinity chromatography column (HisTrap Crude FF GE Healthcare) and eluted with a gradient of increasing imidazole concentrations. The affinity-purified proteins were further incubated with TEV protease overnight at 4 °C and purified by reverse nickel affinity. Proteins were further purified by size-exclusion chromatography (16/600 Superdex 75 PG, GE Healthcare) in buffer (20 mM HEPES, pH 7.5, 500 mM NaCl, 0.5 mM TCEP). Protein purity was analyzed by sodium dodecyl sulfate polyacrylamide gel electrophoresis (SDS-PAGE), and proteins were concentrated to 20 mg/mL for NUDT14 and 26 mg/mL for NUDT5 using 10,000 MWCO Vivaspin concentrators (Vivascience).

### Catalytic Assays

Inhibition activities of the compounds were determined using AMP-Glo system (Promega). The compounds were diluted from 50 μM to 0 μM in a final reaction containing 20 mM HEPES, 100 mM NaCl, 0.5 mM TCEP, 1 mM MgCl_2_, and 0.1% BSA, pH 7.4. The reactions were performed in 1536-well plates in a 2 μL reaction volume with 1 nM of NUDT5 or NUDT14, and 10 μM of ADPr as the substrate. The final DMSO concentration was 1% for all reactions. NUDT5 reactions were incubated for 20 min, while NUDT14 reactions were carried out for 1 h at rt. The reactions were stopped by adding 2 μL of AMP-Glo I. The stop solution is supplemented with 25 μM of a compound, PubChem CID 16339098, in order to stop enzyme activity completely. The reactions were further incubated with 4 μL of the detection solution for 1 h at rt. Luminescence signals were then measured in a PHERAstar FSX plate reader. Experiments were done in triplicate sets, and data were analyzed by GraphPad Prism 9. Inhibitor dose–response data were normalized to reactions containing vehicle only (1% v/v DMSO, 100% activity) and reactions containing 500 nM TH5427 (**6**) (1% v/v DMSO, 0% activity). Data are represented as the mean ± SD of two independent biological replicates.

### NanoBRET Target Engagement Assay

NanoBRET Target Engagement Assays were done by using the NanoBRET Target Engagement Intracellular Kinase Assay kit (Promega) according to the manufacturer’s instructions. In brief, 200,000 HEK293 cells/mL were reverse-transfected with either N-terminal NanoLuc tagged NUDT5 or C-terminal NanoLuc tagged BTK plasmid using FuGENE transfection reagent (Promega). After 24 h of incubation, cells were trypsinized and adjusted to 200,000 cells/mL in assay medium (Opti-MEM + 4% FBS). 2.5 nM CBH-004 and 0.5 μM tracer-05 (Promega) were used for the NUDT5 and BTK assay, respectively. Tracers were spiked into the cell suspension, and 40 μL of cells were seeded into each well of a white PP 384-well plate (781207, Greiner) containing the compounds prepared by an Echo Liquid Handler. After 2 h of incubation, a mix of substrate and extracellular NanoLuc inhibitor in assay medium were added into each well. Donor and acceptor signals were measured in a PHERAstar FSX or FS plate reader and analyzed by GraphPad Prism (v.9 or v.10). For this, the acceptor/donor ratio was calculated and the no tracer background signal was subtracted and multiplied by 1000 to yield mBRET units. Data were then normalized to DMSO.

### HiBiT Cellular Thermal Shift Assay

To determine NUDT14 engagement in intact cells, a HiBiT cellular thermal shift assay was set up.^27^ The Nano-Glo HiBiT lytic detection system was used following the manufacturer’s instructions (Promega). In brief, 400,000 HEK293 cells/mL were reverse-transfected with N-terminal HiBiT tagged NUDT14 using FuGENE transfection reagent (Promega). After 24 h of incubation, cells were trypsinized and resuspended at 400,000 cells/mL in assay medium. The cells were divided into compound and untreated (DMSO) groups and treated accordingly. Ten μL of cells were seeded into each well in a 384-well white PCR plate and incubated for 1 h. After incubation, the samples were heated in a PCR thermocycler at the desired temperature gradient. The plate was allowed to stand for 5 min at rt, and 10 μL of Nano-Glo HiBiT lytic mix was added into each well. The signals were measured in a PHERAstar FSX and analyzed by GraphPad Prism (v.9 or v.10).

### Cell Viability Assay

BT-474 cells were cultivated in DMEM containing 10% FBS at 37 °C and 5% CO_2_. Compounds were dispensed on a white PP 384-well plate (781207, Greiner) using an Echo Liquid Handler. Cells were trypsinized, counted, and diluted to 25,000 cells/mL in culture medium. 40 μL of cell suspension was seeded to each well, and plates were sealed with a breathable film and incubated for 72 h. Cell viability was determined using the CellTiter-Glo reagent following the manufacturer’s instructions (Promega). Luminescence signals were measured in a PHERAstar FS or FSX and analyzed by GraphPad Prism (v.9 or v.10). Data were normalized to DMSO.

### Western Blotting

For the detection of ADP-ribosylation changes by Western blotting, the cell deficient in ADP-ribosyl hydrolases ARH3 and PARG was used, which provides a sensitized background and allows the detection of significant ADP-ribosylation without external DNA damage.^21^ TH5427 (**6**) and **9** were added at 5 μM. Cells were lysed with Triton X-100 lysis buffer (50 mM tris-HCl pH 8.0, 100 mM NaCl, 1% Triton X-100) supplemented with 5 mM MgCl_2_, protease and phosphatase inhibitors (Roche), 1 μM Olaparib (Cayman Chemical), and 1 μM PARGi PDD00017273 (Sigma-Aldrich) at 4 °C. The lysates were incubated with 0.1% Benzonase (Sigma-Aldrich) for 30 min at 4 °C, centrifuged at 15,000 rpm for 15 min, and supernatants were collected. Proteins were heated to 90 °C in a 1× NuPAGE LDS sample buffer (Invitrogen) with DTT (Sigma-Aldrich) for 5 min. Samples were resolved on NuPAGE Novex 4–12% Bis-Tris gels (Invitrogen) and transferred onto nitrocellulose membranes (Bio-Rad) using Trans-Blot Turbo Transfer System (Bio-Rad). The membranes were blocked in phosphate-buffered saline (PBS) buffer with 5% nonfat dried milk and 0.1% Tween-20 for 1 h at rt and incubated overnight with primary antibodies at 4 °C. Primary antibodies were used at the following concentrations: PARP1 (556494, 1:5000), mono/poly-ADPr (83732, 1:1500), histone H3 (07-690, 1:50,000), and NUDT5 (ab129172, 1:1000). Membranes were incubated with peroxidase-conjugated secondary antibody (antimouse, Agilent, 1:2000, antirabbit, Agilent, 1:2000) or fluorophore-conjugated secondary antibody (Alexa Fluor 680 antirabbit IgG, 1:5000) for 1 h. Blots were developed using ECL (Invitrogen) and analyzed by exposing to films or imaged by a LI-COR imaging system.

### Binding Affinity Determination

Surface plasmon resonance (SPR) experiments were performed using a Biacore S200 instrument at 25 °C. Samples were immobilized on a CM5 chip using amine-coupling method according to the manufacturer’s instructions using a running buffer containing 50 mM HEPES, 150 mM NaCl, 2 mM MgCl_2_, and 0.05% Tween-20, pH 7.5. The binding affinity assay was run with 2% DMSO in the running buffer at a flow rate of 30 μL/min with a 60 s association and 200 s dissociation times. All of the assays were performed twice, and binding affinity was calculated using BIAevaluation software.

### Crystallization

For cocrystallization, NUDT5 (26 mg/mL) was mixed with ibrutinib (**1**) at 2.5 mM in the presence of 25 mM MgCl_2_ and incubated for 1 h on ice prior to crystallization trials. Crystallization screens were prepared using the sitting-drop method with a precipitant solution (1:1). Cocrystals were grown in a buffer containing 0.1 M tris pH 8.0, 33% PEG4000, 0.2 M MgCl_2_. For cocrystallization, NUDT14 (20 mg/mL) was mixed with compound **9** at a molar ratio of 1:5 and incubated for 2 h on ice prior to crystallization trials. Crystallization screens were prepared using the sitting-drop method with a precipitant solution (1:1). NUDT5 cocrystals with compound **9** were grown in a buffer containing 0.2 M ammonium acetate, 0.1 M citrate pH 5.5, and 30% PEG4000, while NUDT14 cocrystals with compound **9** were formed in a buffer containing 0.2 M MgCl_2_, 0.1 M bis-tris pH 6.5, and 25% PEG3350. Crystallization trials for NUDT5 and NUDT14 were performed at 18 and 4 °C, respectively. Crystals were cryoprotected with the crystallization condition containing 20% glycerol and flash-frozen in liquid nitrogen.

### Data Collection, Structure Determination, and Refinement

X-ray data sets were collected at Diamond Light Source (Harwell, U.K.) on the I03 beamline using an Eiger2 XE 16 M detector or on beamline I04-1 using a Pilatus 6 M detector. Data processing and scaling were performed in XDS^28^ and Aimless.^29^ The structures of NUDT5—ibrutinib (**1**), NUDT5—compound **9**, and NUDT14—compound **9** were solved in Molecular replacement in Phaser^30^ using PDB 2DSB(17) for NUDT5 and PDB 3Q91(24) for NUDT14 as the search models. The models were further built in Coot^31^ and refined in REFMAC5.^32^

### RapidFire–Intact Protein Mass Spectrometry

Agilent RapidFire High-throughput Mass Spectrometry (RapidFire MS) system was used to observe adduct formation with intact proteins. Specifically, 2 μM of NUDT5 or NUDT14 was incubated with 100 μM of compounds for 2 h. 50 μL was injected into an Agilent RapidFire C4 cartridge. Pump 1 was placed in mobile solution A that contains 0.1% formic acid in water, and Pumps 2 and 3 were in mobile phase B containing 0.1% formic acid and 85% acetonitrile. Data analysis was performed using Agilent MassHunter Qualitative Analysis B.07.00 software.

### Chemoproteomic Pulldowns

T-47D cells were grown in RPMI medium supplemented with 10% FBS and 1× GlutaMAX at 37 °C and 5% CO_2_. When reaching about 80% confluency, cells were harvested, washed with PBS, and frozen at −80 °C. Lysates were generated by adding 3× pellet volume of Buffer A (50 mM tris pH 7.5, 0.8% v/v NP-40, 5% v/v glycerol, 1.5 mM MgCl_2_, 100 mM NaCl, 25 mM NaF, 1 mM Na_3_VO_4_, 1 mM PMSF, 1 mM DTT, 10 μg/mL TLCK, 1 μg/mL Leupeptin, 1 μg/mL Aprotinin, 1 μg/mL soybean trypsin) supplemented with 1 μL/mL of benzonase. To facilitate lysis, crude lysate was drawn 10 times with a needle and incubated for 30 min on ice before clearing by centrifugation.

Chemical pulldowns were performed as previously described.^33^ In brief, the affinity matrix was generated by coupling CBH-003 (**7**) to NHS-activated sepharose beads (Cytiva, #17090601) at a final concentration of 0.5 mM. The lysate (5 mg in 300 μL per condition) was incubated with 20 μM TH5427 (**6**) or DMSO for 30 min at 4 °C before applying it to the affinity beads (50 μL per condition) for 2 h at 4 °C. Affinity matrices were washed 4 times with 1 mL Buffer A, and proteins were eluted 2× with 50 μL of 2× Laemmli buffer containing 25 mM DTT in PBS for 5 min at 95 °C.

Eluents (70 μL) were diluted in 0.1 M tris (pH 7.8) to 200 μL, reduced with DTT (5 mM final concentration) for 30 min at rt, and alkylated with iodoacetamide (20 mM final concentration) for 30 min in the dark. Protein was precipitated by the sequential addition of MeOH (600 μL), CHCl_3_ (150 μL), and H_2_O (450 μL), pelleted (17,000*g*, 5 min), washed with further MeOH (2 × 600 μL), and repelleted. Air-dried pellets were resuspended in 6 M urea (pH 7.8, 50 μL) by vortexing; the resulting protein solution was diluted with 250 μL of H_2_O and then incubated with trypsin (1 μg) overnight at 37 °C. The digests were acidified with formic acid (1% (v/v) final concentration), desalted using SOLA HRP SPE Cartridges (Thermo Fisher), eluting with 69% (v/v) MeCN, 0.1% (v/v) formic acid in H_2_O (600 μL), and dried *in vacuo*. The dried peptides were stored at −20 °C before resuspension in 2% (v/v) MeCN and 0.1% (v/v) formic acid in H_2_O (20 μL) for LC-MS/MS analysis.

### LC-MS/MS Data Acquisition and Analysis

Digested samples were analyzed by nano-UPLC-MS/MS using a Dionex Ultimate 3000 nano-UPLC fitted with an EASY spray column (75 μm × 500 mm, 2 μm particle size, Thermo Scientific), coupled to an Orbitrap Q Exactive instrument. A 60 min gradient of 0.1% (v/v) formic acid in 5% (v/v) DMSO to 0.1% (v/v) formic acid with 35% (v/v) acetonitrile in 5% (v/v) DMSO at a flow rate of 250 nL·min^–1^ was used. The instrument was operated in a data-dependent mode, with survey scans acquired at a resolution of 70,000 at 200 *m*/*z* and the 15 most abundant precursors selected for HCD fragmentation with an AGC target of 1 × 10^5^ ions. Raw data were searched in using MSFragger v.3.7^34^ IonQuant 1.8.10^35^ and Philosopher 4.8.0^36^ within Fragpipe v.19.1. against the human proteome FASTA file (UniProt UP000005640, downloaded 05.08.21) with added decoy and contaminants. The “LFQ-MBR” workflow was enabled for label-free quantification (LFQ) and match between runs (MBR) with standard settings for identification and quantification. For the *in silico* digest, trypsin was selected, allowing 2 missed cleavages, and methionine oxidation and cysteine carbamidomethylation were kept as variable modifications. Data were further processed using Perseus version 2.0.9.0. MaxLFQ intensities were log_2_-transformed, experimental replicates were grouped, and proteins were filtered out when detected less than 3 out of 4 times. Conditions were normalized by median subtraction. Missing values were imputed with a constant (−3) and the fold change and Student’s *t* test *p*-value between competition and DMSO group. Principal component analysis was performed with the Benjamini–Hochberg FDR of 0.05. Plots were created in R studio version 4.1.1.

## Supporting Information Available

Chemical proteomics results; NUDT5 NanoBRET TE assay; mass spectrometry analysis of ibrutinib analogues with NUDT5; mass spectrometry analysis of ibrutinib analogues with NUDT14; multiple sequence alignment of NUDT14 and NUDT5; NUDIX selectivity for compound **9** and ibrutinib (**1**) determined by SPR; setup of HiBiT-NUDT14 CETSA; assessment of protein ADP-ribosylation; NMR spectra and LC-MS chromatograms; X-ray data collection and refinement statistics; and primers for cloning (PDF)
Molecular formula strings (CSV)

## Abbreviations Used

DCM: dichloromethane
DIAD: diisopropyl azodicarboxylate
DIPEA: *N*,*N*-diisopropylethylamine
DMF: *N*,*N*-dimethylformamide
DMSO: dimethyl sulfoxide
dppf: [1,1′-bis(diphenylphosphino)ferrocene]
MS: mass spectrometry
Pin: pinacol boronic ester
PS: polymer-supported
THF: tetrahydrofuran
TFA: trifluoroacetic acid

## References

[ref1] Bessman M. J.; Frick D. N.; O’Handley S. F. The MutT proteins or ″Nudix″ hydrolases, a family of versatile, widely distributed, ″housecleaning″ enzymes. J. Biol. Chem. 1996, 271 (41), 25059–25062. 10.1074/jbc.271.41.25059. 8810257

[ref2] Mildvan A. S.; Xia Z.; Azurmendi H. F.; Saraswat V.; Legler P. M.; Massiah M. A.; Gabelli S. B.; Bianchet M. A.; Kang L. W.; Amzel L. M. Structures and mechanisms of Nudix hydrolases. Arch. Biochem. Biophys. 2005, 433 (1), 129–143. 10.1016/j.abb.2004.08.017. 15581572

[ref3] Carreras-Puigvert J.; Zitnik M.; Jemth A. S.; Carter M.; Unterlass J. E.; Hallstrom B.; Loseva O.; Karem Z.; Calderon-Montano J. M.; Lindskog C.; et al. A comprehensive structural, biochemical and biological profiling of the human NUDIX hydrolase family. Nat. Commun. 2017, 8 (1), 1541 10.1038/s41467-017-01642-w. 29142246 PMC5688067

[ref4] Arimori T.; Tamaoki H.; Nakamura T.; Kamiya H.; Ikemizu S.; Takagi Y.; Ishibashi T.; Harashima H.; Sekiguchi M.; Yamagata Y. Diverse substrate recognition and hydrolysis mechanisms of human NUDT5. Nucleic Acids Res. 2011, 39 (20), 8972–8983. 10.1093/nar/gkr575. 21768126 PMC3203587

[ref5] Ito R.; Sekiguchi M.; Setoyama D.; Nakatsu Y.; Yamagata Y.; Hayakawa H. Cleavage of oxidized guanine nucleotide and ADP sugar by human NUDT5 protein. J. Biochem. 2011, 149 (6), 731–738. 10.1093/jb/mvr028. 21389046

[ref6] Gupte R.; Liu Z.; Kraus W. L. PARPs and ADP-ribosylation: recent advances linking molecular functions to biological outcomes. Genes Dev. 2017, 31 (2), 101–126. 10.1101/gad.291518.116. 28202539 PMC5322727

[ref7] Palazzo L.; Mikoc A.; Ahel I. ADP-ribosylation: new facets of an ancient modification. FEBS J. 2017, 284 (18), 2932–2946. 10.1111/febs.14078. 28383827 PMC7163968

[ref8] Perraud A. L.; Shen B.; Dunn C. A.; Rippe K.; Smith M. K.; Bessman M. J.; Stoddard B. L.; Scharenberg A. M. NUDT9, a member of the Nudix hydrolase family, is an evolutionarily conserved mitochondrial ADP-ribose pyrophosphatase. J. Biol. Chem. 2003, 278 (3), 1794–1801. 10.1074/jbc.M205601200. 12427752

[ref9] Formentini L.; Macchiarulo A.; Cipriani G.; Camaioni E.; Rapizzi E.; Pellicciari R.; Moroni F.; Chiarugi A. Poly(ADP-ribose) catabolism triggers AMP-dependent mitochondrial energy failure. J. Biol. Chem. 2009, 284 (26), 17668–17676. 10.1074/jbc.M109.002931. 19411252 PMC2719406

[ref10] Yang H.; Slupska M. M.; Wei Y. F.; Tai J. H.; Luther W. M.; Xia Y. R.; Shih D. M.; Chiang J. H.; Baikalov C.; Fitz-Gibbon S.; et al. Cloning and characterization of a new member of the Nudix hydrolases from human and mouse. J. Biol. Chem. 2000, 275 (12), 8844–8853. 10.1074/jbc.275.12.8844. 10722730

[ref11] Wright R. H. G.; Lioutas A.; Le Dily F.; Soronellas D.; Pohl A.; Bonet J.; Nacht A. S.; Samino S.; Font-Mateu J.; Vicent G. P.; et al. ADP-ribose-derived nuclear ATP synthesis by NUDIX5 is required for chromatin remodeling. Science 2016, 352 (6290), 1221–1225. 10.1126/science.aad9335. 27257257

[ref12] Page B. D. G.; Valerie N. C. K.; Wright R. H. G.; Wallner O.; Isaksson R.; Carter M.; Rudd S. G.; Loseva O.; Jemth A. S.; Almlof I.; et al. Targeted NUDT5 inhibitors block hormone signaling in breast cancer cells. Nat. Commun. 2018, 9 (1), 250 10.1038/s41467-017-02293-7. 29343827 PMC5772648

[ref13] Pickup K. E.; Pardow F.; Carbonell-Caballero J.; Lioutas A.; Villanueva-Canas J. L.; Wright R. H. G.; Beato M. Expression of Oncogenic Drivers in 3D Cell Culture Depends on Nuclear ATP Synthesis by NUDT5. Cancers 2019, 11 (9), 1337 10.3390/cancers11091337. 31510016 PMC6770457

[ref14] Huber K. V. M.; Salah E.; Radic B.; Gridling M.; Elkins J. M.; Stukalov A.; Jemth A. S.; Gokturk C.; Sanjiv K.; Stromberg K.; et al. Stereospecific targeting of MTH1 by (S)-crizotinib as an anticancer strategy. Nature 2014, 508 (7495), 222–227. 10.1038/nature13194. 24695225 PMC4150021

[ref15] Hantschel O. Unexpected off-targets and paradoxical pathway activation by kinase inhibitors. ACS Chem. Biol. 2015, 10 (1), 234–245. 10.1021/cb500886n. 25531586

[ref16] Trapp J.; Jochum A.; Meier R.; Saunders L.; Marshall B.; Kunick C.; Verdin E.; Goekjian P.; Sippl W.; Jung M. Adenosine mimetics as inhibitors of NAD+-dependent histone deacetylases, from kinase to sirtuin inhibition. J. Med. Chem. 2006, 49 (25), 7307–7316. 10.1021/jm060118b. 17149860

[ref17] Zha M.; Zhong C.; Peng Y.; Hu H.; Ding J. Crystal structures of human NUDT5 reveal insights into the structural basis of the substrate specificity. J. Mol. Biol. 2006, 364 (5), 1021–1033. 10.1016/j.jmb.2006.09.078. 17052728

[ref18] Food and Drug Administration (FDA). Highlights of Prescribing Information. IMBRUVICA TM (ibrutinib) capsules, for oral use. 2019. https://www.imbruvica.com/files/prescribing-information.pdf.

[ref19] Dale N. C.; Johnstone E. K.; White C. W.; Pfleger K. D. NanoBRET: the bright future of proximity-based assays. Front. Bioeng. Biotechnol. 2019, 7, 56 10.3389/fbioe.2019.00056. 30972335 PMC6443706

[ref20] Chen J.; Kinoshita T.; Sukbuntherng J.; Chang B. Y.; Elias L. Ibrutinib Inhibits ERBB Receptor Tyrosine Kinases and HER2-Amplified Breast Cancer Cell Growth. Mol. Cancer Ther. 2016, 15 (12), 2835–2844. 10.1158/1535-7163.MCT-15-0923. 27678331

[ref21] Prokhorova E.; Agnew T.; Wondisford A. R.; Tellier M.; Kaminski N.; Beijer D.; Holder J.; Groslambert J.; Suskiewicz M. J.; Zhu K.; et al. Unrestrained poly-ADP-ribosylation provides insights into chromatin regulation and human disease. Mol. Cell 2021, 81 (12), 2640–2655. e2648. 10.1016/j.molcel.2021.04.028. 34019811 PMC8221567

[ref22] Martin J. G.; Ward J. A.; Feyertag F.; Zhang L.; Couvertier S.; Guckian K.; Huber K. V. M.; Johnson D. S. Chemoproteomic Profiling of Covalent XPO1 Inhibitors to Assess Target Engagement and Selectivity. ChemBioChem 2021, 22 (12), 2116–2123. 10.1002/cbic.202100038. 33887086

[ref23] Gehringer M.; Laufer S. A. Emerging and Re-Emerging Warheads for Targeted Covalent Inhibitors: Applications in Medicinal Chemistry and Chemical Biology. J. Med. Chem. 2019, 62 (12), 5673–5724. 10.1021/acs.jmedchem.8b01153. 30565923

[ref24] Tresaugues L.; Siponen M. I.; Arrowsmith C. H.; Berglund H.; Bountra C.; Collins R.; Edwards A. M.; Ekblad T.; Flodin S.; Flores A.; Graslund S.; Hammarstrom M.; Johansson I.; Karlberg T.; Kol S.; Kotenyova T.; Kouznetsova E.; Moche M.; Nyman T.; Persson C.; Schuler H.; Schutz P.; Thorsell A. G.; Van Der Berg S.; Wahlberg E.; Weigelt J.; Welin M.; Nordlund P.; Structural Genomics Consortium (SGC). Crystal Structure of Human Uridine Diphosphate Glucose Pyrophosphatase (NUDT14). 2011. 10.2210/pdb3q91/pdb.

[ref25] Michel M.; Homan E. J.; Wiita E.; Pedersen K.; Almlof I.; Gustavsson A. L.; Lundback T.; Helleday T.; Warpman Berglund U. In silico Druggability Assessment of the NUDIX Hydrolase Protein Family as a Workflow for Target Prioritization. Front Chem. 2020, 8, 443 10.3389/fchem.2020.00443. 32548091 PMC7274155

[ref26] Savitsky P.; Bray J.; Cooper C. D.; Marsden B. D.; Mahajan P.; Burgess-Brown N. A.; Gileadi O. High-throughput production of human proteins for crystallization: the SGC experience. J. Struct. Biol. 2010, 172 (1), 3–13. 10.1016/j.jsb.2010.06.008. 20541610 PMC2938586

[ref27] Ramachandran S.; Szewczyk M.; Barghout S. H.; Ciulli A.; Barsyte-Lovejoy D.; Vu V. HiBiT Cellular Thermal Shift Assay (HiBiT CETSA). In Chemogenomics: Methods and Protocols; Springer, 2023; pp 149–165. 10.1007/978-1-0716-3397-7_11 37558947

[ref28] Kabsch W. Xds. Acta Crystallogr., Sect. D: Biol. Crystallogr. 2010, 66 (Pt 2), 125–132. 10.1107/S0907444909047337. 20124692 PMC2815665

[ref29] Evans P. R.; Murshudov G. N. How good are my data and what is the resolution?. Acta Crystallogr., Sect. D: Biol. Crystallogr. 2013, 69 (Pt 7), 1204–1214. 10.1107/S0907444913000061. 23793146 PMC3689523

[ref30] McCoy A. J.; Grosse-Kunstleve R. W.; Adams P. D.; Winn M. D.; Storoni L. C.; Read R. J. Phaser crystallographic software. J. Appl. Crystallogr. 2007, 40 (Pt 4), 658–674. 10.1107/S0021889807021206. 19461840 PMC2483472

[ref31] Emsley P.; Lohkamp B.; Scott W. G.; Cowtan K. Features and development of Coot. Acta Crystallogr., Sect. D: Biol. Crystallogr. 2010, 66 (Pt 4), 486–501. 10.1107/S0907444910007493. 20383002 PMC2852313

[ref32] Murshudov G. N.; Skubak P.; Lebedev A. A.; Pannu N. S.; Steiner R. A.; Nicholls R. A.; Winn M. D.; Long F.; Vagin A. A. REFMAC5 for the refinement of macromolecular crystal structures. Acta Crystallogr., Sect. D: Biol. Crystallogr. 2011, 67 (Pt 4), 355–367. 10.1107/S0907444911001314. 21460454 PMC3069751

[ref33] Huber K. V. M.; Superti-Furga G. Profiling of small molecules by chemical proteomics. In Proteomics in Systems Biology: Methods and Protocols; Springer: New York, 2016; Vol. 1394, pp 211–218 10.1007/978-1-4939-3341-9_15. 26700051

[ref34] Kong A. T.; Leprevost F. V.; Avtonomov D. M.; Mellacheruvu D.; Nesvizhskii A. I. MSFragger: ultrafast and comprehensive peptide identification in mass spectrometry–based proteomics. Nat. Methods 2017, 14 (5), 513–520. 10.1038/nmeth.4256. 28394336 PMC5409104

[ref35] Yu F.; Haynes S. E.; Nesvizhskii A. I. IonQuant Enables Accurate and Sensitive Label-Free Quantification With FDR-Controlled Match-Between-Runs. Mol. Cell. Proteomics 2021, 20, 100077 10.1016/j.mcpro.2021.100077. 33813065 PMC8131922

[ref36] da Veiga Leprevost F.; Haynes S. E.; Avtonomov D. M.; Chang H.-Y.; Shanmugam A. K.; Mellacheruvu D.; Kong A. T.; Nesvizhskii A. I. Philosopher: a versatile toolkit for shotgun proteomics data analysis. Nat. Methods 2020, 17 (9), 869–870. 10.1038/s41592-020-0912-y. 32669682 PMC7509848

