# Supplementary material for: Unexpected Noncovalent Off-Target Activity of Clinical BTK Inhibitors Leads to Discovery of a Dual NUDT5/14 Antagonist
Source: J Med Chem. 2024 Apr 18;67(9):7245–59. doi: 10.1021/acs.jmedchem.4c00072 (PMC11089510; doi:10.1021/acs.jmedchem.4c00072)
Supplement: Supplementary file 1 — jm4c00072_si_001.pdf [file jm4c00072_si_001.pdf]

Supporting Information

Unexpected non-covalent off-target activity of clinical BTK inhibitors leads to discovery of a dual NUDT5/14 antagonist

Esra Balıkçı[1,2†], Anne-Sophie M. C. Marques[1,2†], Ludwig G. Bauer[1,2†]†, Raina Seupel[1,2], James Bennett[1,2], Brigitt Raux[1,2], Karly Buchan[1,2], Klemensas Simelis[1,2], Usha Singh[1,2], Catherine Rogers[1,2], Jennifer Ward [1,2], Carol Cheng[1,2], Tamas Szommer[1,2], Kira Schuetzenhofer[5], Jonathan M. Elkins[1,2], David L. Sloman[5], Ivan Ahel[4], Oleg Fedorov[1,2], Paul E. Brennan[1,2,3], Kilian V. M. Huber[1,2*]

[1]Centre for Medicines Discovery, Nuffield Department of Medicine, University of Oxford, Old Road Campus, Roosevelt Drive, Oxford OX3 7FZ, UK

[2]Target Discovery Institute, Nuffield Department of Medicine, University of Oxford, Old Road Campus, Roosevelt Drive, Oxford OX3 7FZ, UK

[3]Alzheimer's Research UK Oxford Drug Discovery Institute, Nuffield Department of Medicine, University of Oxford, Old Road Campus, Roosevelt Drive, Oxford OX3 7FZ, UK

[4]Sir William Dunn School of Pathology, University of Oxford, South Parks Road, Oxford OX1 3RE, UK

[5]Departments of Discovery Chemistry, Merck & Co. Inc., 33 Avenue Louis Pasteur, Boston, Massachusetts 02115, United States

[†]These authors contributed equally to this work

[*]Correspondence:

kilian.huber@cmd.ox.ac.uk

Table of Contents

Supplementary Figures......S3

Figure S1. Chemical proteomics results......S3

Figure S2. NUDT5 NanoBRET TE assay......S4

Figure S3. Mass spectrometry analysis of ibrutinib analogues with NUDT5......S5-7

Figure S4. Mass spectrometry analysis of ibrutinib analogues with NUDT14......S8-10

Figure S5. Multiple sequence alignment of NUDT14 and NUDT5......S11

Table S1. NUDIX selectivity for compound **9** and ibrutinib (**1**) determined by SPR......S11

Figure S6. Setup of HiBiT-NUDT14 CETSA......S12

Figure S7. Assessment of protein ADP-ribosylation......S12

NMR spectra and LC-MS chromatograms......S13-43

Table S2. X-ray data collection and refinement statistics......S44

Table S3. Primers for cloning......S45

Supplementary Figures

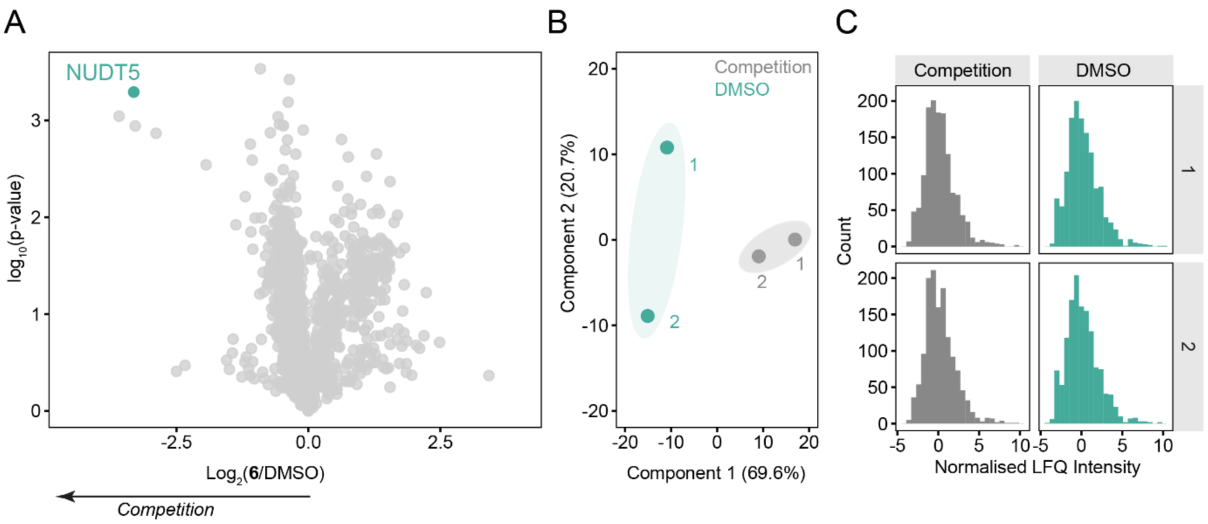

Figure S1. Evaluation of **6** by compound centric chemical proteomics using CBH-003 (**7**) affinity matrix enables enrichment of endogenous NUDT5 from cellular lysates. Target proteins are pulled down from lysates and analysed by LC-MS/MS. (A) Chemoproteomic result shows NUDT5 as significantly enriched and competed by 20 µM of **6** (n = 2). (B) Principal component analysis and (C) histograms of measured pulldown samples show clustering of DMSO or competition conditions and uniform distribution of quantified proteins respectively confirming method robustness.

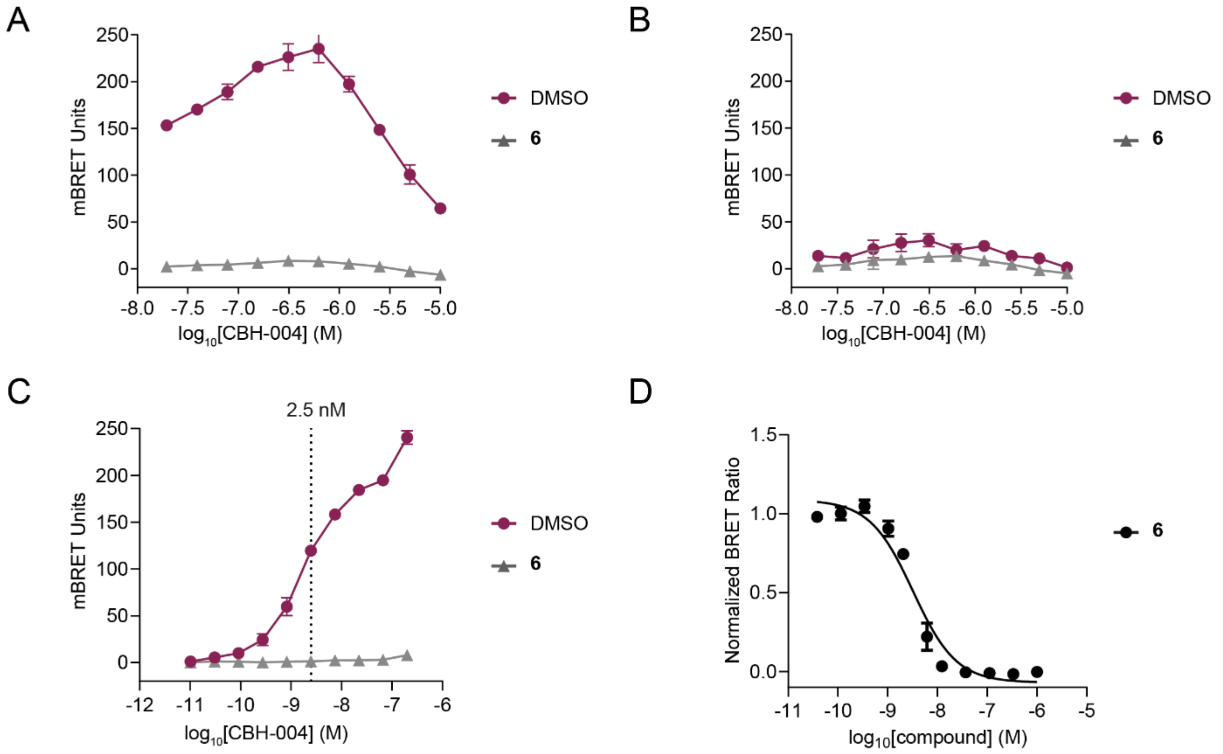

Figure S2. NUDT5 NanoBRET TE assay setup with CBH-004 (**8**) and overexpressed NanoLuc-NUDT5 in HEK293 cells. (A) Titration of **8** +/- 10 µM of **6** in HEK cells with overexpressed N-terminal NanoLuc-NUDT5 or (B) C-terminal NanoLuc-NUDT5. (C) Final compound **8** titration with N-terminal NanoLuc-NUDT5 to determine tracer concentration for optimal assay window. (D) Dose-dependent competition with **6** using 2.5 nM of **8**. Data are shown as mean ± SD and are based on three technical replicates. Graph is representative of two independent biological replicates (n = 2) (EC_{50} = 0.004 ± 0.001 µM).

Compound 1

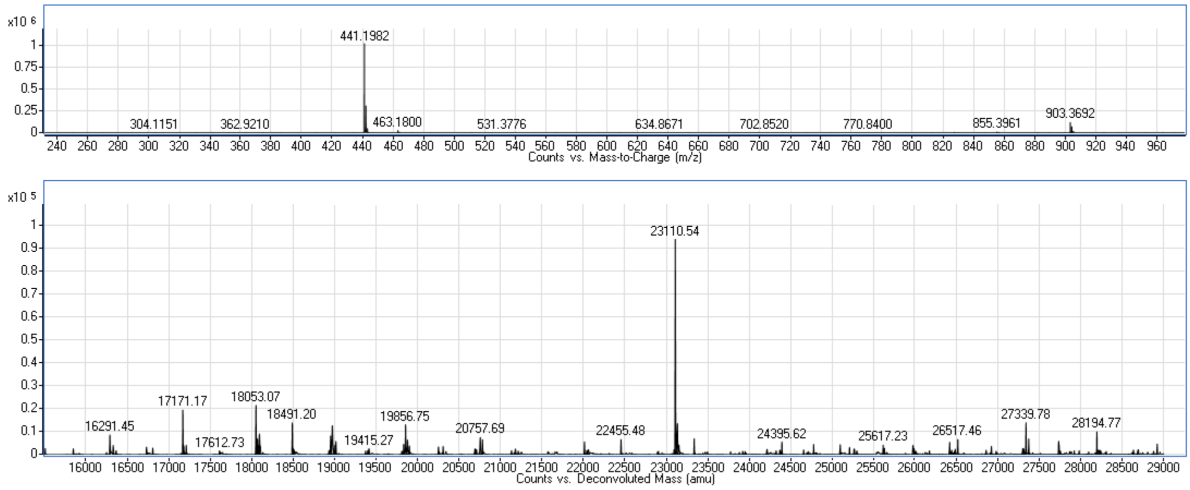

Compound 9

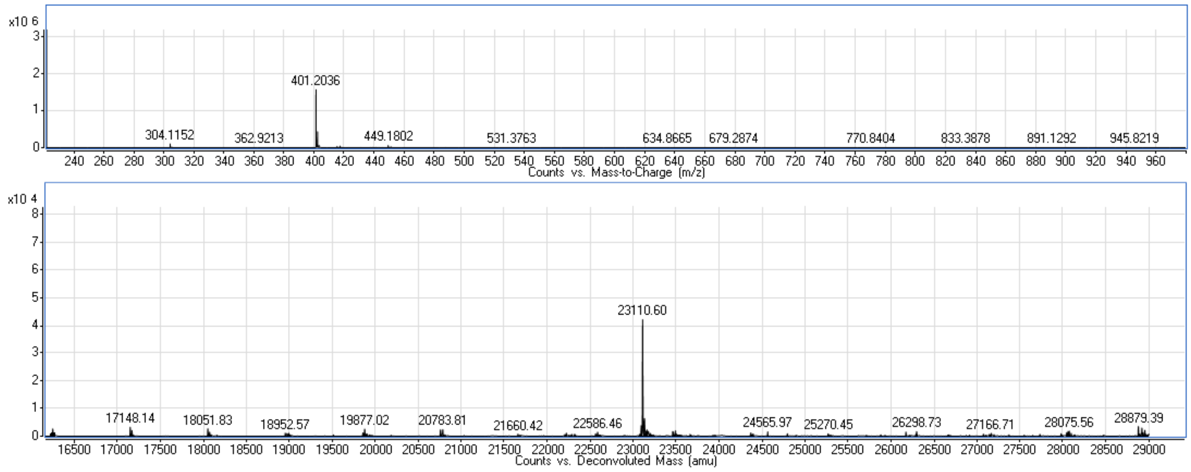

Compound 10

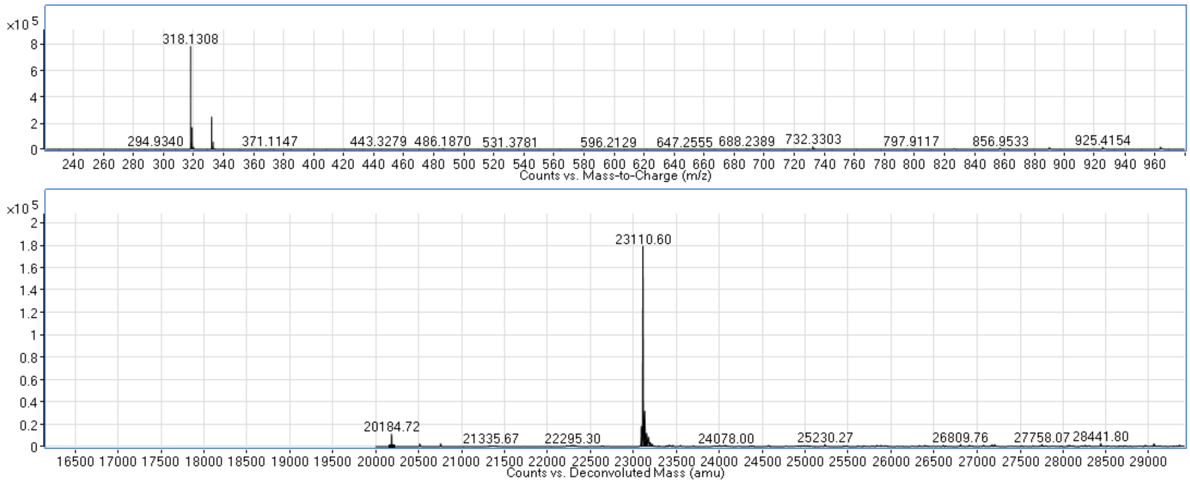

Compound 11

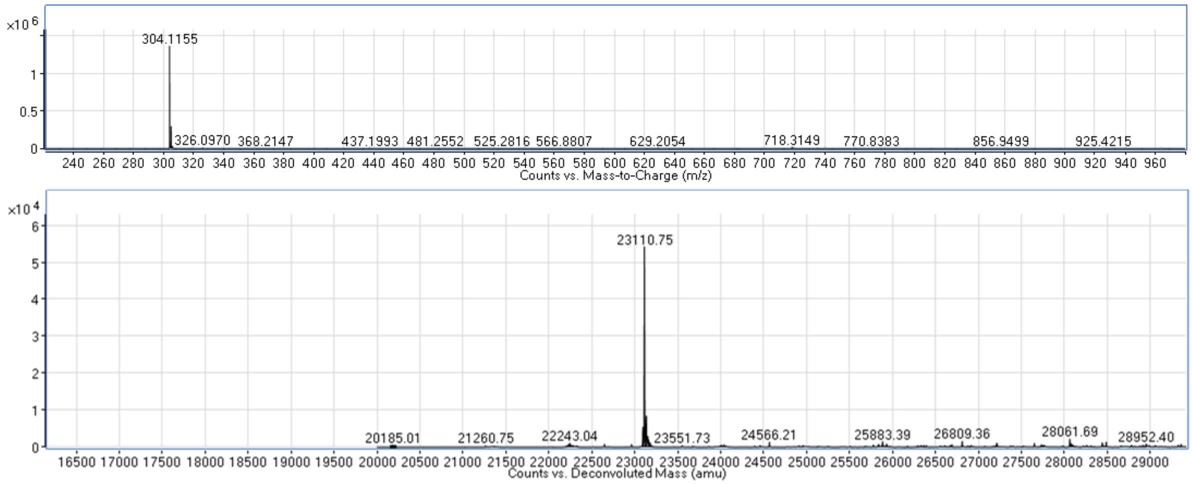

Compound 12

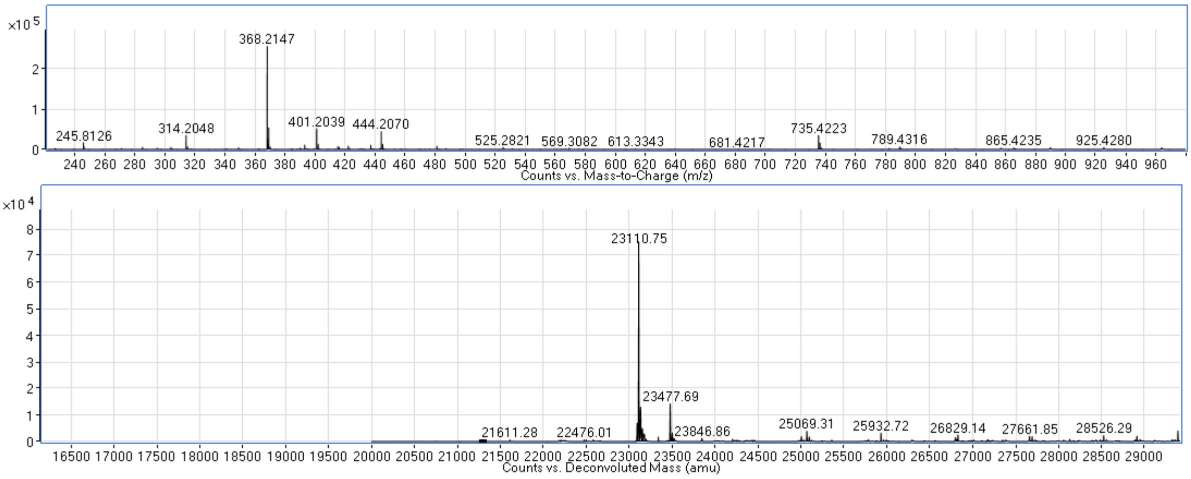

Compound 13

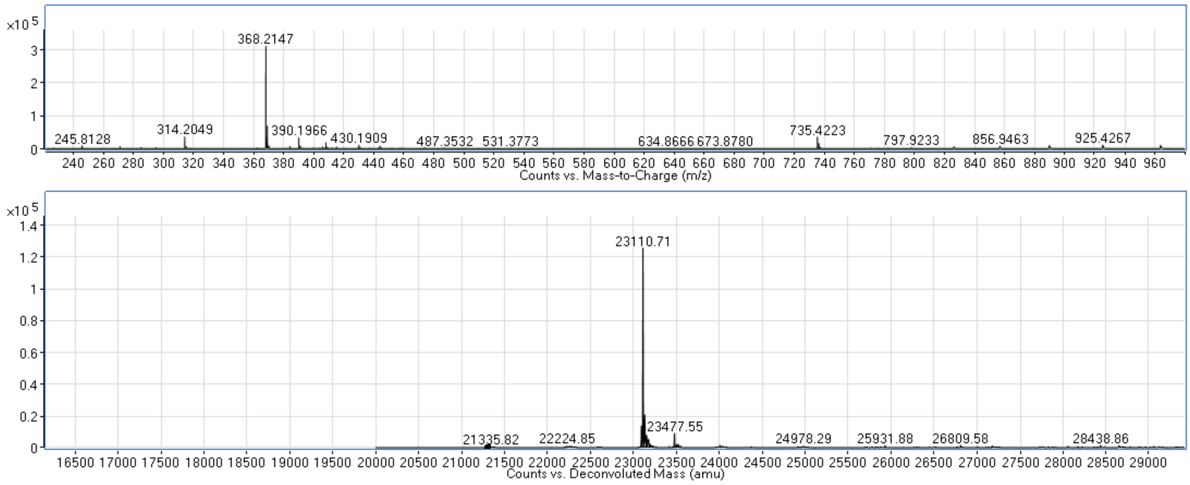

Compound 14

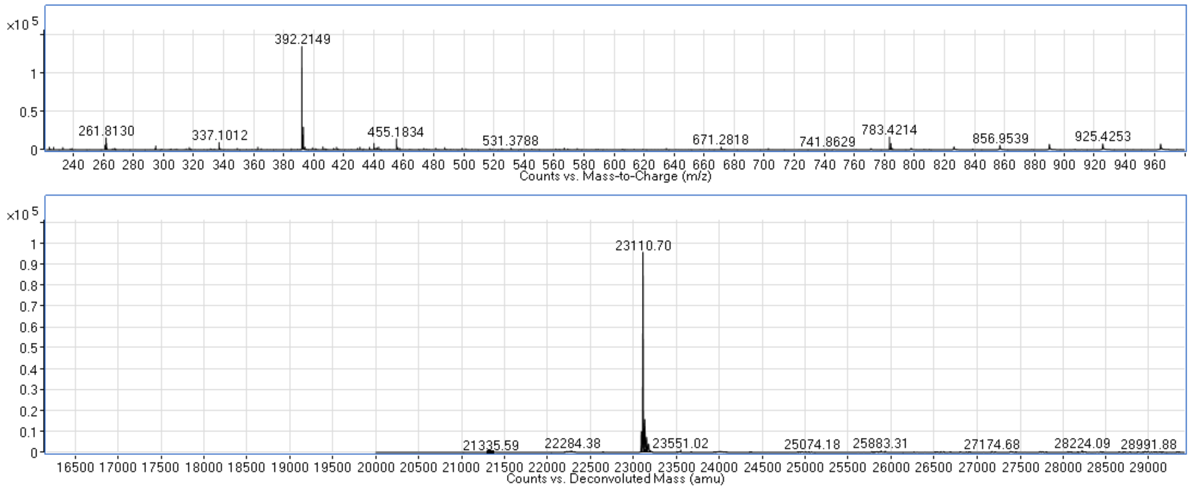

Compound 15

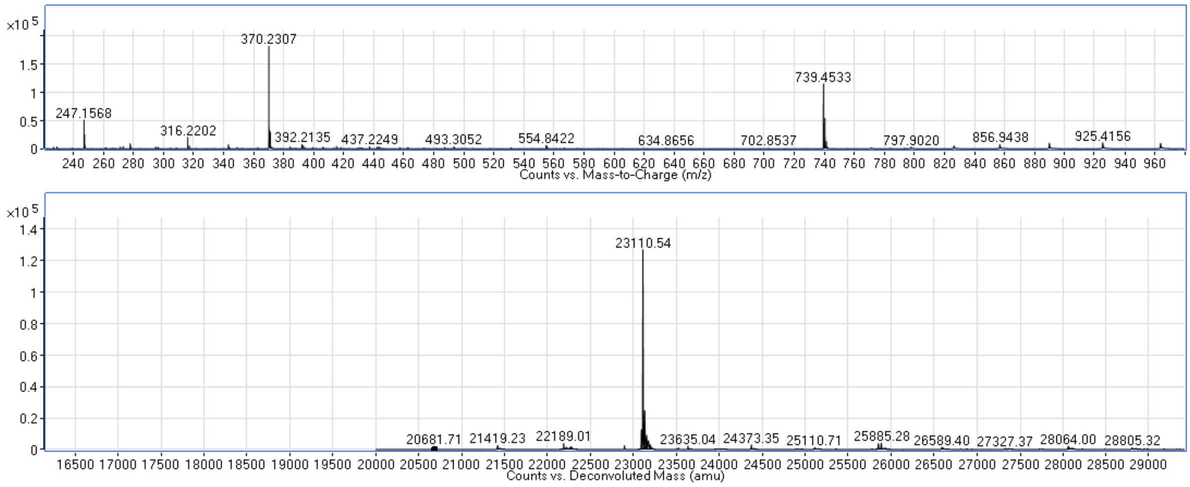

Figure S3. Intact protein mass spectra of NUDT5 with ibrutinib (**1**) and ibrutinib analogues (**9**-**15**) after 2 h incubation. (The corresponding compounds mass spectra are included for each molecule, NUDT5 molecular weight corresponds to 23110 Da).

Compound 1

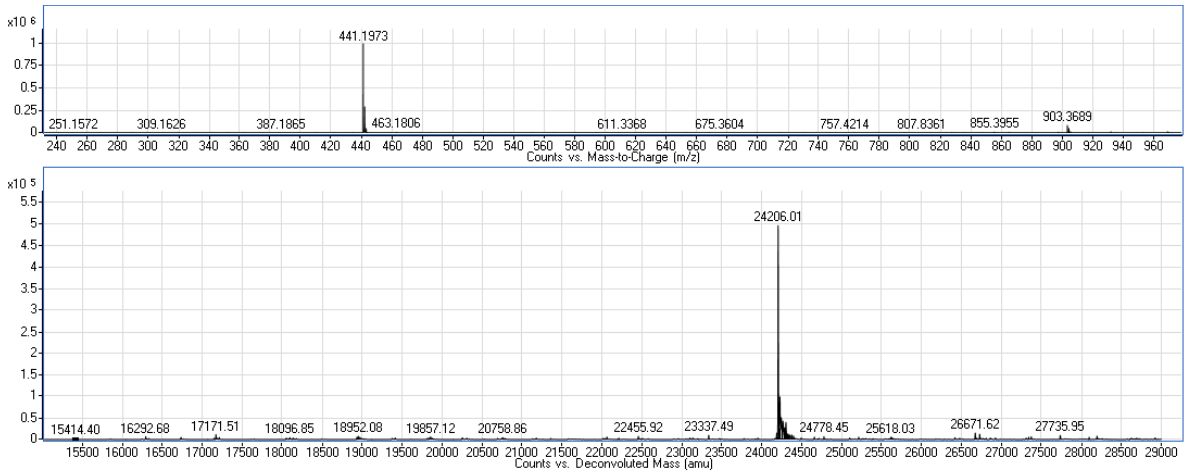

Compound 9

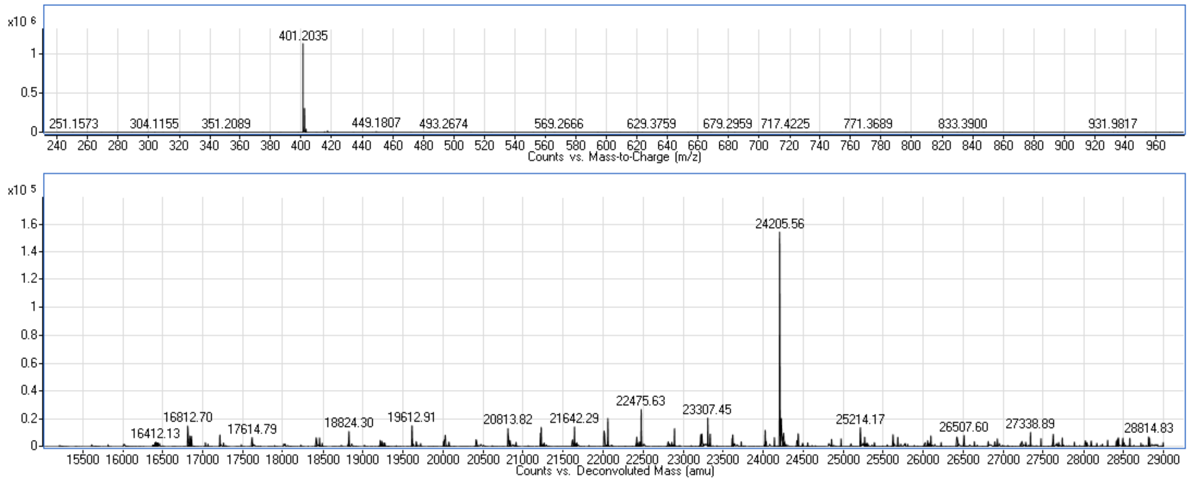

Compound 10

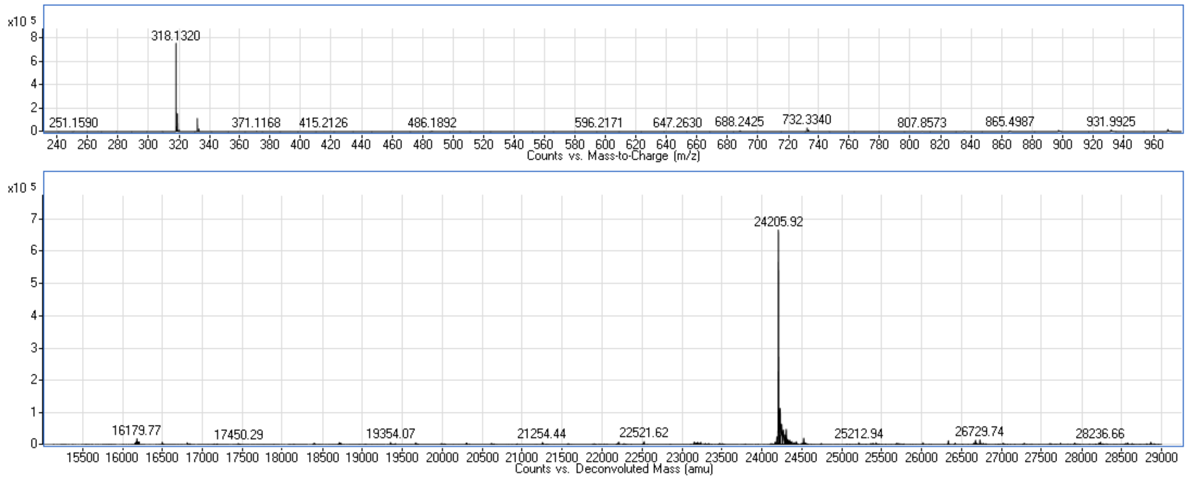

Compound 11

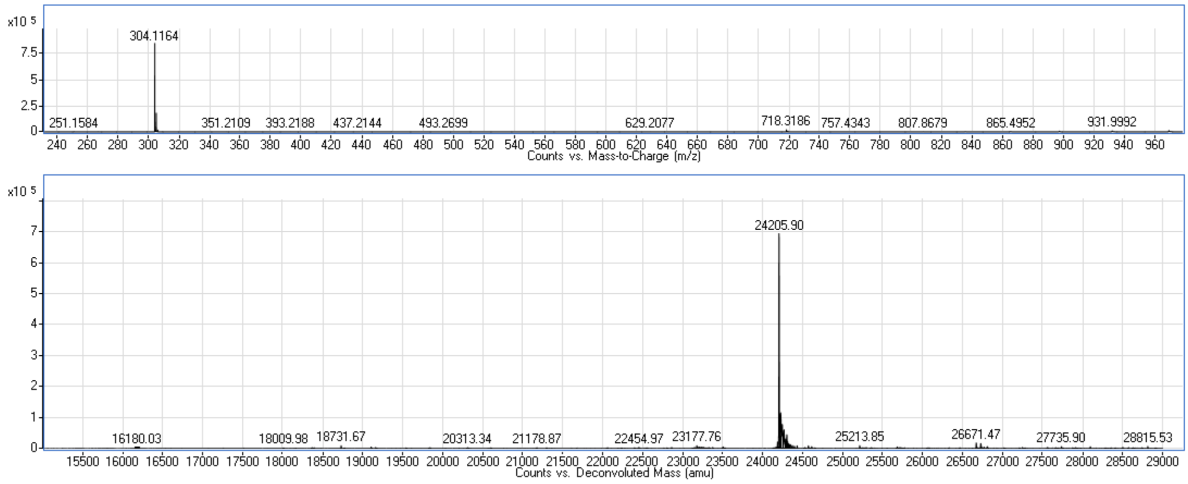

Compound 12

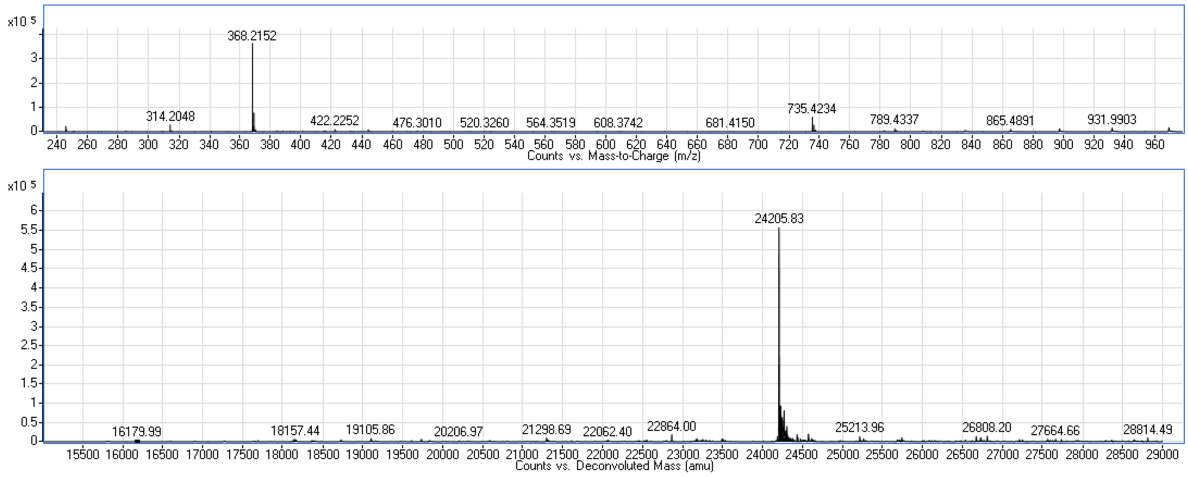

Compound 13

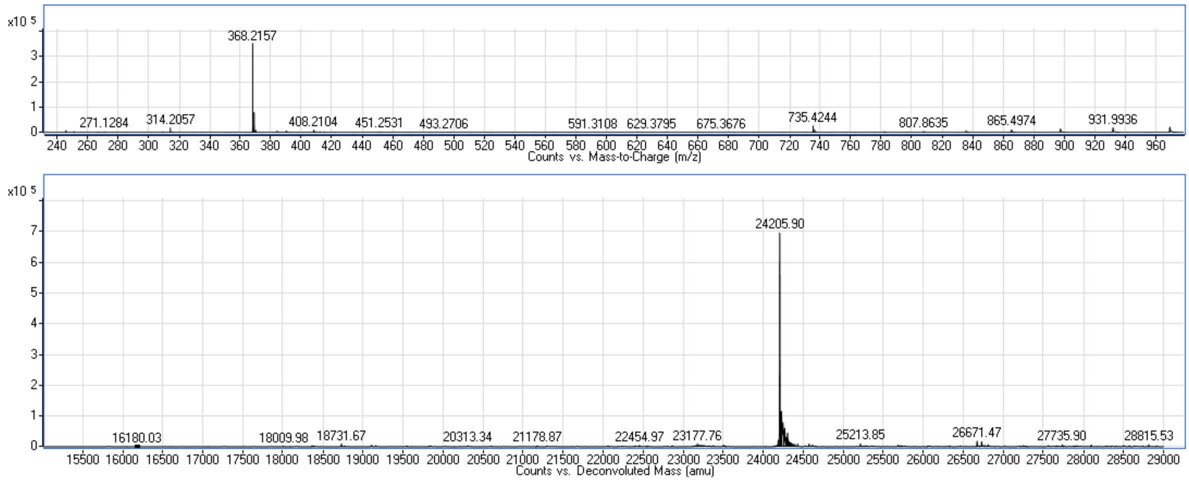

Compound 14

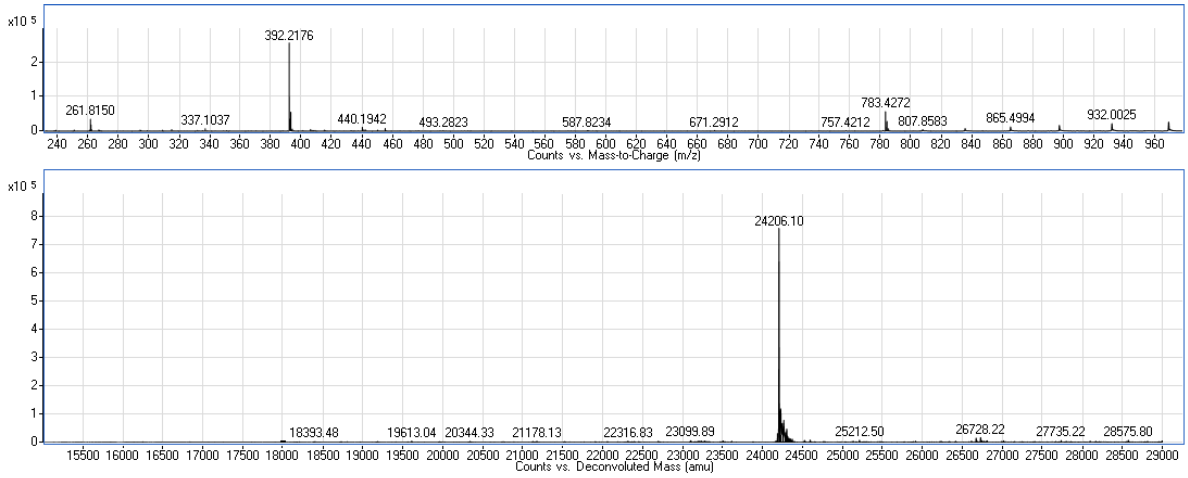

Compound 15

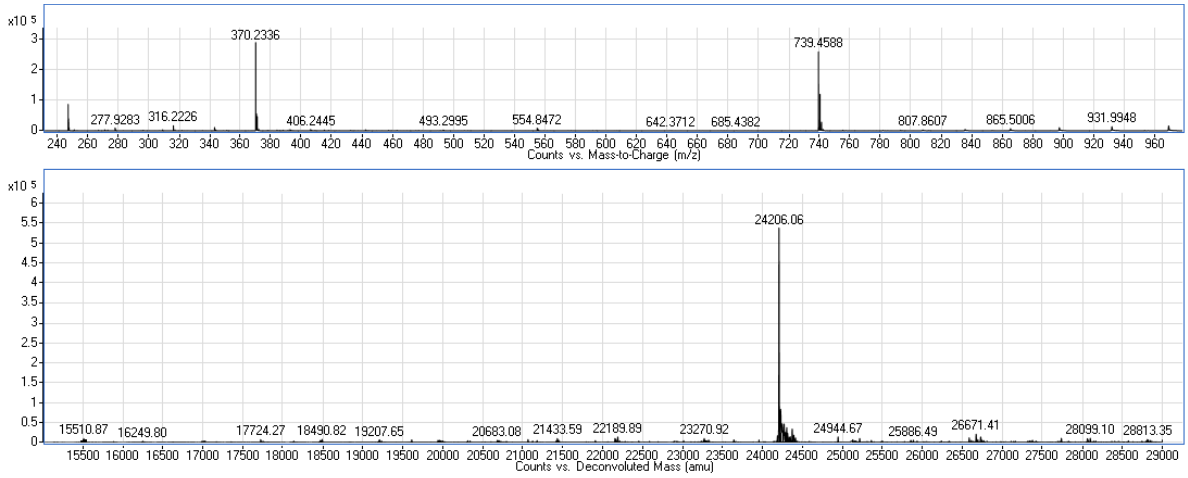

Figure S4. Intact protein mass spectra of NUDT14 with ibrutinib (**1**) and ibrutinib analogues (**9**-**15**) after 2 h incubation. (The corresponding compounds mass spectra are included for each molecule, NUDT14 molecular weight corresponds to 24206 Da).

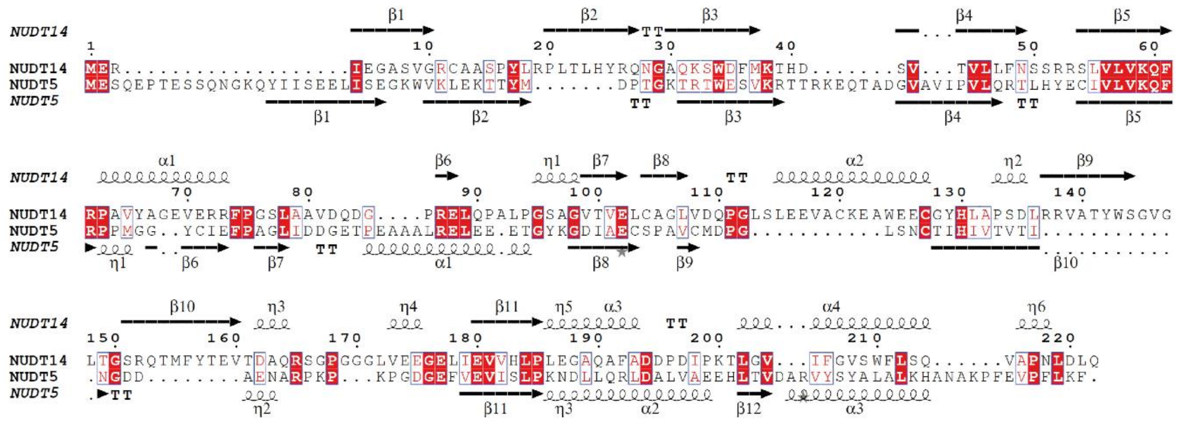

Figure S5. Multiple sequence alignment of NUDT14 and NUDT5 (Clustal Omega). Identical sequences are highlighted in red while similar residues are in red letters with blue squares.

Table S1: NUDIX selectivity for compound **9** and ibrutinib (**1**) determined by SPR. NB, no binding (K_D > 100 μM). Data are shown as mean ± SD and are based on two independent biological replicates (n = 2).

	compound K_D (μM), (n=2)	
NUDIX protein	1	9
NUDT14	1.90 ± 0.50	0.368 ± 0.020
NUDT5	0.209 ± 0.062	0.237 ± 0.081
NUDT1	3.50 ± 0.28	NB
NUDT15	NB	NB
NUDT7	NB	NB
NUDT18	NB	NB
NUDT12	NB	NB
NUDT9	NB	NB
NUDT21	NB	NB
NUDT17	NB	NB
NUDT16	NB	NB
NUDT16L1	NB	NB

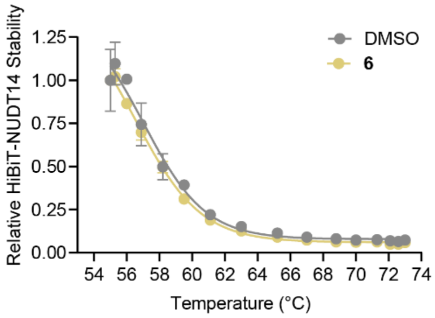

Figure S6. HiBiT-NUDT14 CETSA. The specific NUDT5 inhibitor **6** does not stabilise NUDT14 compared to DMSO. Graph is representative of two independent experiments (n = 2).

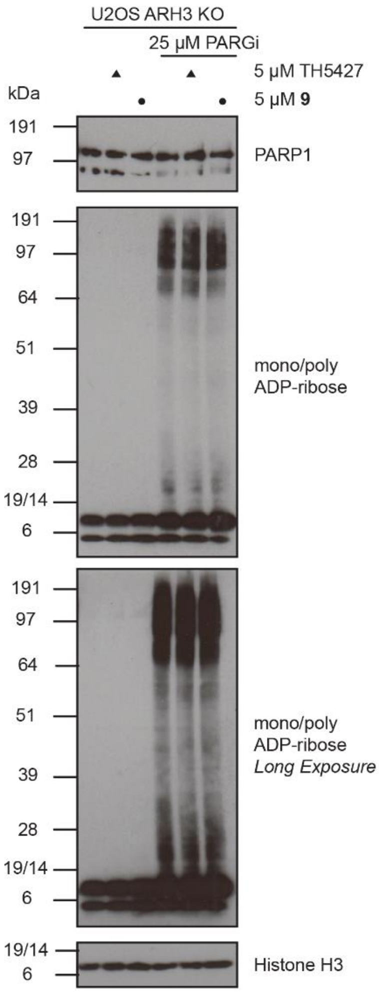

Figure S7. Assessment of protein ADP-ribosylation. Treatment of human cells with the inhibitors does not lead to changes in protein ADP-ribosylation. U2OS ARH3 KO cells were treated with the indicated inhibitors or DMSO for 3 days. ADPr levels were analysed using Western blotting.

NMR Spectra and LC-MS chromatograms

3-bromo-1-(1-methylpiperidin-4-yl)-1*H*-pyrazolo[3,4-*d*]pyrimidin-4-amine (17a)

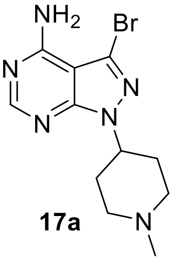

17a

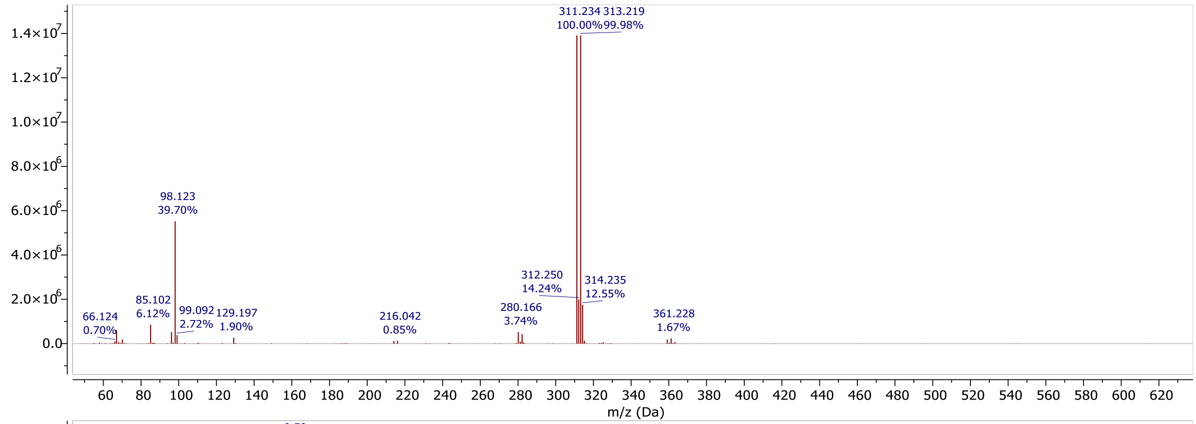

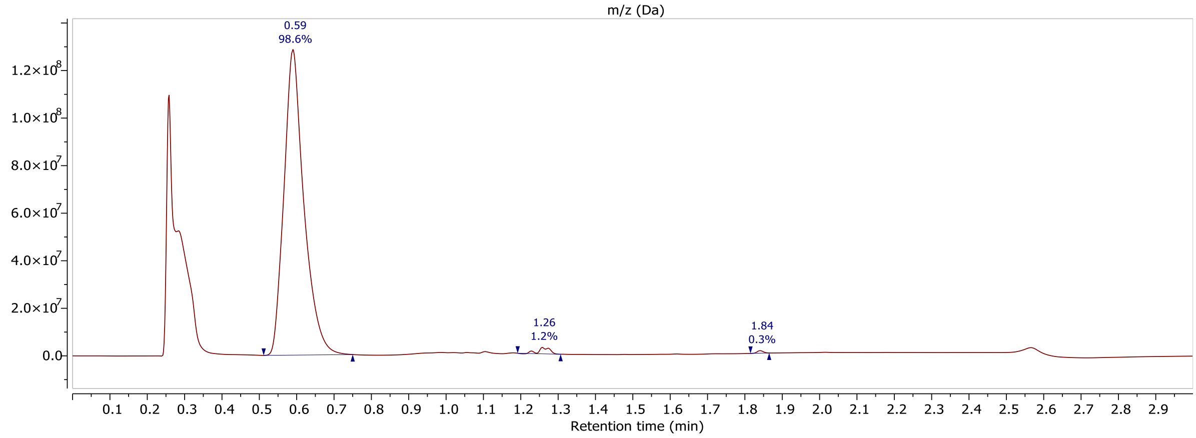

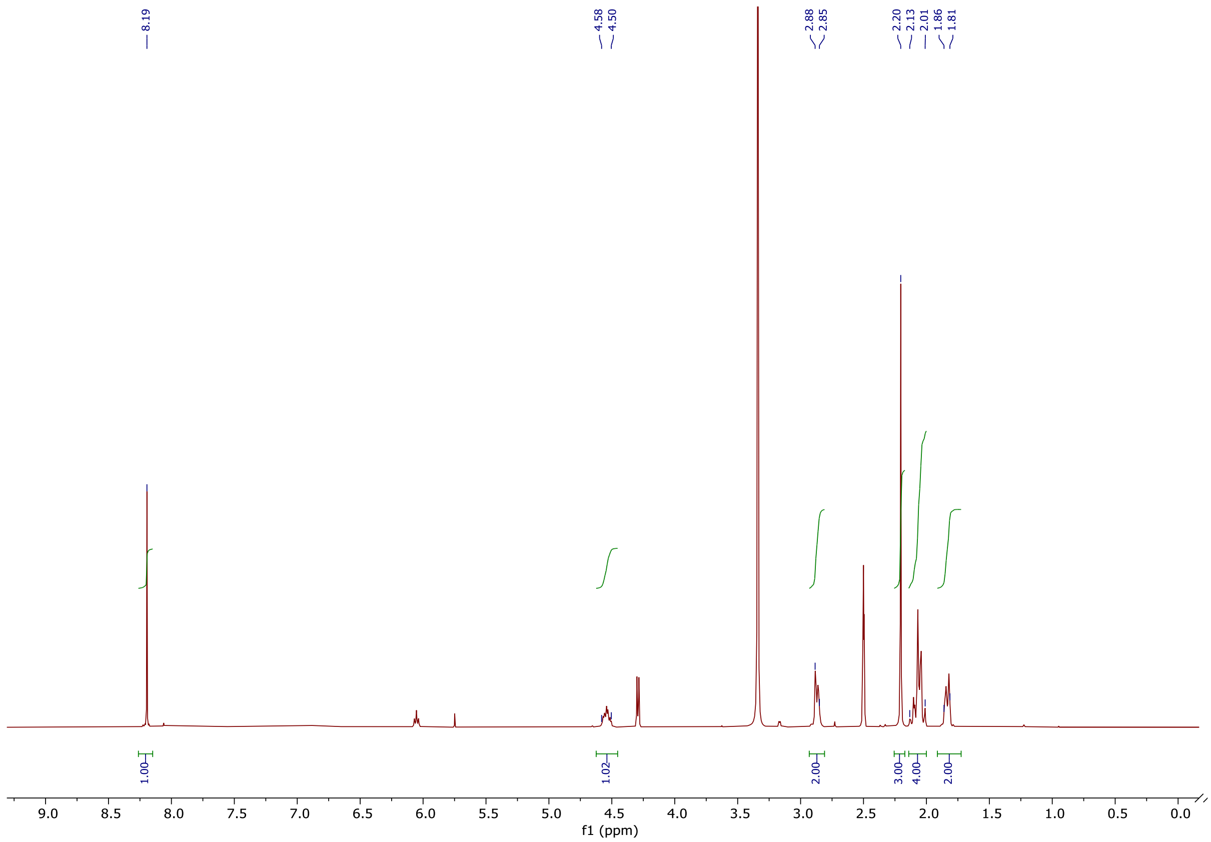
8.19
4.58
4.50
2.88
2.85
2.20
2.13
2.01
1.86
1.81
1.00
1.02
2.00
3.00
4.00
2.00
9.0 8.5 8.0 7.5 7.0 6.5 6.0 5.5 5.0 4.5 4.0 3.5 3.0 2.5 2.0 1.5 1.0 0.5 0.0
f1 (ppm)

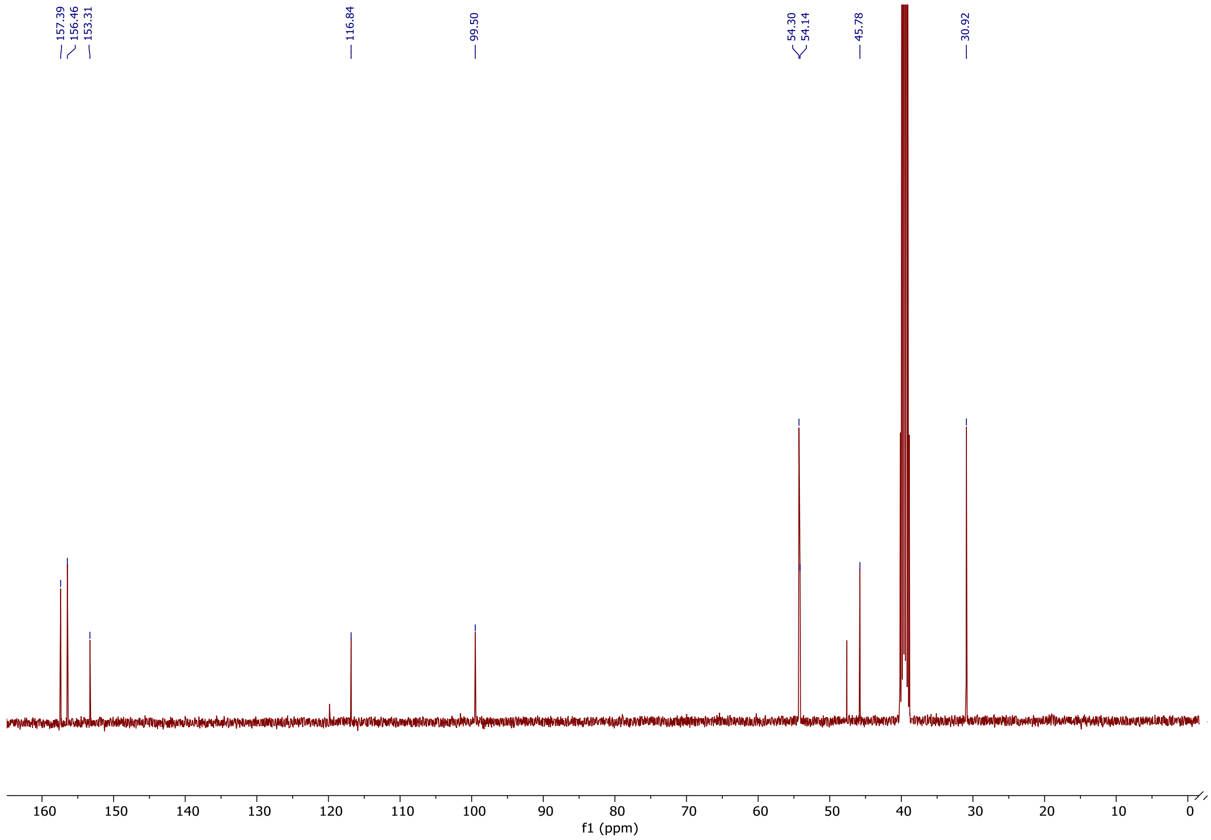
157.39
156.46
153.31
116.84
99.50
54.30
54.14
45.78
30.92
160 150 140 130 120 110 100 90 80 70 60 50 40 30 20 10 0
f1 (ppm)

1-(1-methylpiperidin-4-yl)-3-(4-phenoxyphenyl)-1*H*-pyrazolo[3,4-*d*]pyrimidin-4-amine (9)

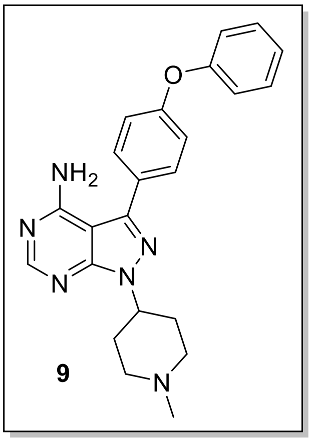

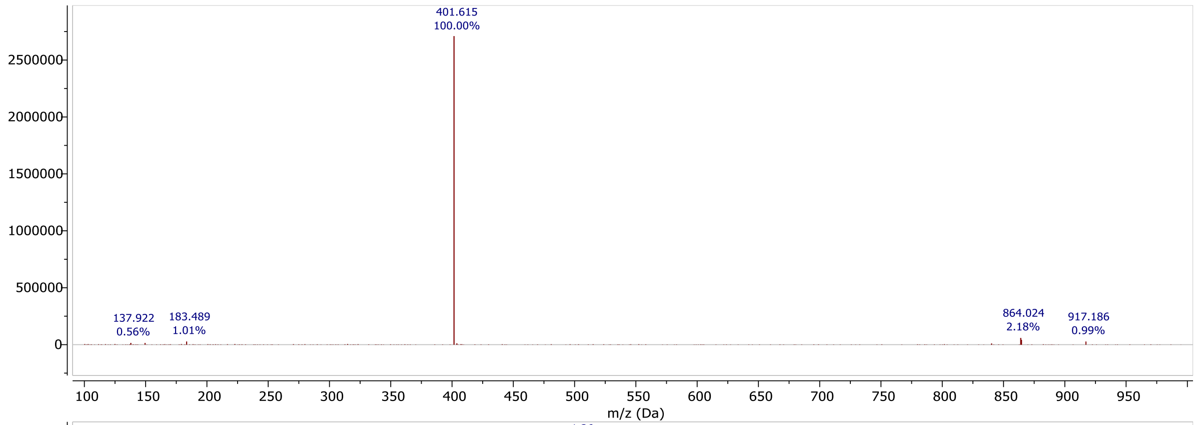

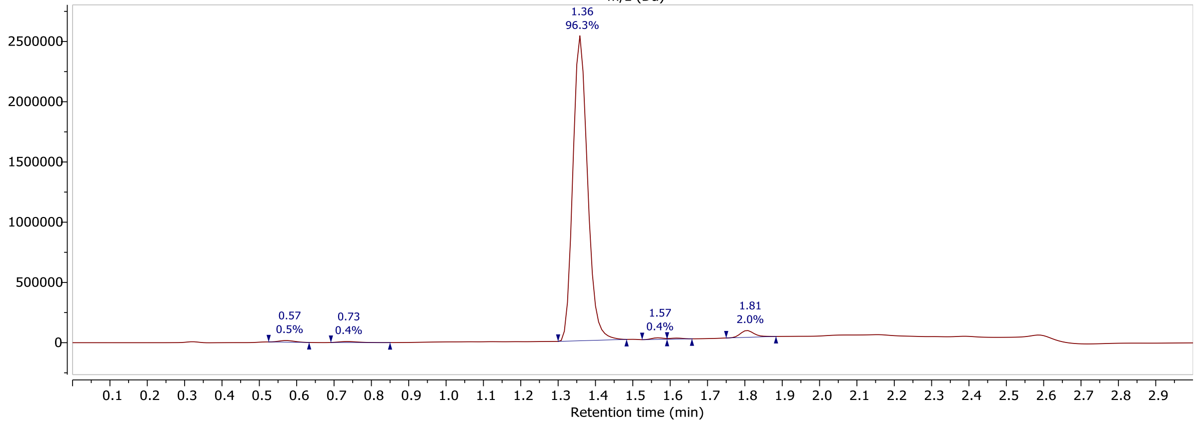

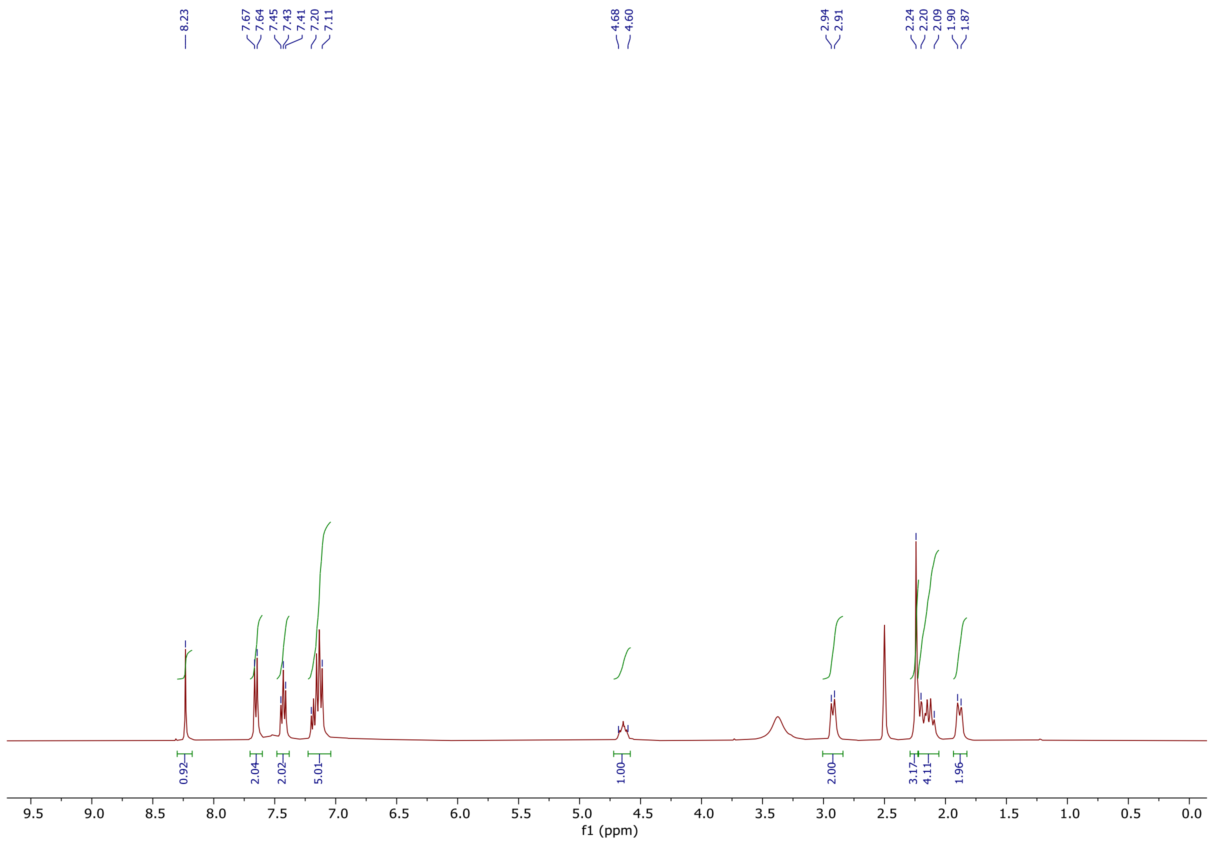
8.23
7.67
7.64
7.45
7.43
7.41
7.20
7.11
4.68
4.60
2.94
2.91
2.24
2.20
2.09
1.90
1.87
0.92
2.04
2.02
5.01
1.00
2.00
3.17
4.11
1.96
f1 (ppm)

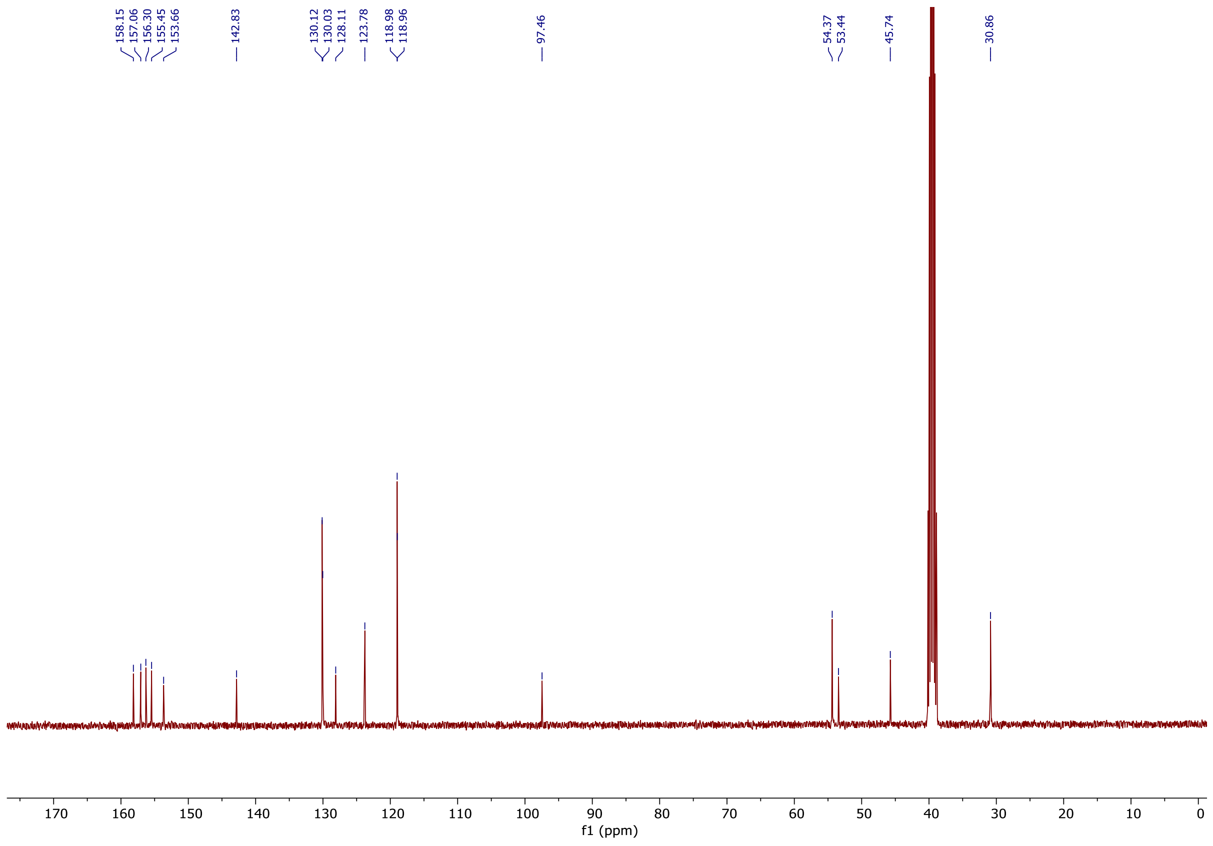
158.15
157.06
156.30
155.45
153.66
142.83
130.12
130.03
128.11
123.78
118.98
118.96
97.46
54.37
53.44
45.74
30.86
f1 (ppm)

1-methyl-3-(4-phenoxyphenyl)-1*H*-pyrazolo[3,4-*d*]pyrimidin-4-amine (10)

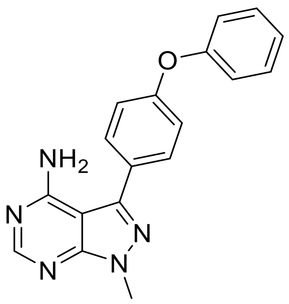

10

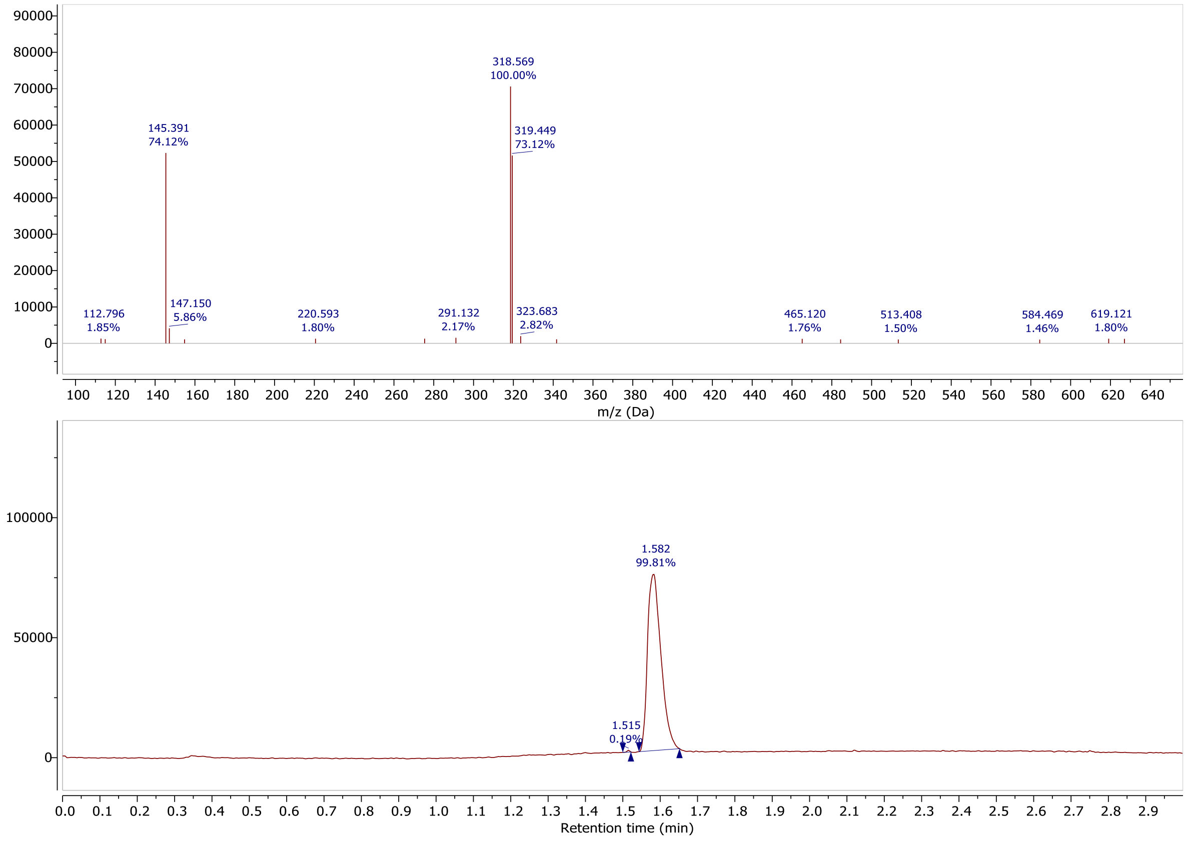

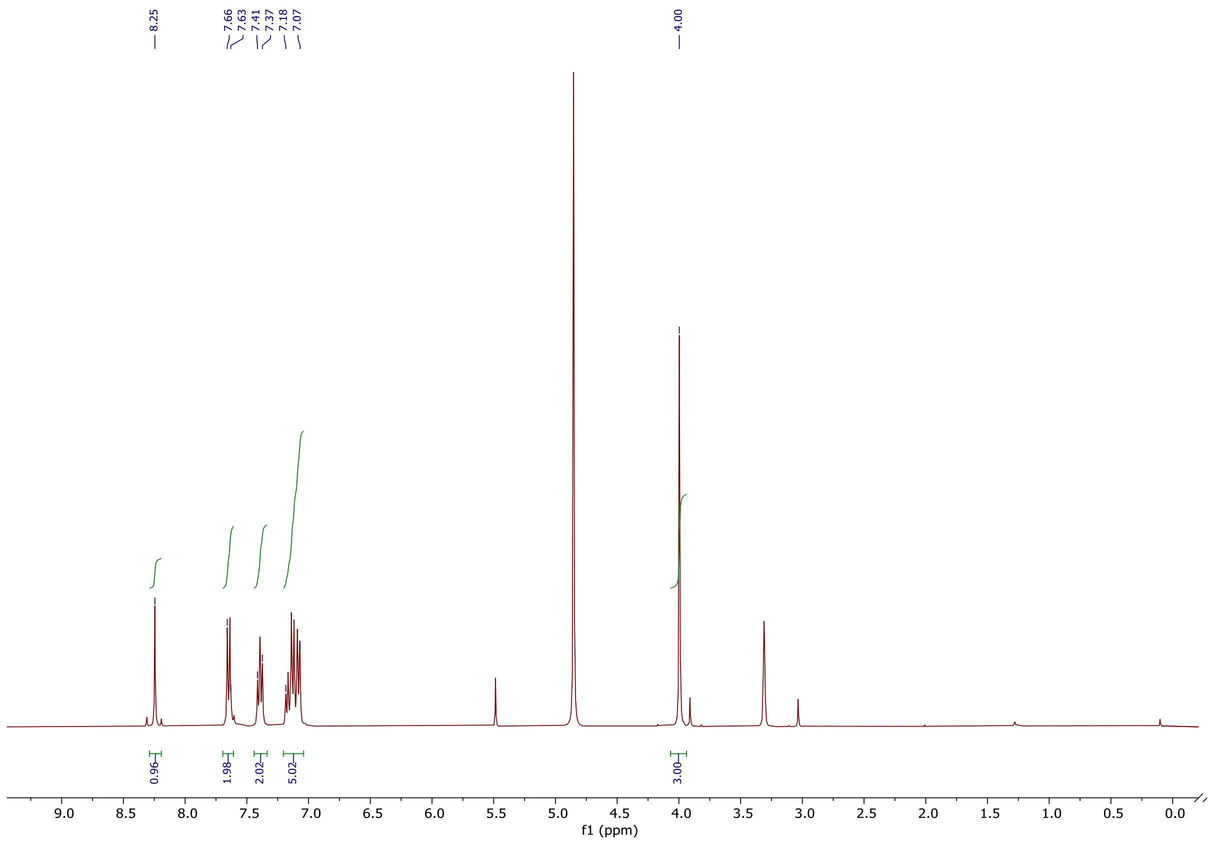
8.25
7.66
7.63
7.41
7.37
7.18
7.07
4.00
0.96
1.98
2.02
5.02
3.00
f1 (ppm)

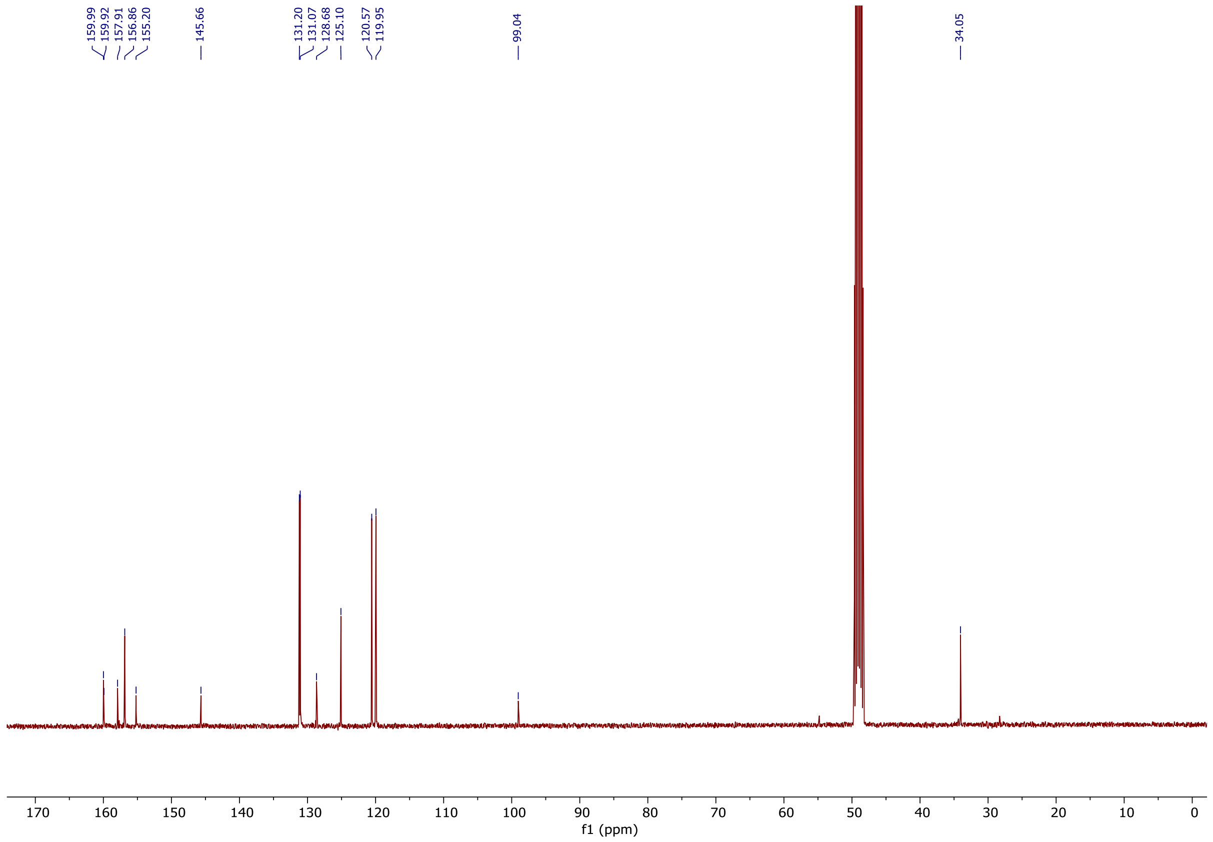
159.99
159.92
157.91
156.86
155.20
145.66
131.20
131.07
128.68
125.10
120.57
119.95
99.04
34.05
f1 (ppm)

3-(4-phenoxyphenyl)-1*H*-pyrazolo[3,4-*d*]pyrimidin-4-amine (11)

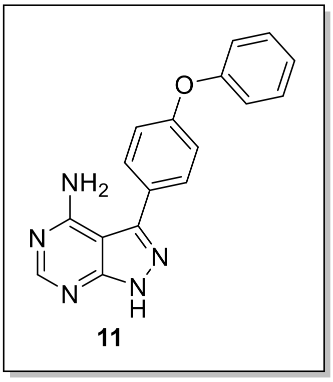

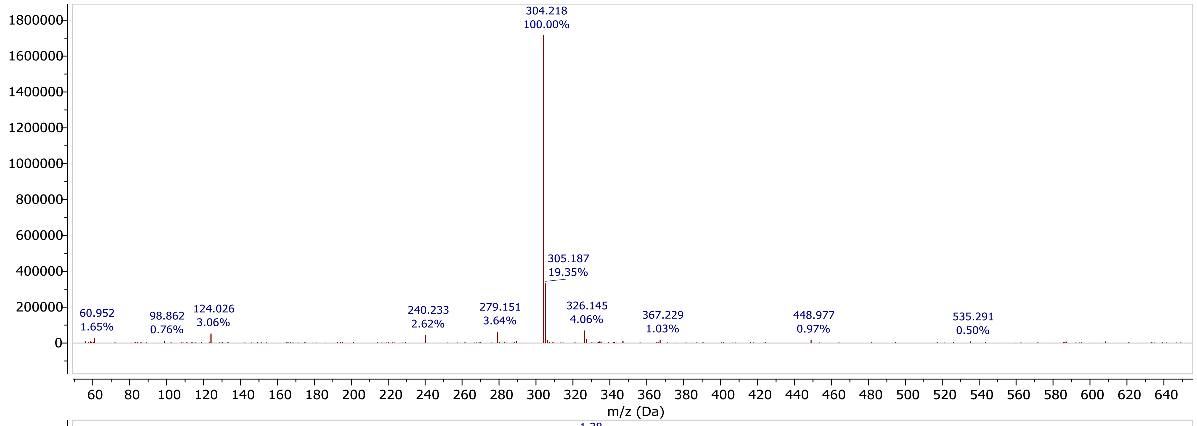

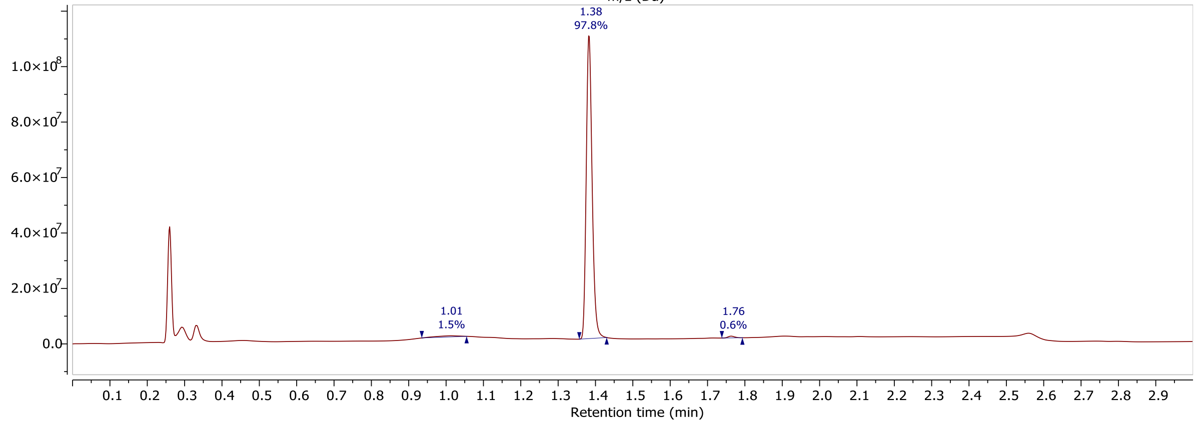

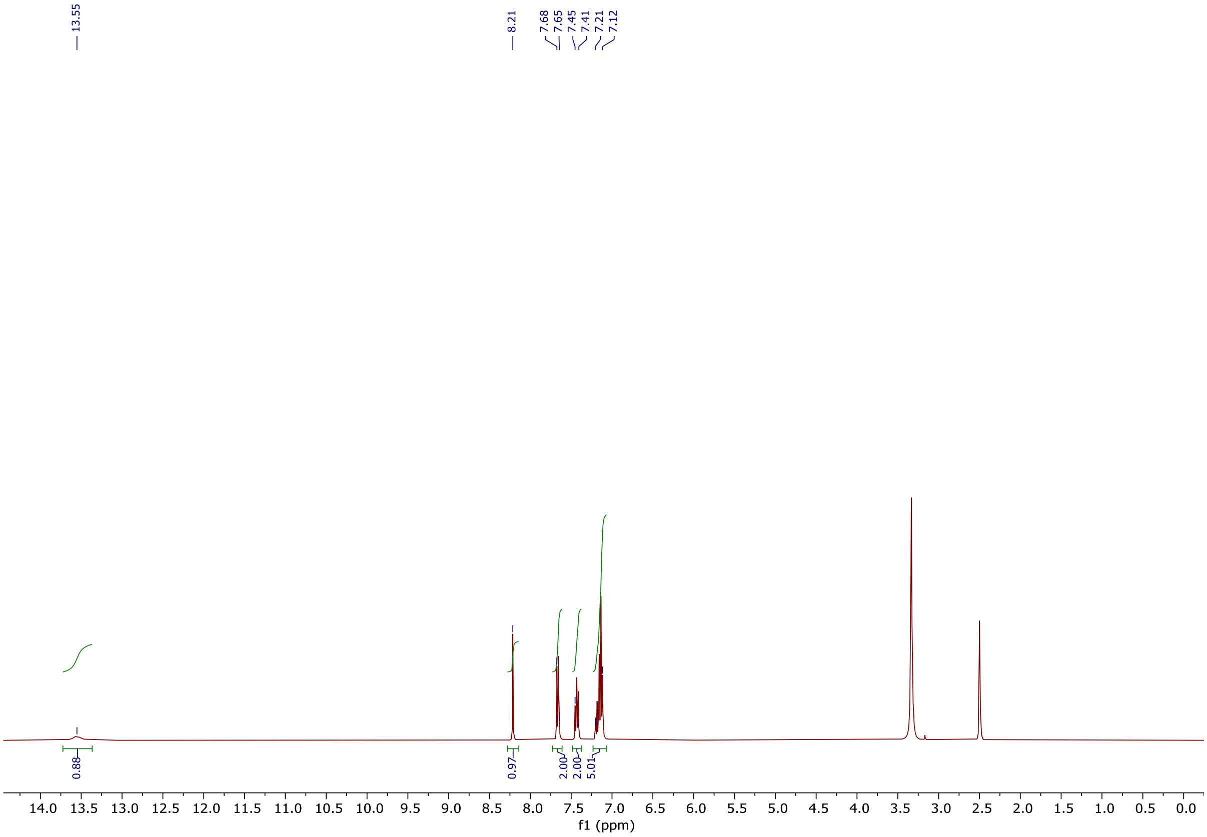
13.55
8.21
7.68
7.65
7.45
7.41
7.21
7.12
0.88
0.97
2.00
2.00
5.01
f1 (ppm)

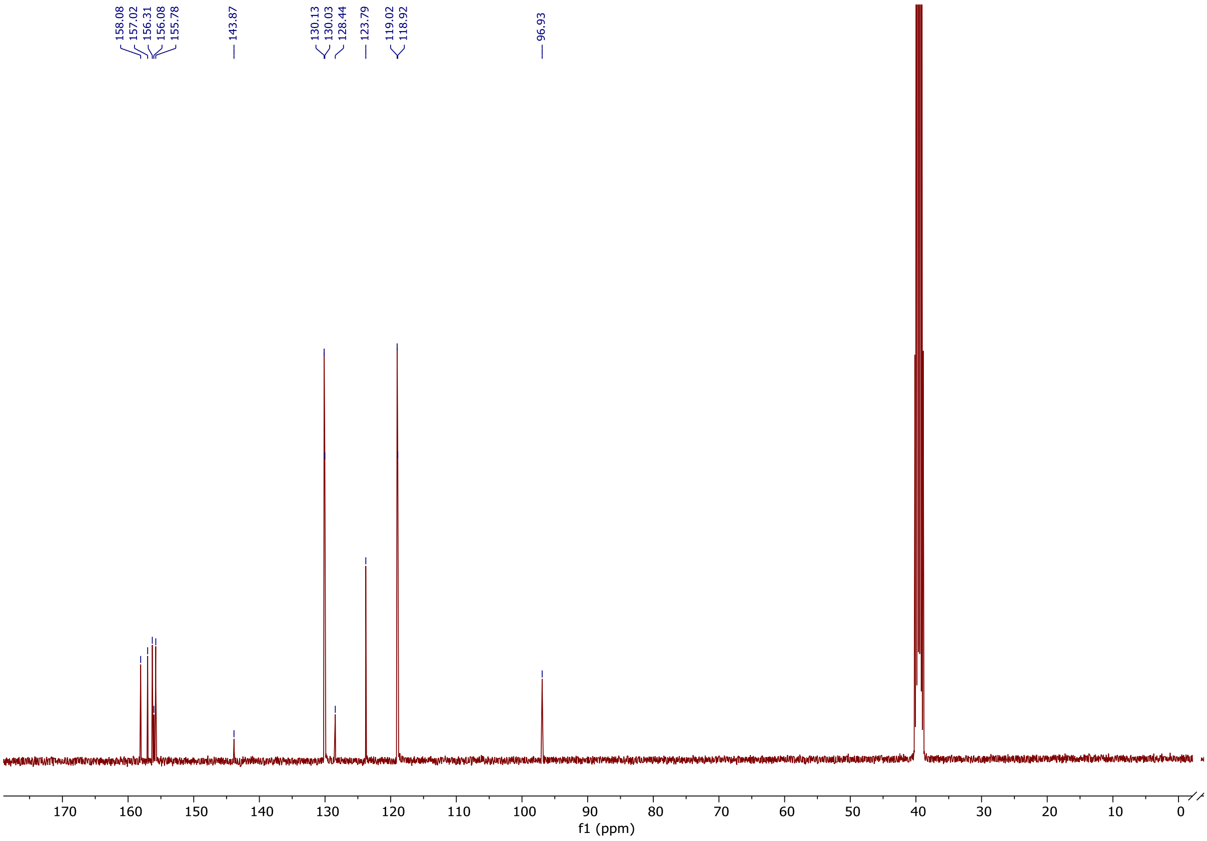
158.08
157.02
156.31
156.08
155.78
143.87
130.13
130.03
128.44
123.79
119.02
118.92
96.93
f1 (ppm)

***Tert*-butyl 4-(4-amino-1-(1-methylpiperidin-4-yl)-1*H*-pyrazolo[3,4-*d*]pyrimidin-3-yl)-3,6-dihydropyridine-1(2*H*)-carboxylate (18a)**

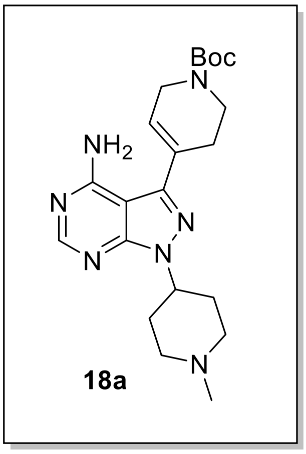

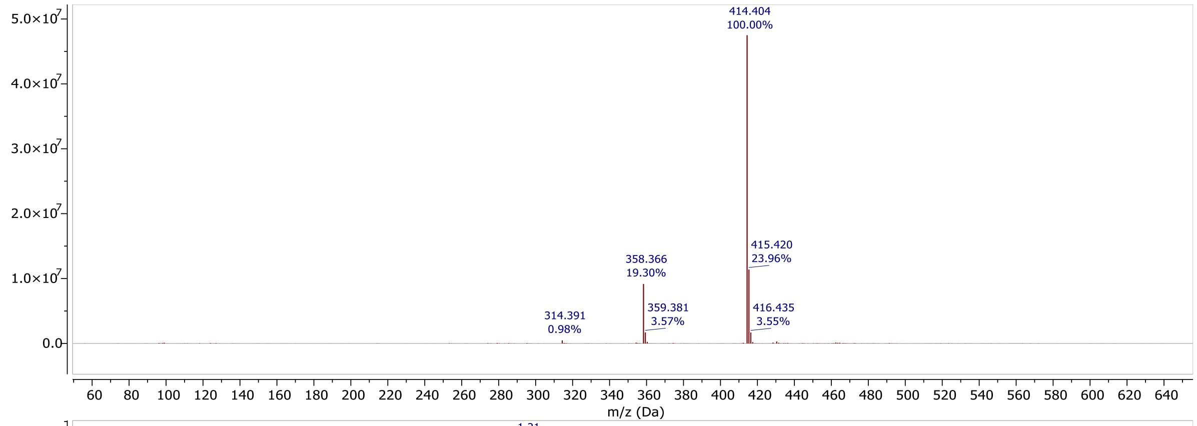

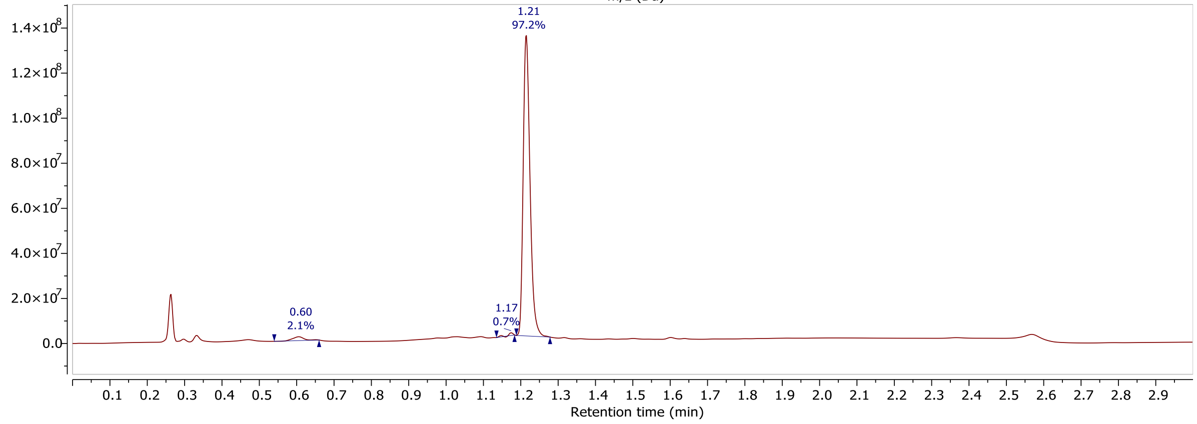

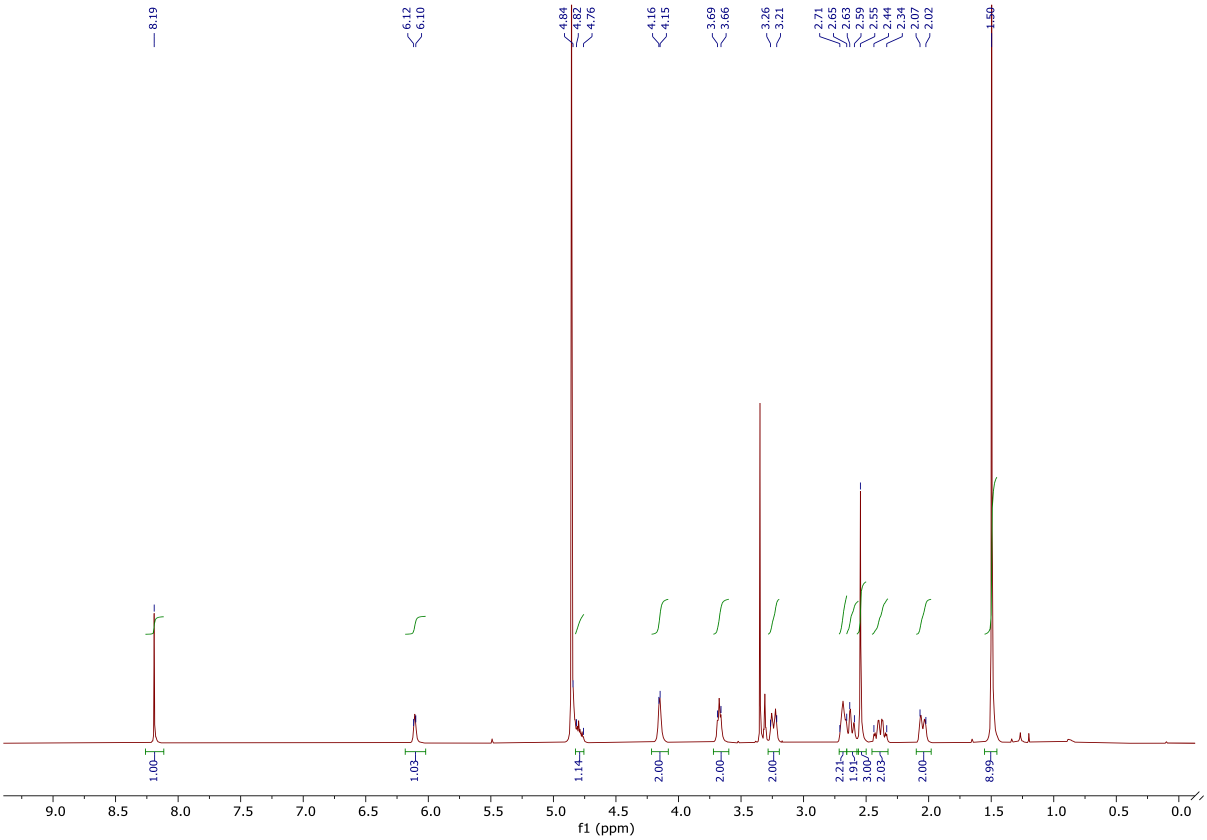
8.19
6.12
6.10
4.84
4.82
4.76
4.16
4.15
3.69
3.66
3.26
3.21
2.71
2.65
2.63
2.59
2.55
2.44
2.34
2.07
2.02
1.50
1.00
1.03
1.14
2.00
2.00
2.00
2.21
1.91
3.00
2.03
2.00
8.99
f1 (ppm)

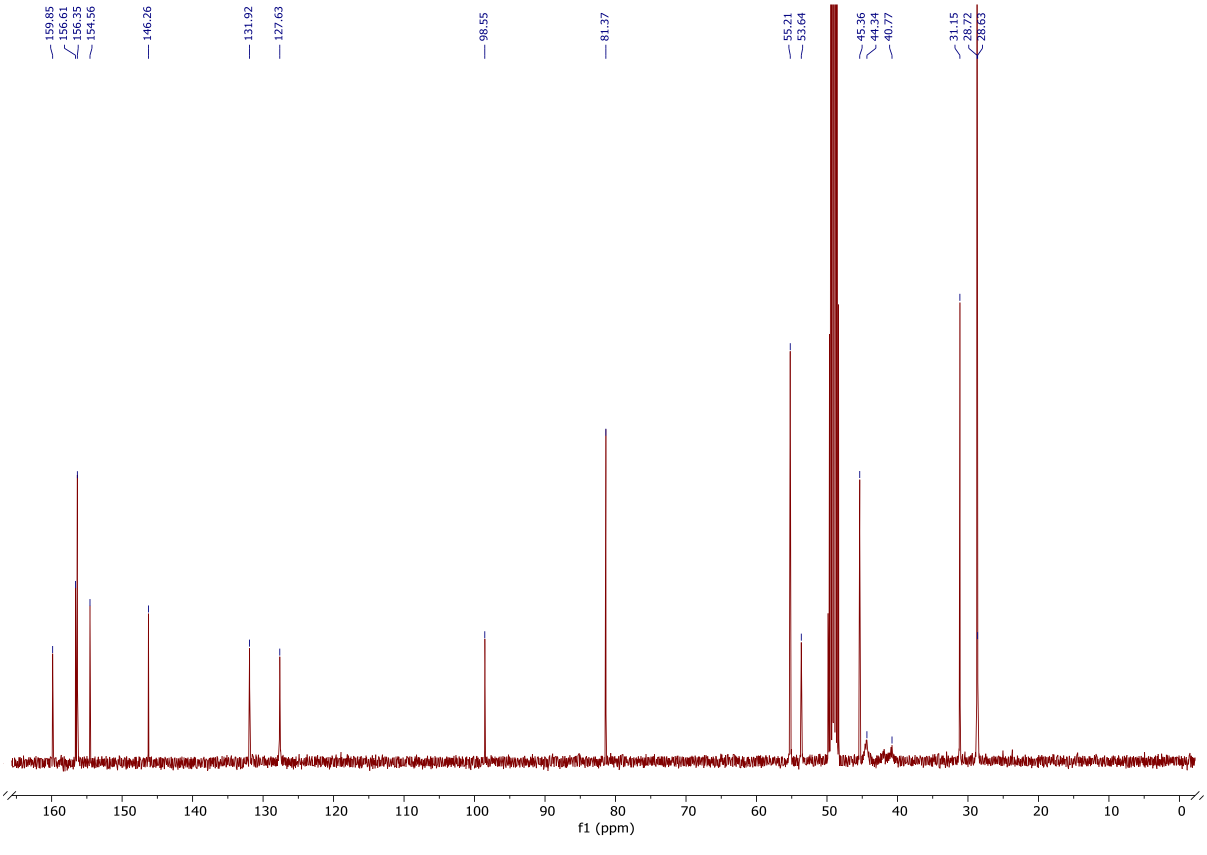
159.85
156.61
156.35
154.56
146.26
131.92
127.63
98.55
81.37
55.21
53.64
45.36
44.34
40.77
31.15
28.72
28.63
f1 (ppm)

1-(4-(4-amino-1-(1-methylpiperidin-4-yl)-1*H*-pyrazolo[3,4-*d*]pyrimidin-3-yl)-3,6-dihydropyridin - 1(2*H*)-yl)prop-2-en-1-one (12)

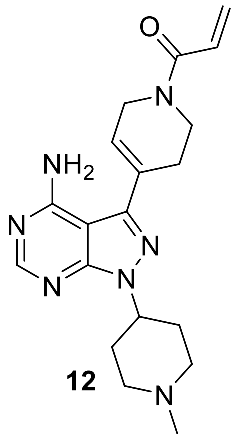

12

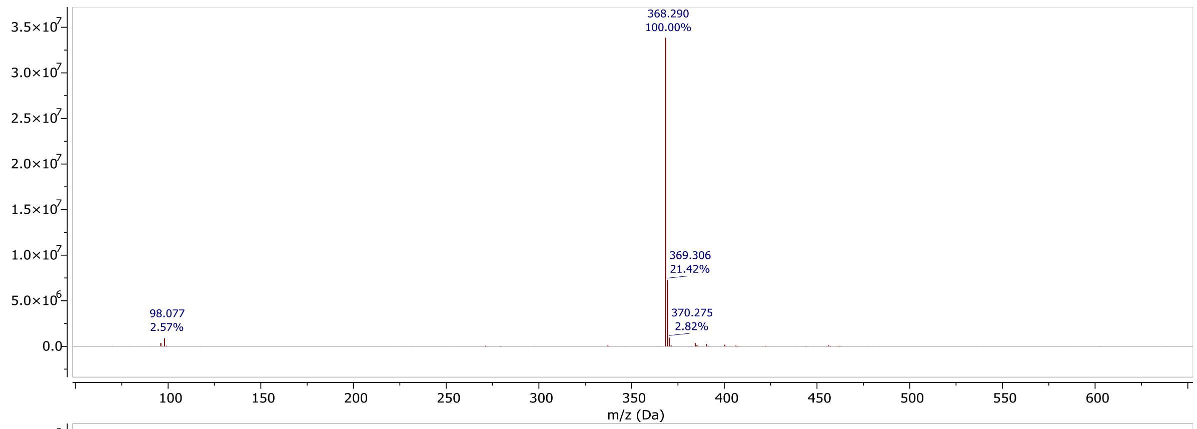

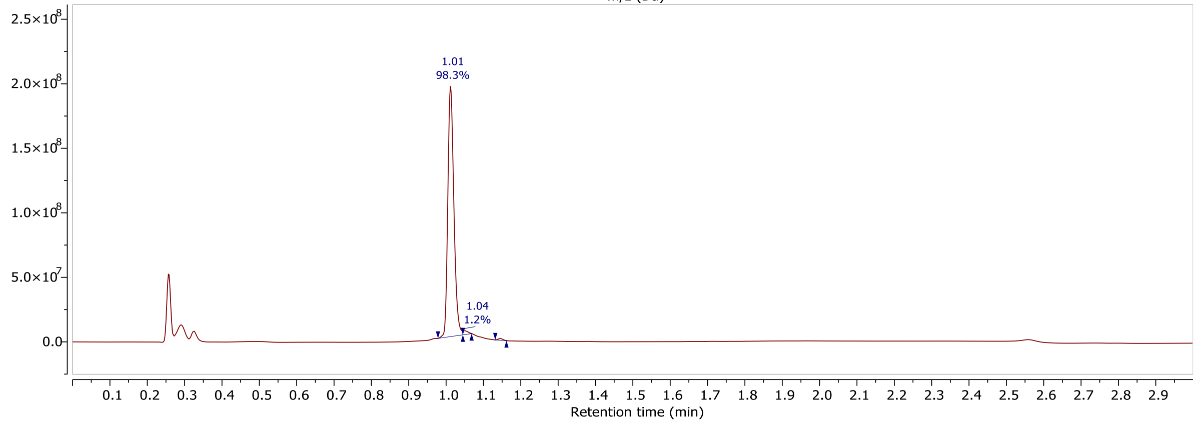

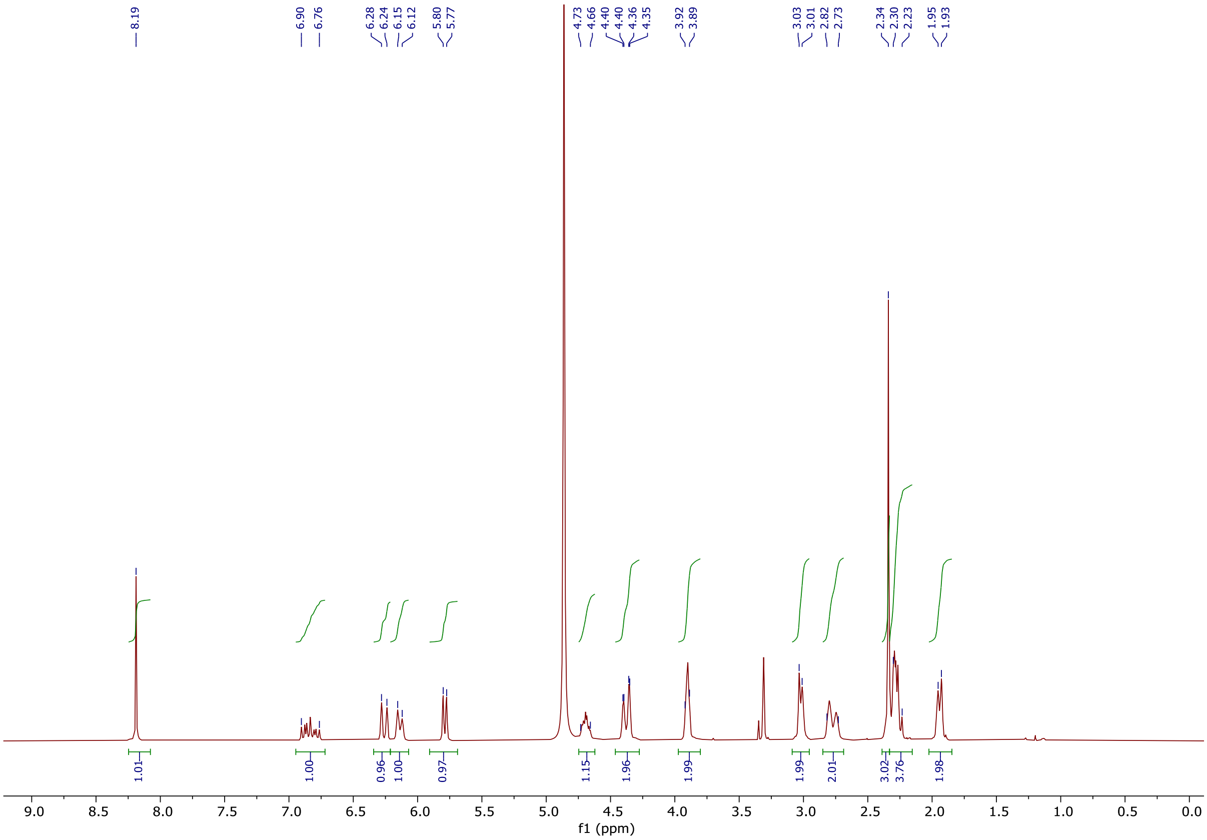
8.19
6.90 6.76
6.28 6.24 6.15 6.12
5.80 5.77
4.73 4.66 4.40 4.40 4.36 4.35
3.92 3.89
3.03 3.01 2.82 2.73
2.34 2.30 2.23
1.95 1.93
1.01 1.00 0.96 1.00 0.97 1.15 1.96 1.99 1.99 2.01 3.02 3.76 1.98
9.0 8.5 8.0 7.5 7.0 6.5 6.0 5.5 5.0 4.5 4.0 3.5 3.0 2.5 2.0 1.5 1.0 0.5 0.0
f1 (ppm)

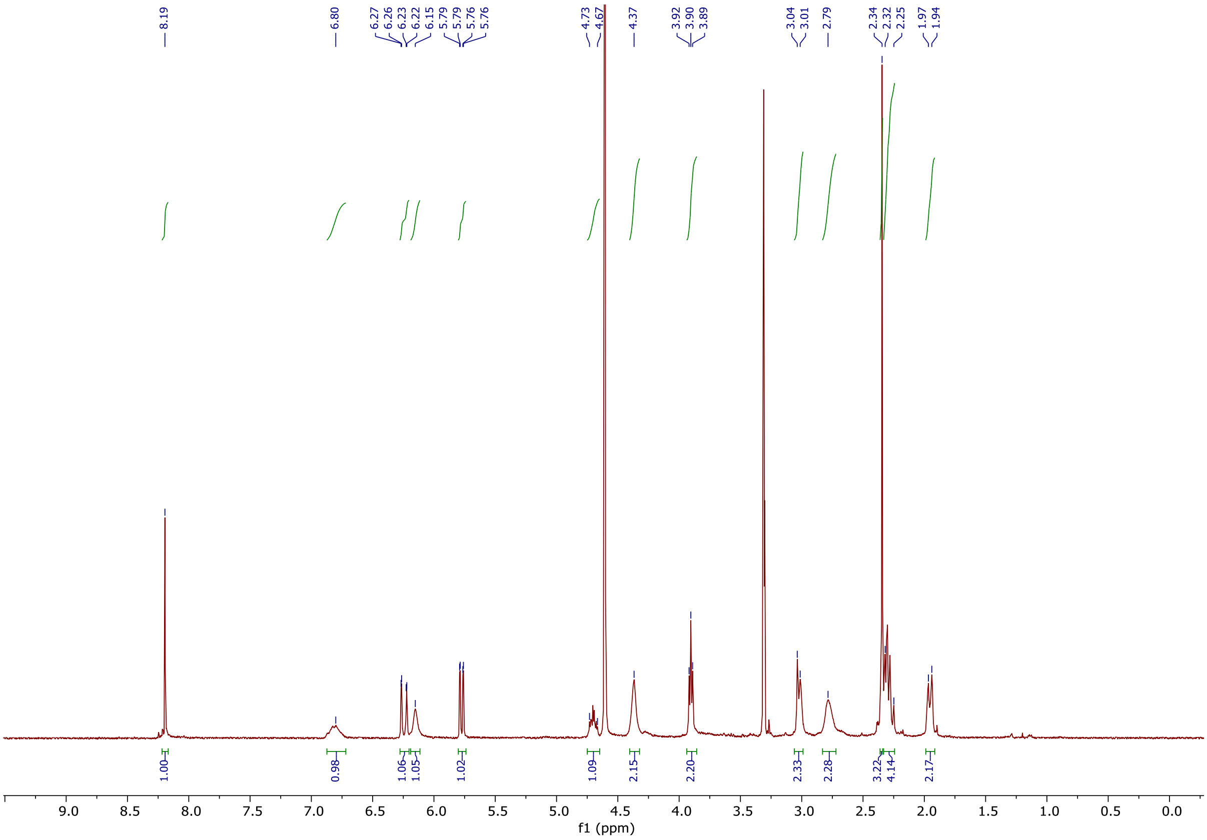
8.19
6.80
6.27 6.26 6.23 6.22 6.15 5.79 5.79 5.76 5.76
4.73 4.67
4.37
3.92 3.90 3.89
3.04 3.01 2.79
2.34 2.32 2.25
1.97 1.94
1.00 0.98 1.06 1.05 1.02 1.09 2.15 2.20 2.33 2.28 3.22 4.14 2.17
9.0 8.5 8.0 7.5 7.0 6.5 6.0 5.5 5.0 4.5 4.0 3.5 3.0 2.5 2.0 1.5 1.0 0.5 0.0
f1 (ppm)

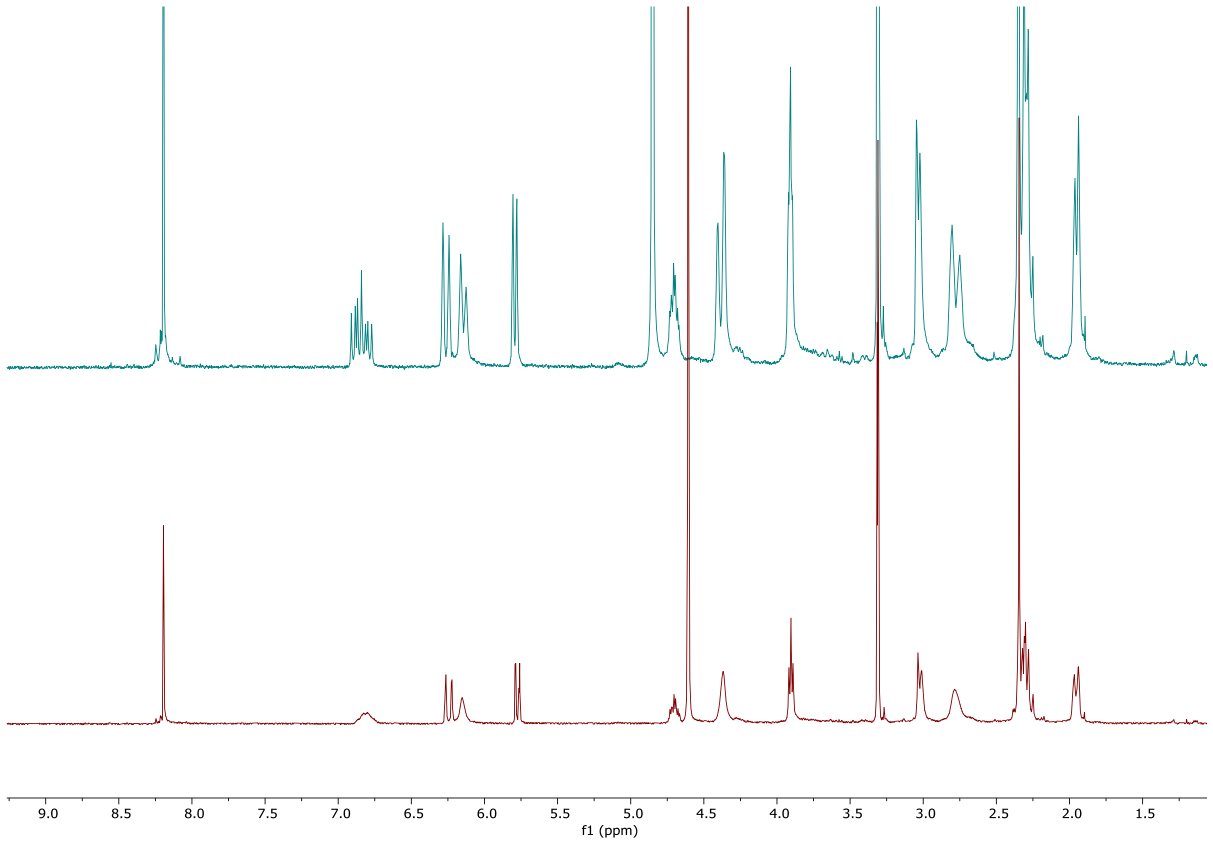

9.0
8.5
8.0
7.5
7.0
6.5
6.0
5.5
5.0
4.5
4.0
3.5
3.0
2.5
2.0
1.5
f1 (ppm)

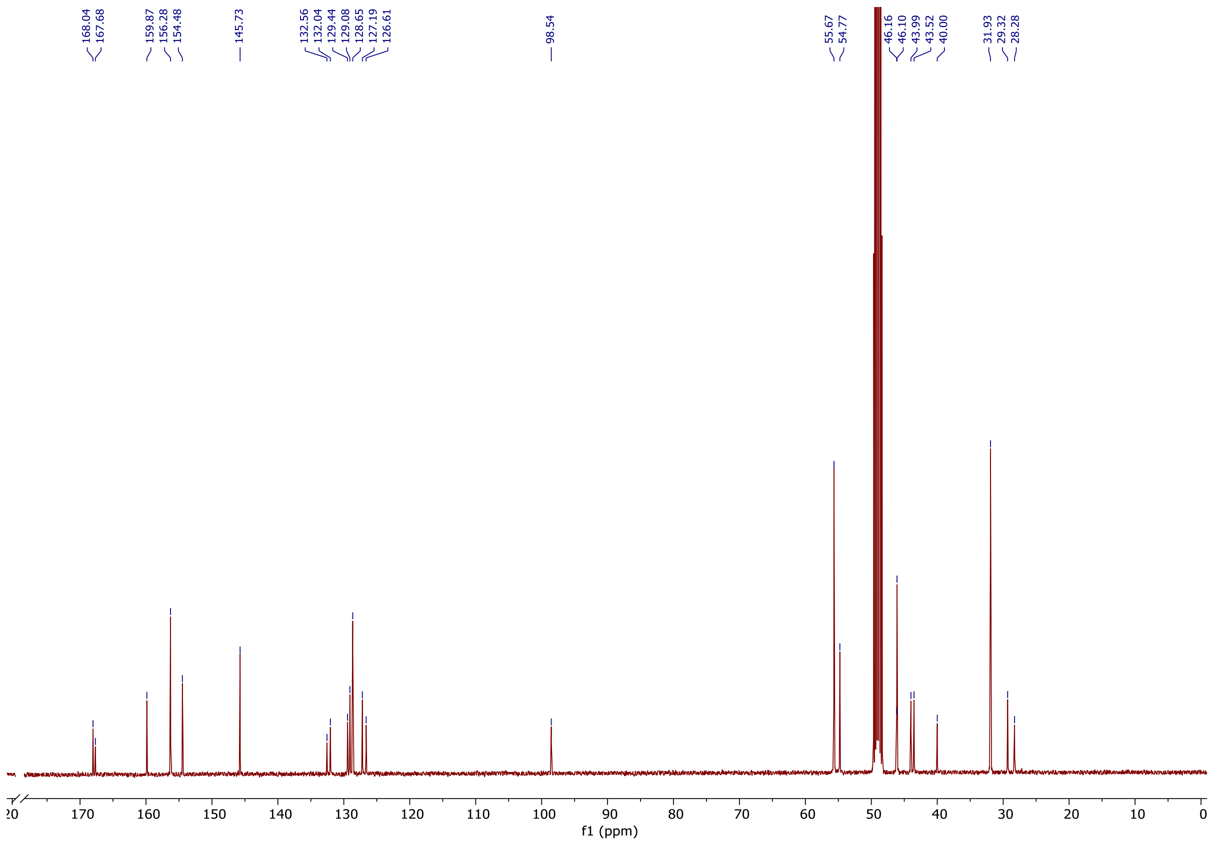

168.04
167.68
159.87
156.28
154.48
145.73
132.56
132.04
129.44
129.08
128.65
127.19
126.61
98.54
55.67
54.77
46.16
46.10
43.99
43.52
40.00
31.93
29.32
28.28
170
160
150
140
130
120
110
100
90
80
70
60
50
40
30
20
10
0
f1 (ppm)

***Tert*-butyl 5-(4-amino-1-(1-methylpiperidin-4-yl)-1*H*-pyrazolo[3,4-*d*]pyrimidin-3-yl)-3,6-dihydropyridine-1(2*H*)-carboxylate (18b)**

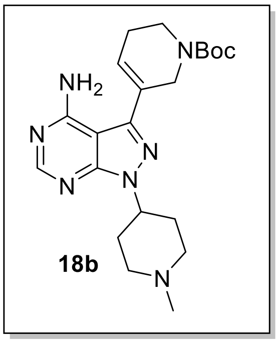

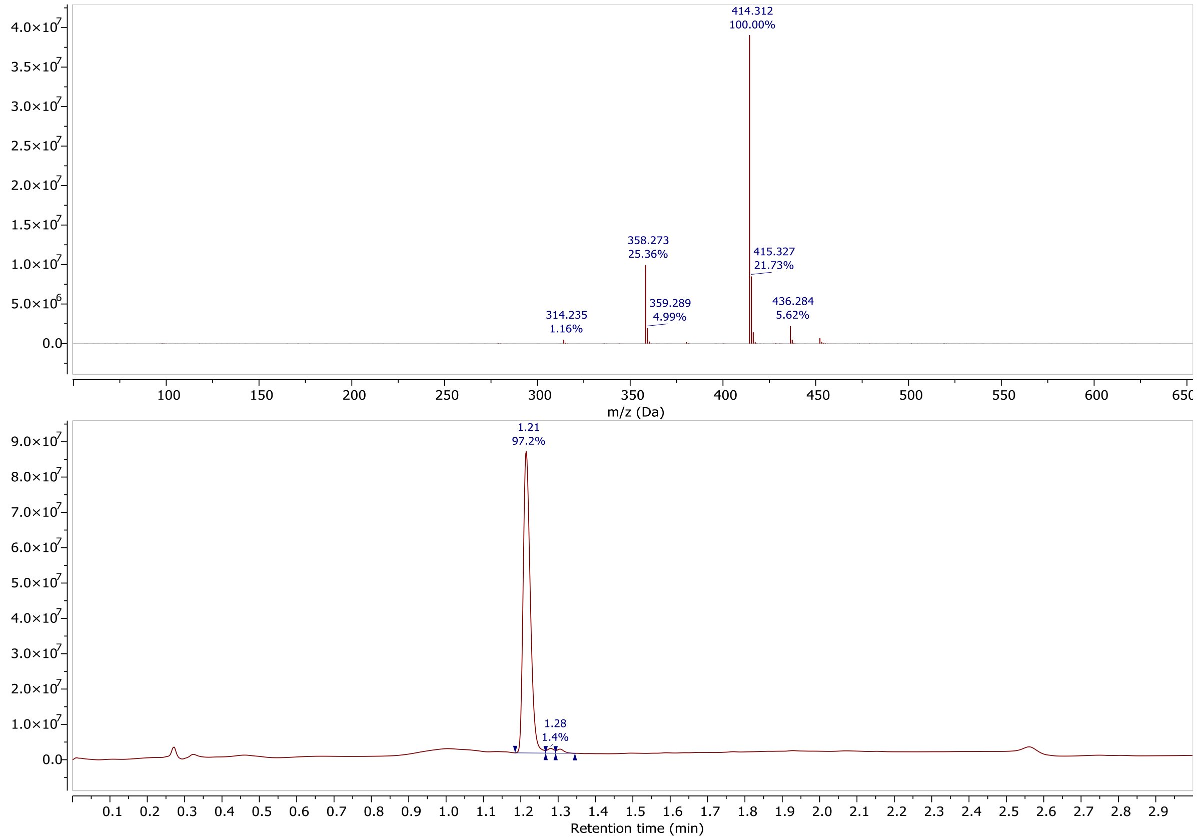

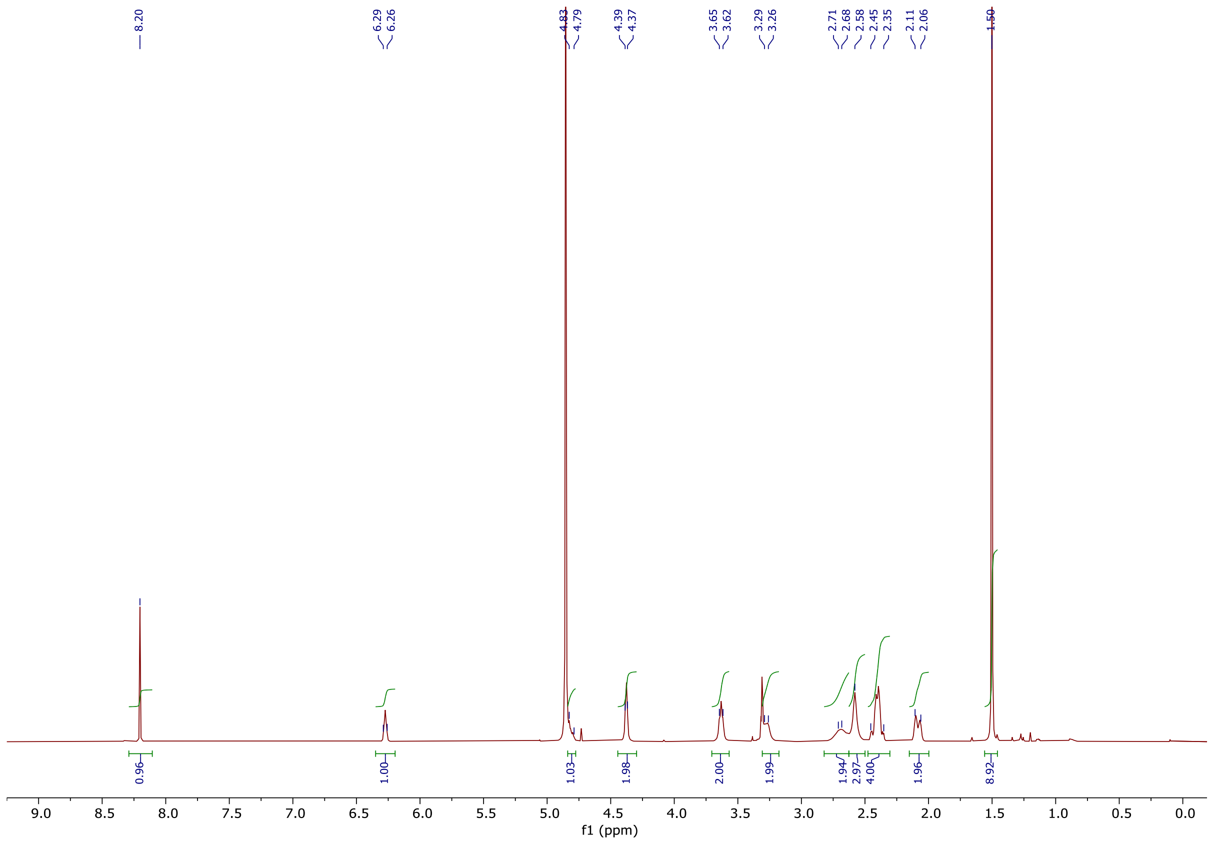
8.20
6.29
6.26
4.83
4.79
4.39
4.37
3.65
3.62
3.29
3.26
2.71
2.68
2.58
2.45
2.35
2.11
2.06
1.50
0.96
1.00
1.03
1.98
2.00
1.99
1.94
2.97
4.00
1.96
8.92
f1 (ppm)

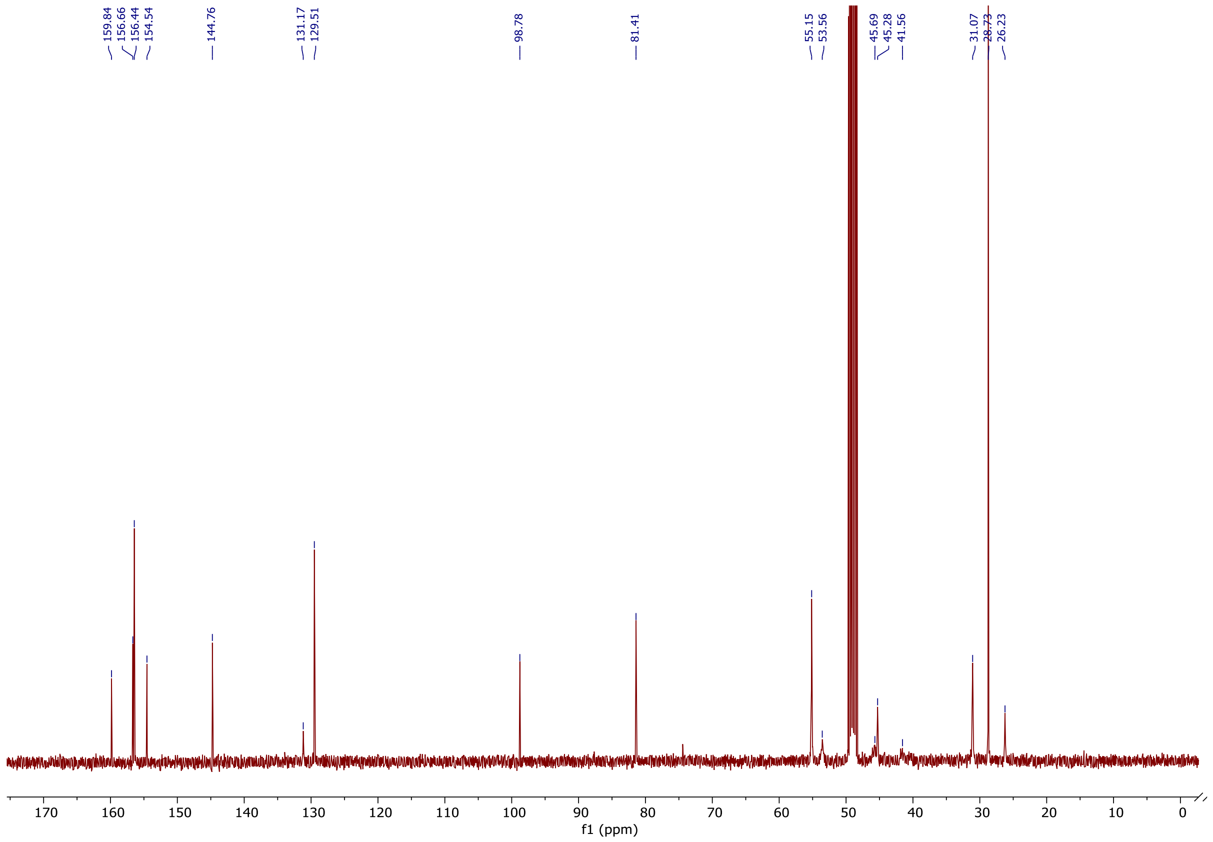
159.84
156.66
156.44
154.54
144.76
131.17
129.51
98.78
81.41
55.15
53.56
45.69
45.28
41.56
31.07
28.73
26.23
f1 (ppm)

1-(5-(4-amino-1-(1-methylpiperidin-4-yl)-1*H*-pyrazolo[3,4-*d*]pyrimidin-3-yl)-3,6-dihydropyridin - 1(2*H*)-yl)prop-2-en-1-one (13)

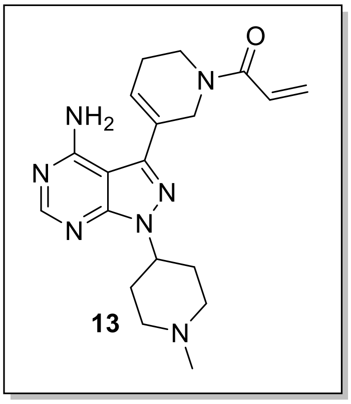

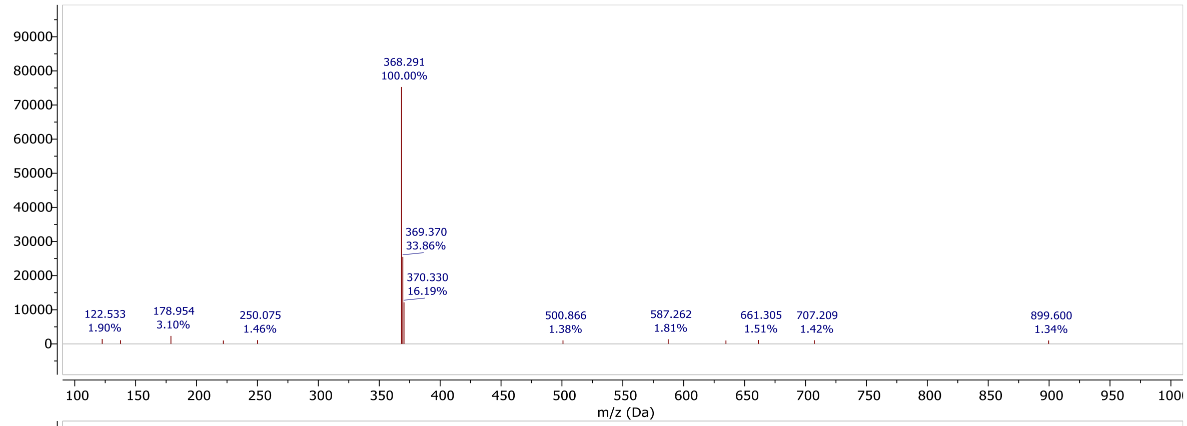

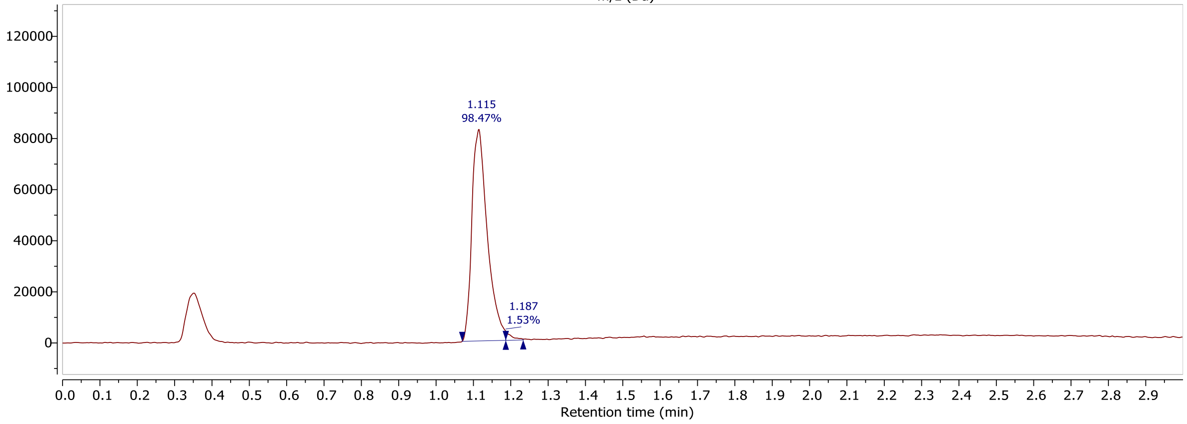

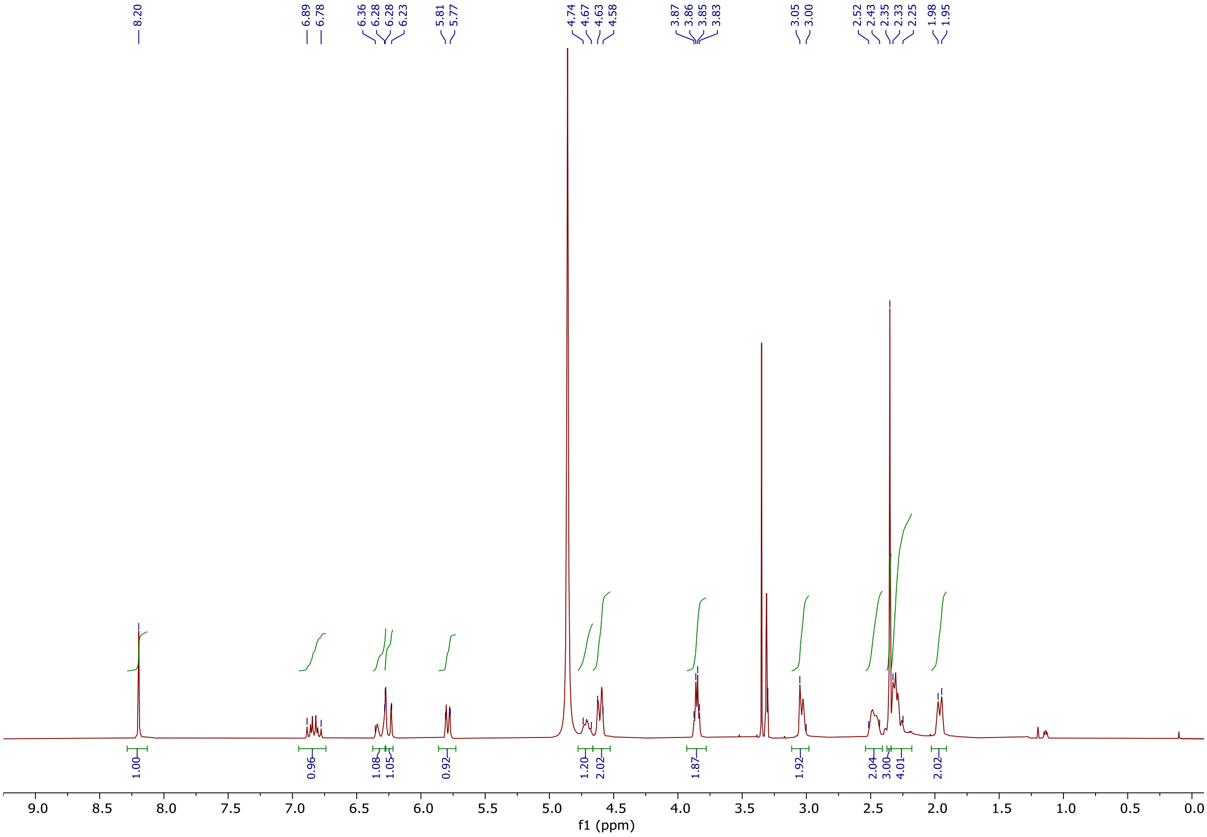
8.20
6.89
6.78
6.36
6.28
6.28
6.23
5.81
5.77
4.74
4.67
4.63
4.58
3.87
3.86
3.85
3.83
3.05
3.00
2.52
2.43
2.35
2.33
2.25
1.98
1.95
1.00
0.96
1.08
1.05
0.92
1.20
2.02
1.87
1.92
2.04
3.00
4.01
2.02
9.0 8.5 8.0 7.5 7.0 6.5 6.0 5.5 5.0 4.5 4.0 3.5 3.0 2.5 2.0 1.5 1.0 0.5 0.0
f1 (ppm)

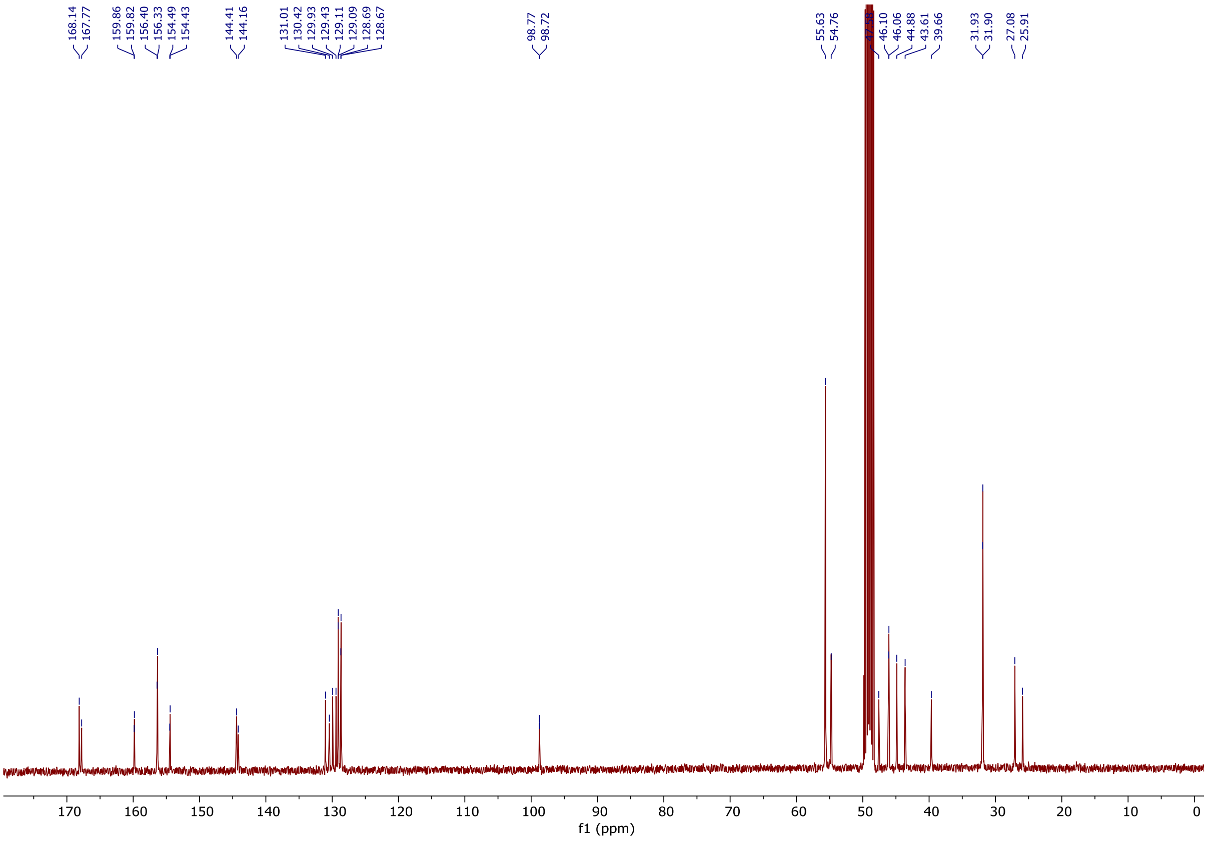
168.14
167.77
159.86
159.82
156.40
156.33
154.49
154.43
144.41
144.16
131.01
130.42
129.93
129.43
129.11
129.09
128.69
128.67
98.77
98.72
55.63
54.76
46.10
46.06
44.88
43.61
39.66
31.93
31.90
27.08
25.91
170 160 150 140 130 120 110 100 90 80 70 60 50 40 30 20 10 0
f1 (ppm)

Tert-butyl (4-(4-amino-1-(1-methylpiperidin-4-yl)-1H-pyrazolo[3,4-d]pyrimidin-3-yl)benzyl) carbamate (18c)

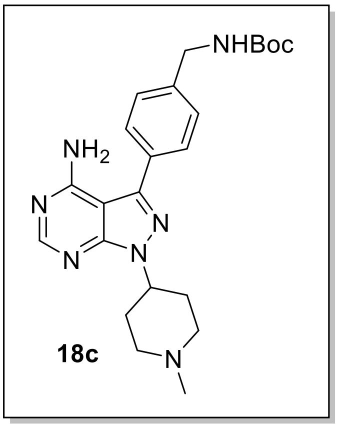

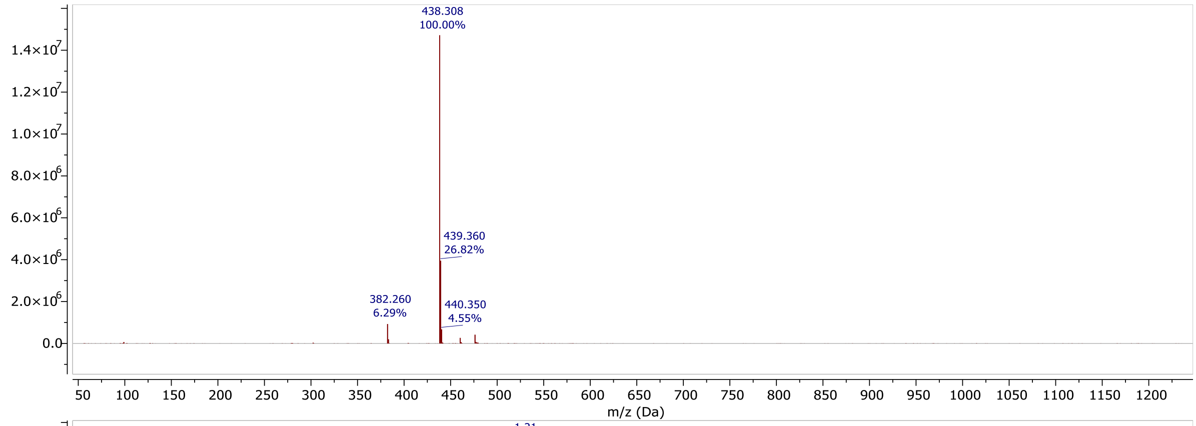

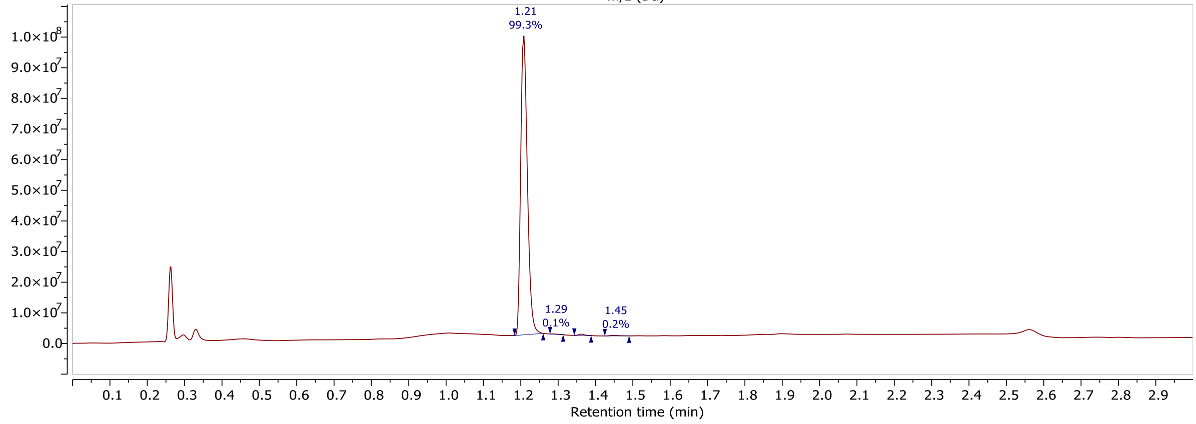

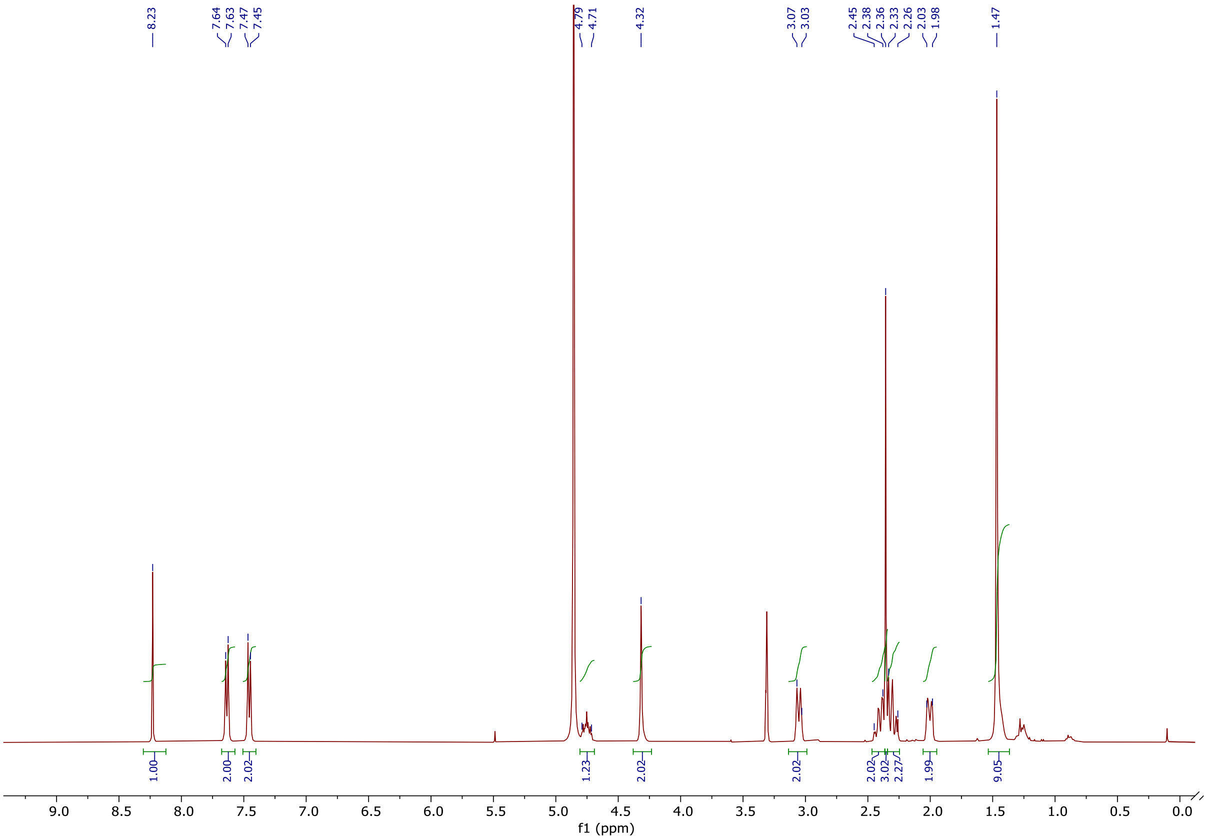
8.23
7.64
7.63
7.47
7.45
4.79
4.71
4.32
3.07
3.03
2.45
2.38
2.36
2.33
2.26
2.03
1.98
1.47
1.00
2.00
2.02
1.23
2.02
2.02
2.02
3.02
2.27
1.99
9.05
f1 (ppm)

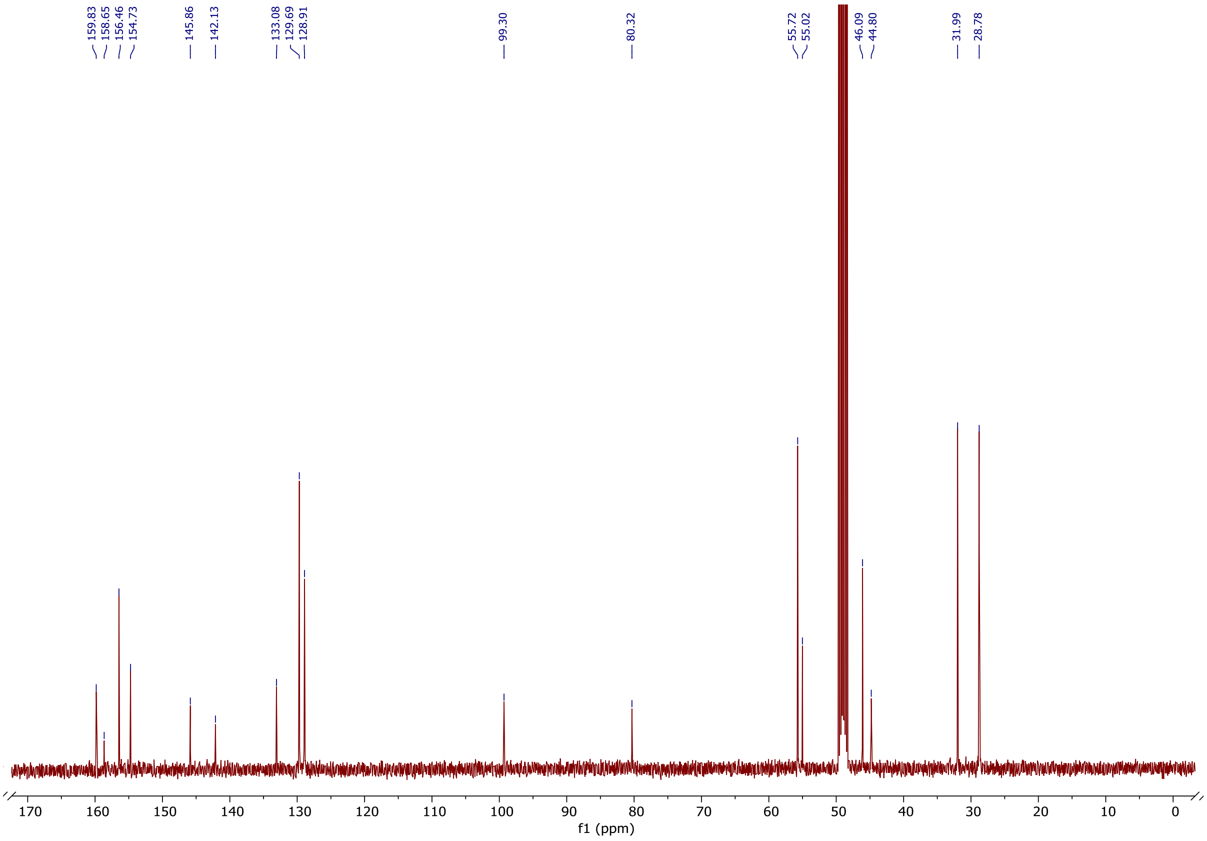
159.83
158.65
156.46
154.73
145.86
142.13
133.08
129.69
128.91
99.30
80.32
55.72
55.02
46.09
44.80
31.99
28.78
f1 (ppm)

N-(4-(4-amino-1-(1-methylpiperidin-4-yl)-1H-pyrazolo[3,4-d]pyrimidin-3-yl)benzyl)acrylamide (14)

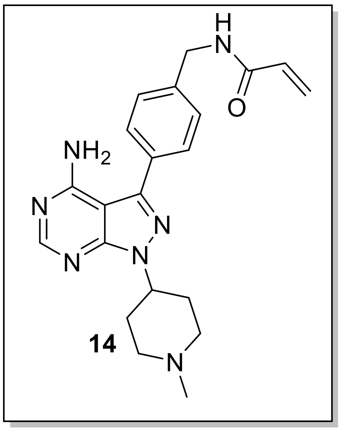

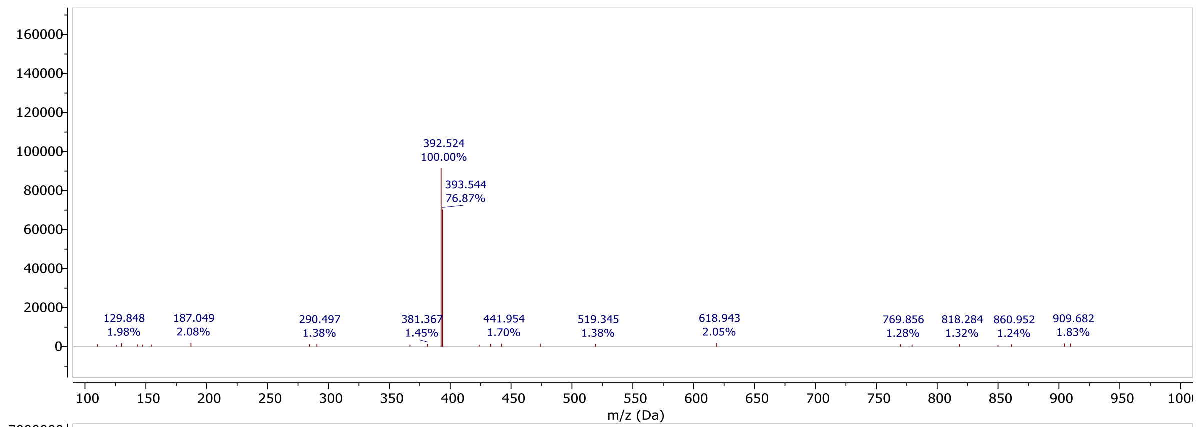

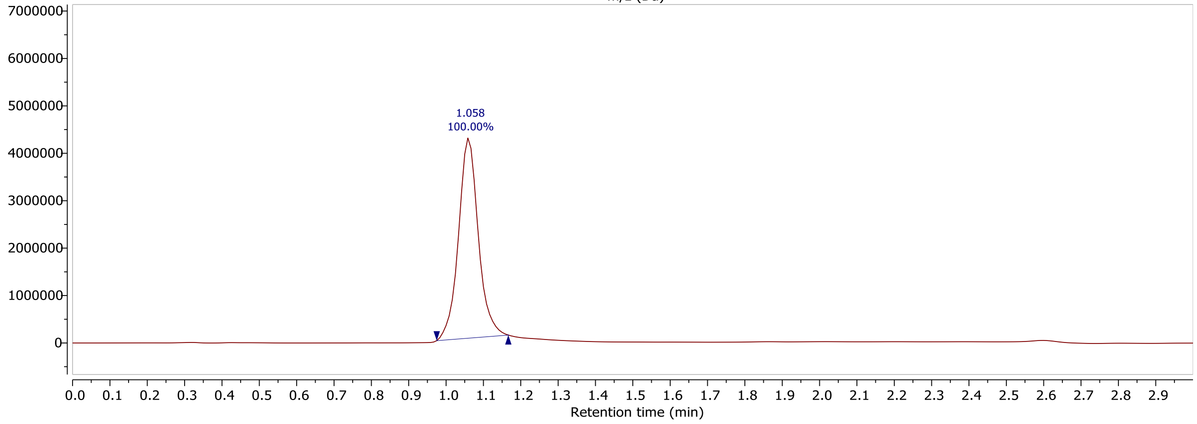

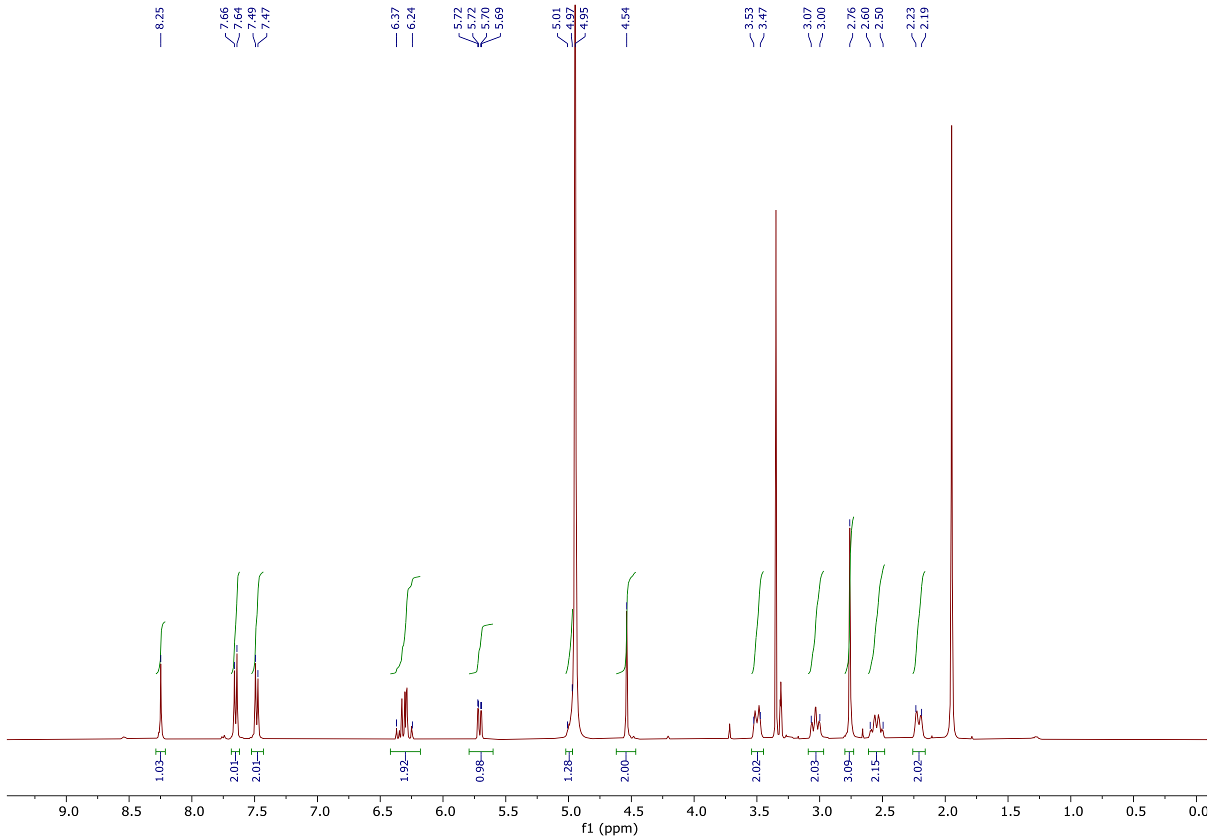

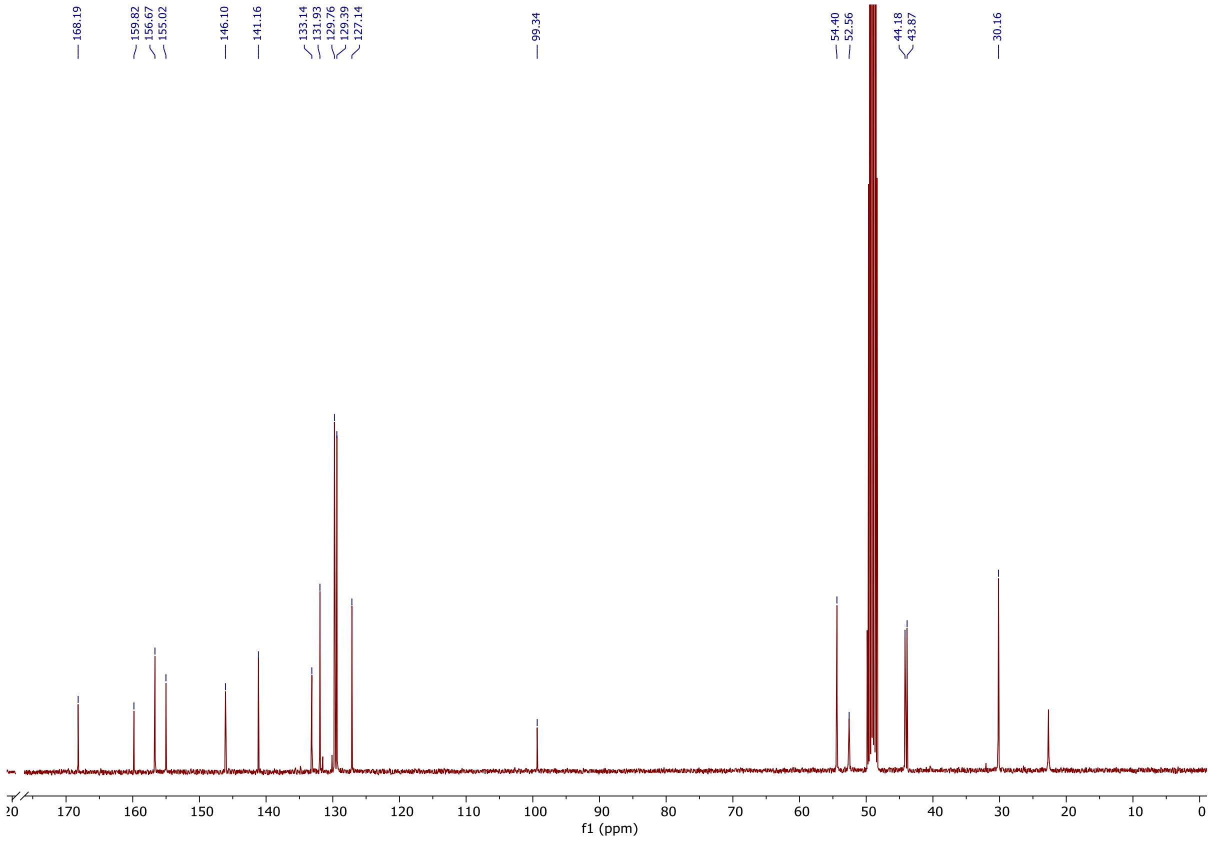

***Tert*-butyl 4-(4-amino-1-(1-methylpiperidin-4-yl)-1*H*-pyrazolo[3,4-*d*]pyrimidin-3-yl)piperidine-1-carboxylate (19)**

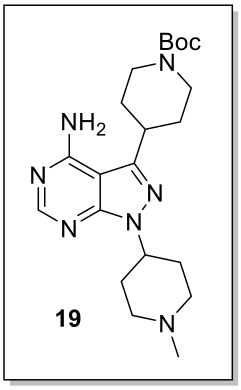

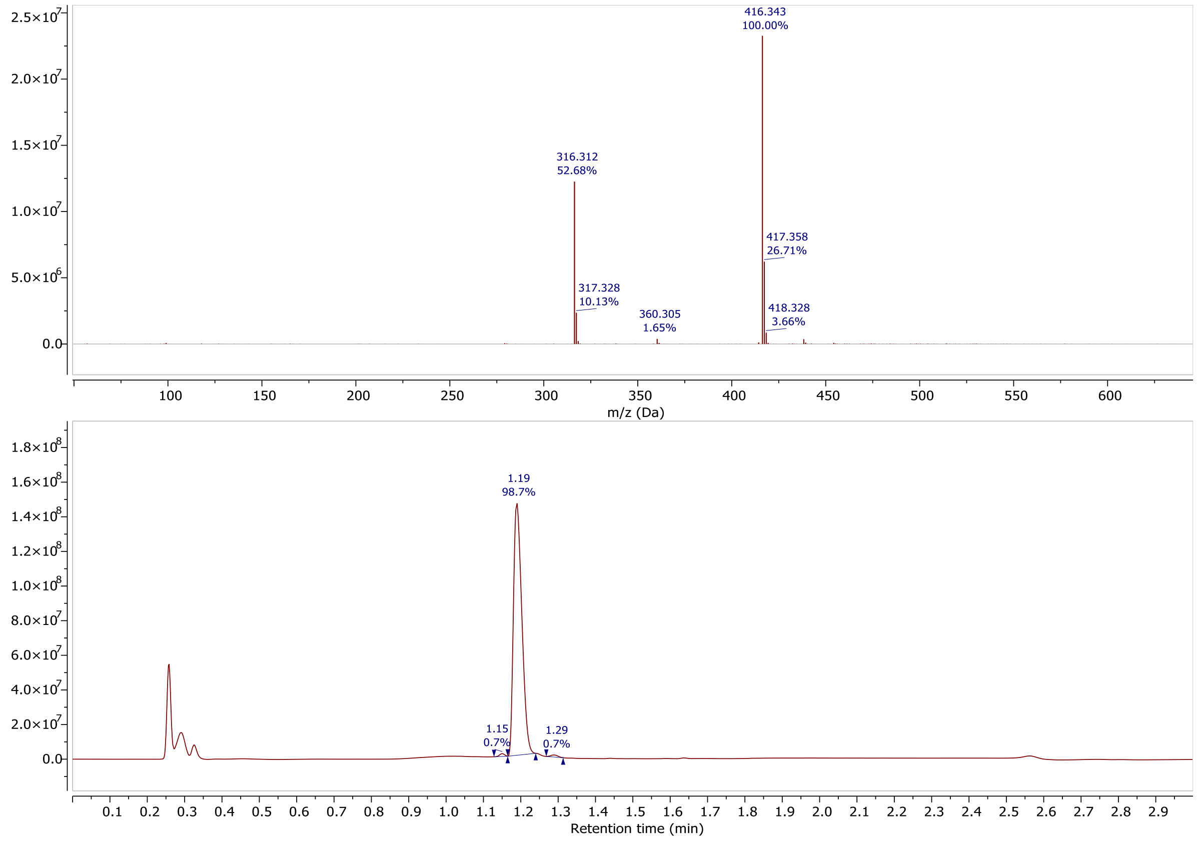

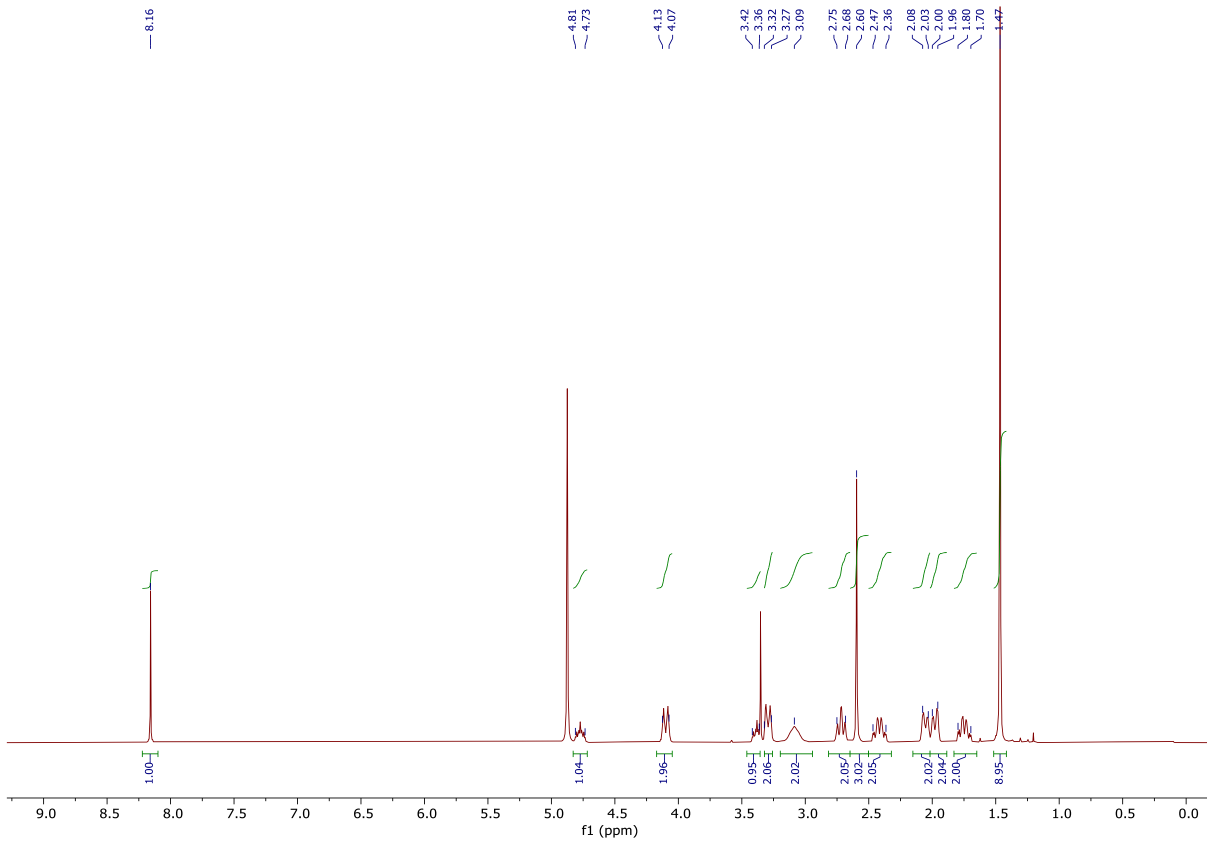
8.16
4.81
4.73
4.13
4.07
3.42
3.36
3.32
3.27
3.09
2.75
2.68
2.60
2.47
2.36
2.08
2.03
2.00
1.96
1.80
1.70
1.47
1.00
1.04
1.96
0.95
2.06
2.02
2.05
3.02
2.05
2.02
2.04
2.00
8.95
f1 (ppm)

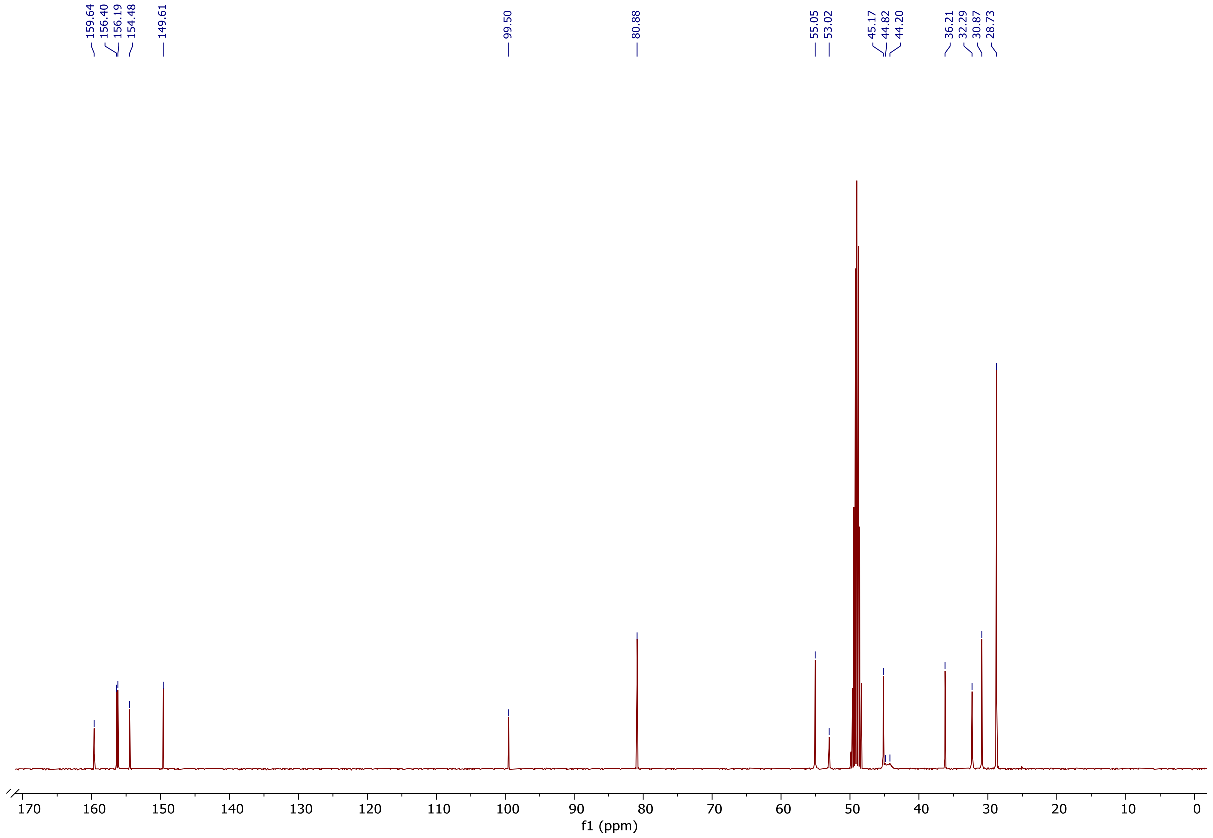
159.64
156.40
156.19
154.48
149.61
99.50
80.88
55.05
53.02
45.17
44.82
44.20
36.21
32.29
30.87
28.73
f1 (ppm)

1-(4-(4-amino-1-(1-methylpiperidin-4-yl)-1*H*-pyrazolo[3,4-*d*]pyrimidin-3-yl)piperidin-1-yl)prop-2-en-1-one (15)

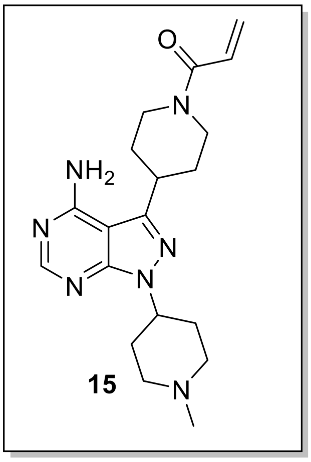

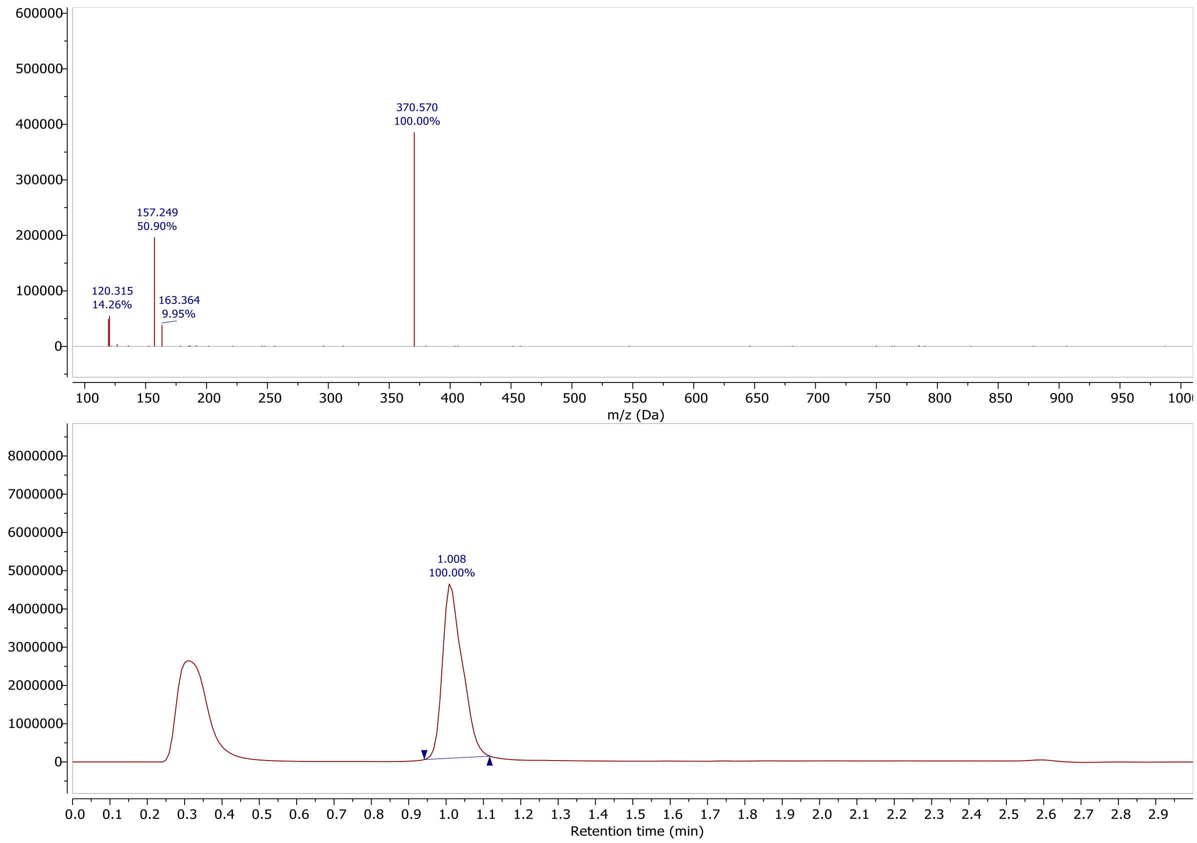

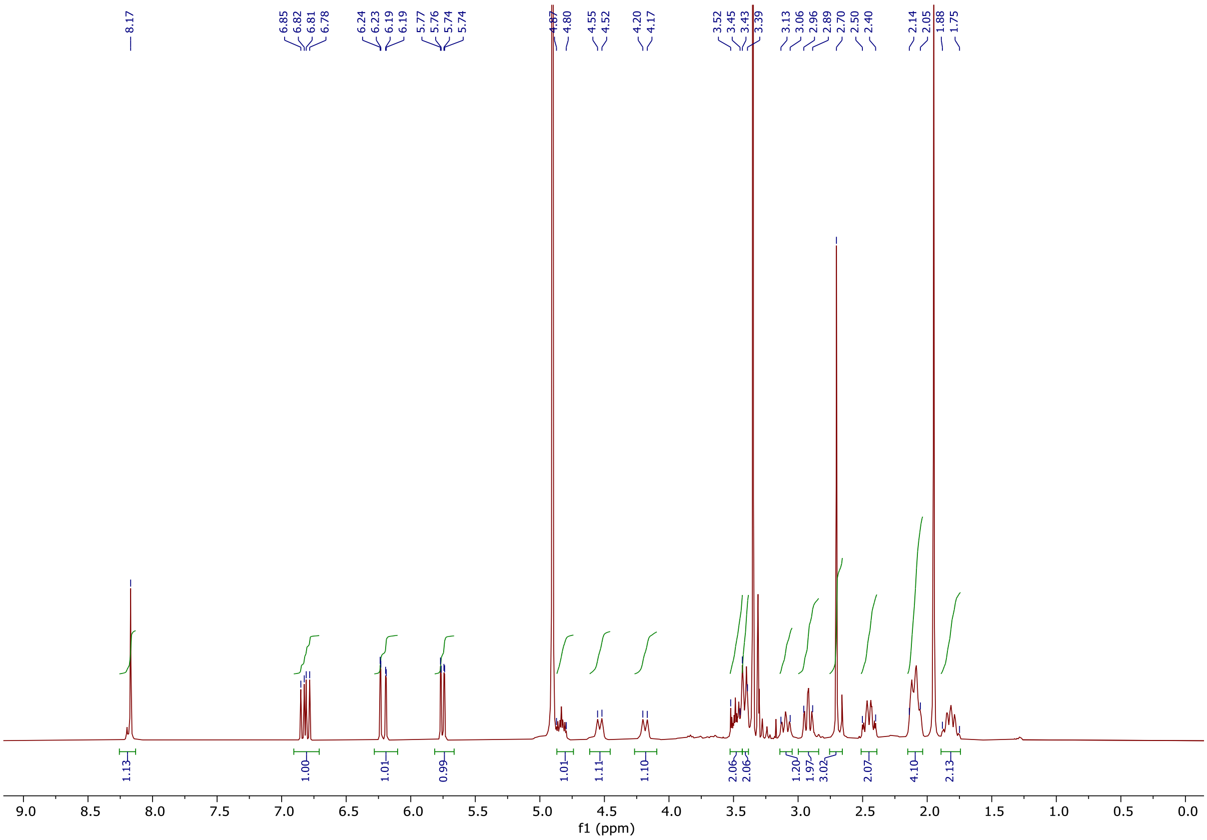

8.17
6.85
6.82
6.81
6.78
6.24
6.23
6.19
6.19
5.77
5.76
5.74
5.74
4.87
4.80
4.55
4.52
4.20
4.17
3.52
3.45
3.43
3.39
3.13
3.06
2.96
2.89
2.70
2.50
2.40
2.14
2.05
1.88
1.75
1.13
1.00
1.01
0.99
1.01
1.11
1.10
2.06
2.06
1.20
1.97
3.02
2.07
4.10
2.13
f1 (ppm)

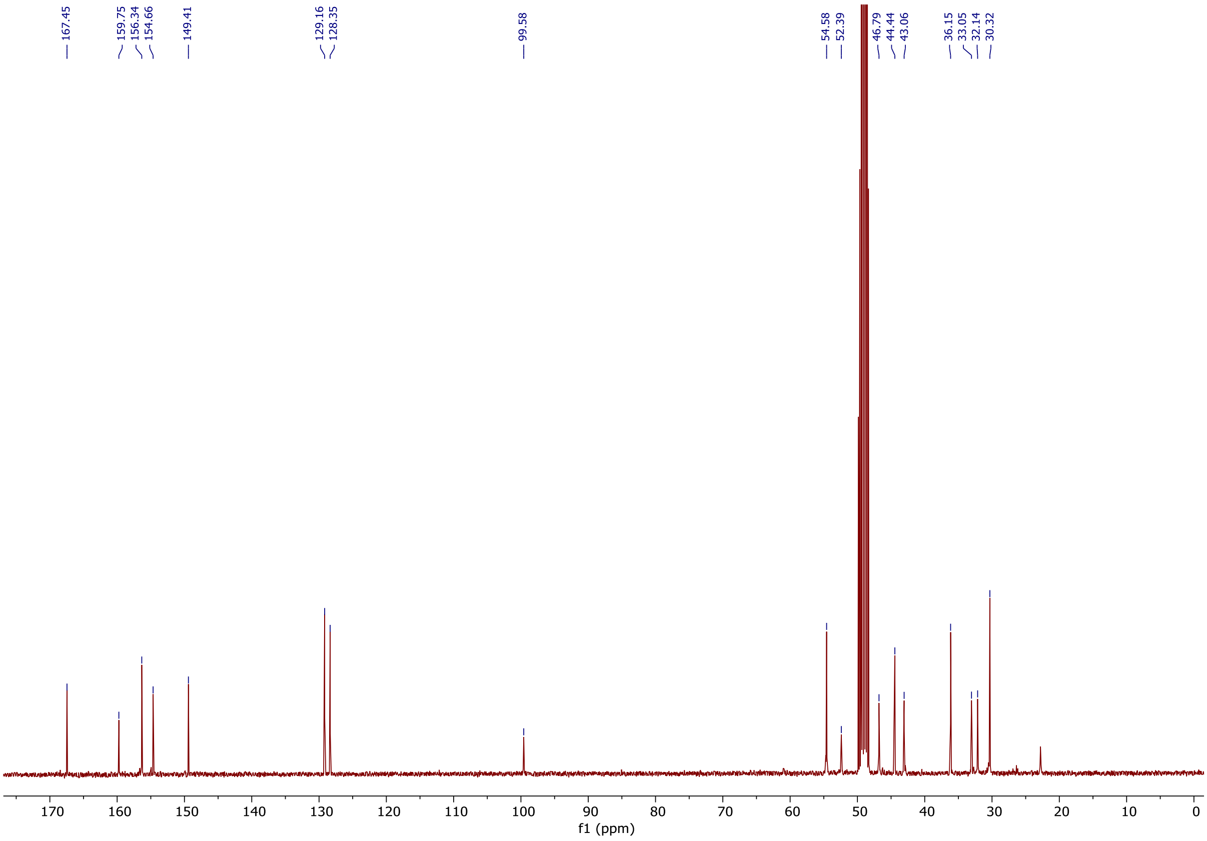

167.45
159.75
156.34
154.66
149.41
129.16
128.35
99.58
54.58
52.39
46.79
44.44
43.06
36.15
33.05
32.14
30.32
f1 (ppm)

Tert-butyl (4-(4-(7-((5-(3,4-dichlorophenyl)-1,3,4-oxadiazol-2-yl)methyl)-1,3-dimethyl-2,6-dioxo-2,3,6,7-tetrahydro-1_H_-purin-8-yl)piperazin-1-yl)butyl)carbamate

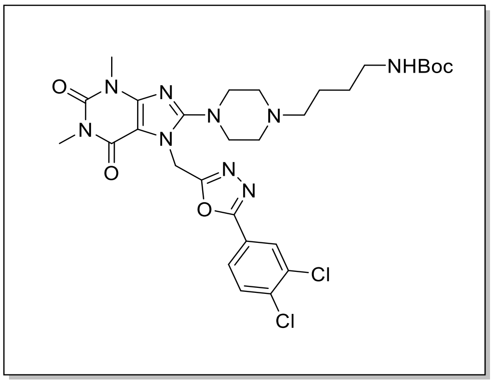

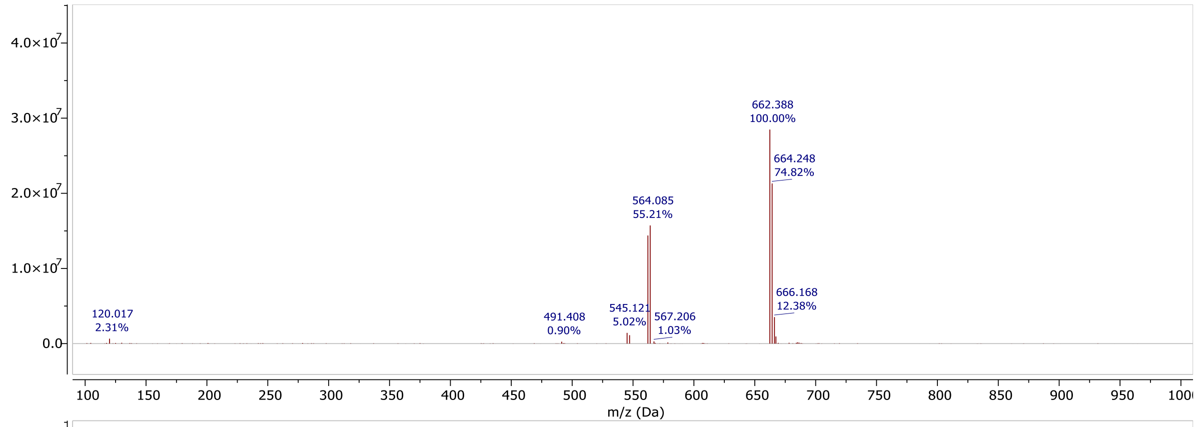

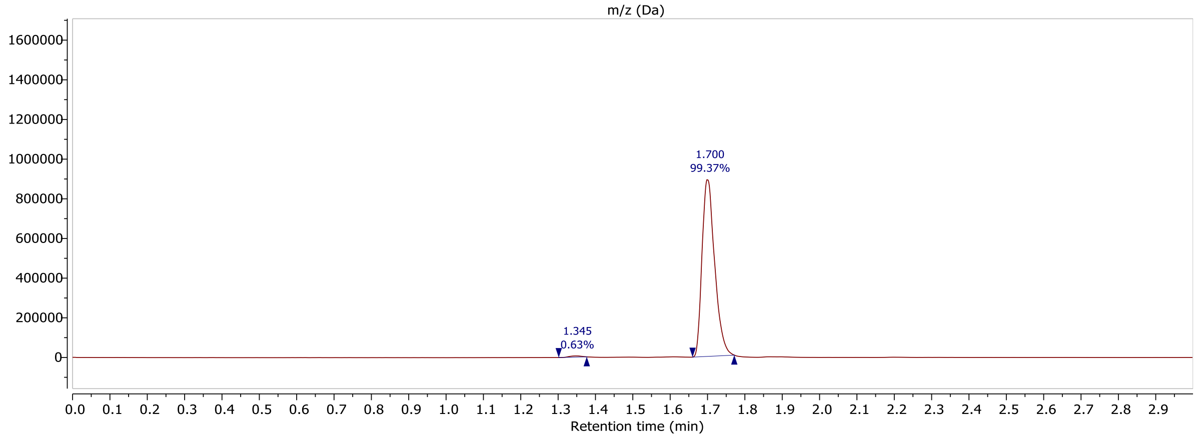

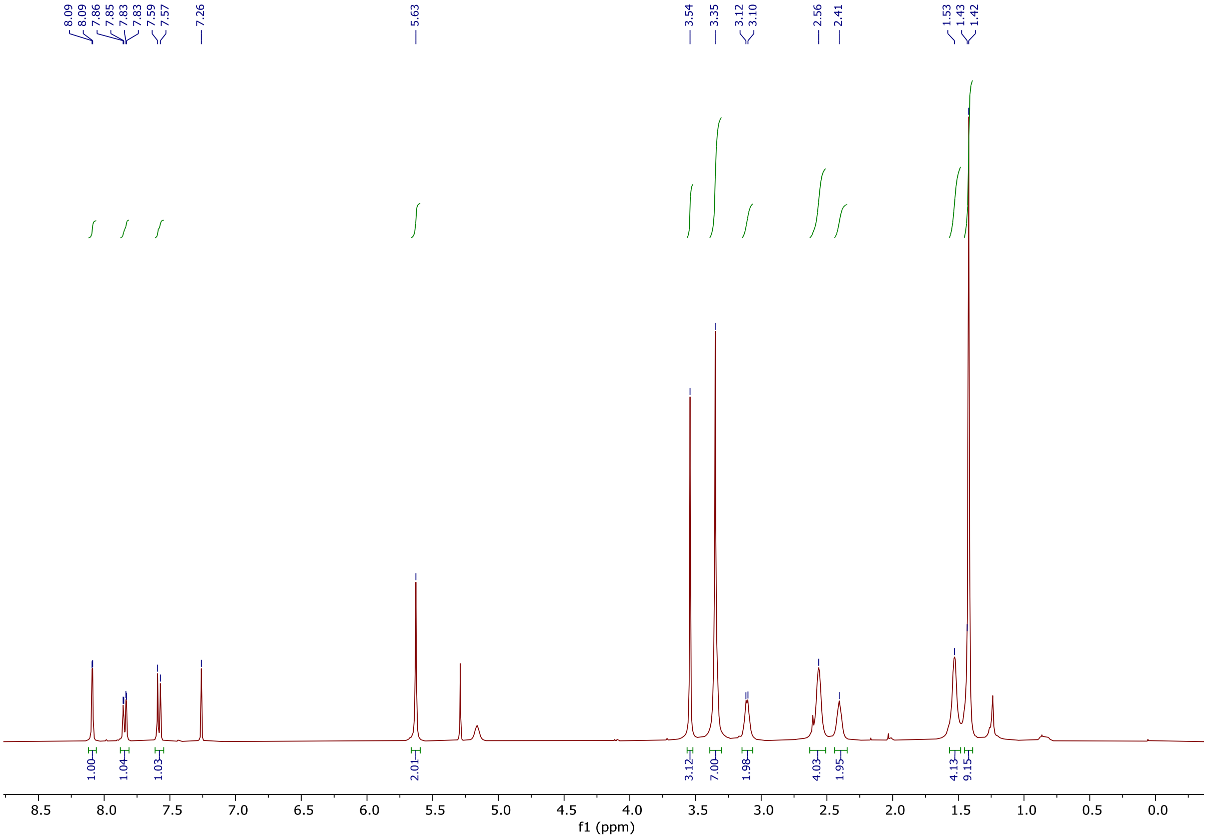

8.09
8.09
7.86
7.85
7.83
7.83
7.59
7.57
7.26
5.63
3.54
3.35
3.12
3.10
2.56
2.41
1.53
1.43
1.42
1.00
1.04
1.03
2.01
3.12
7.00
1.98
4.03
1.95
4.13
9.15
f1 (ppm)

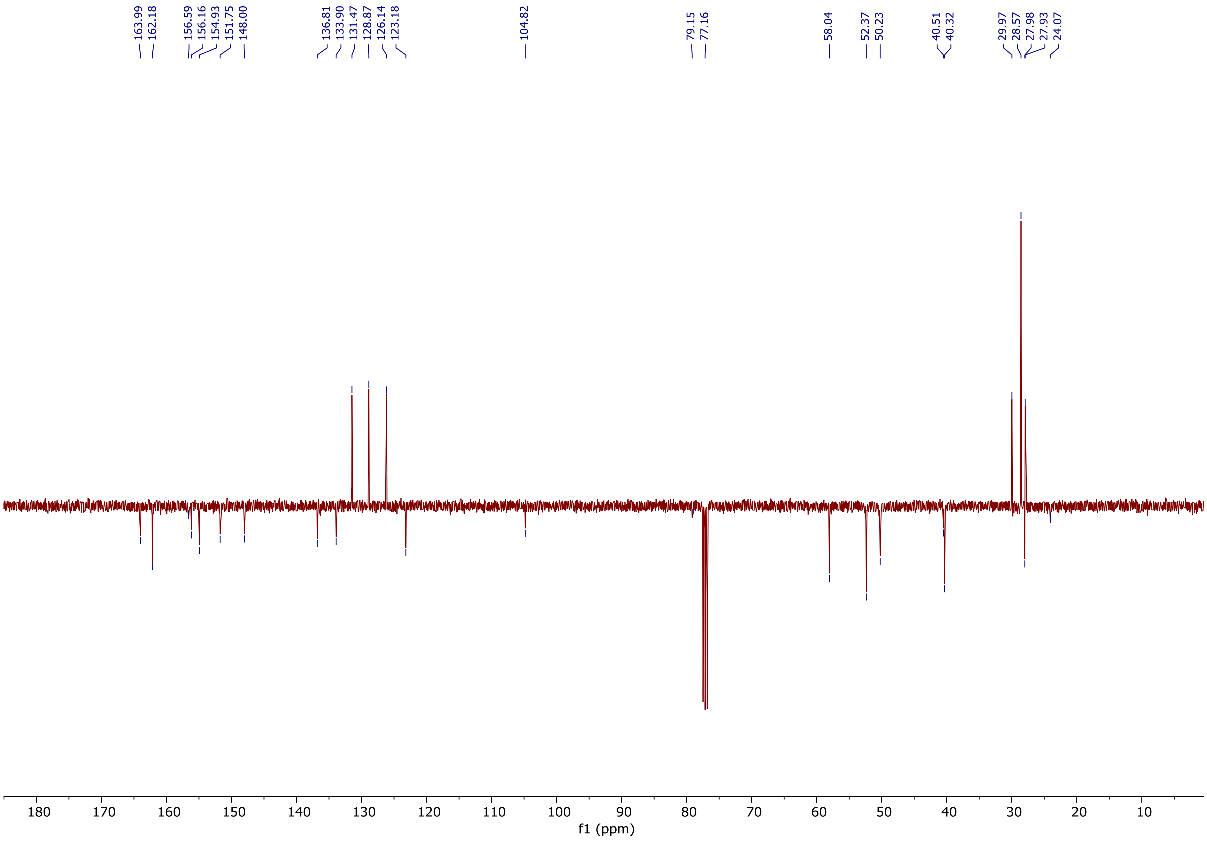

163.99
162.18
156.59
156.16
154.93
151.75
148.00
136.81
133.90
131.47
128.87
126.14
123.18
104.82
79.15
77.16
58.04
52.37
50.23
40.51
40.32
29.97
28.57
27.98
27.93
24.07
f1 (ppm)

4-(4-(7-((5-(3,4-dichlorophenyl)-1,3,4-oxadiazol-2-yl)methyl)-1,3-dimethyl-2,6-dioxo-2,3,6,7-tetrahydro-1*H*-purin-8-yl)piperazin-1-yl)butan-1-aminium trifluoroacetate, CBH-003 (7)

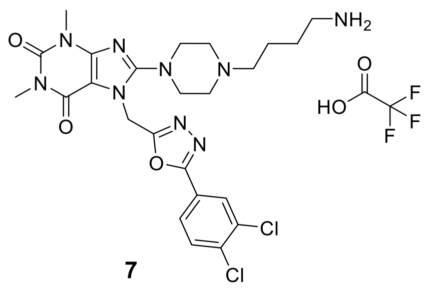

7

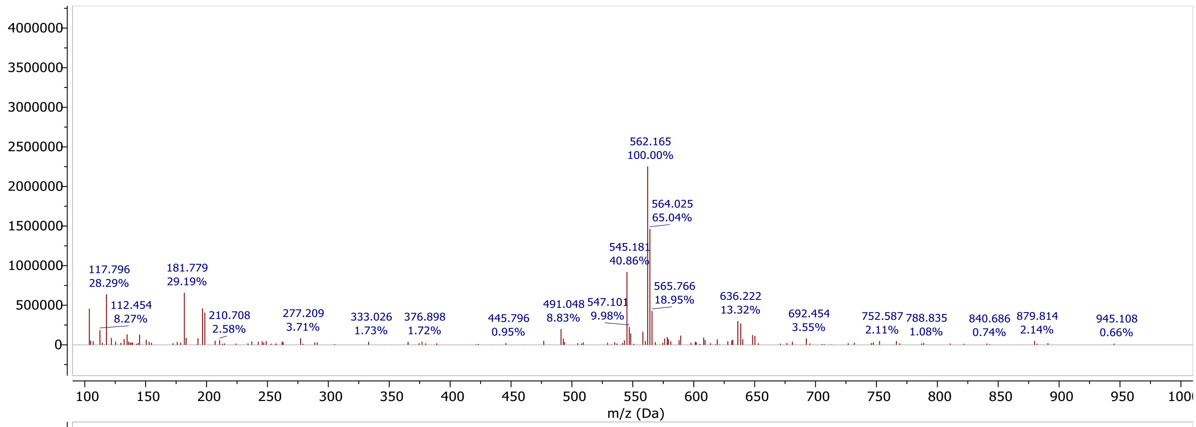

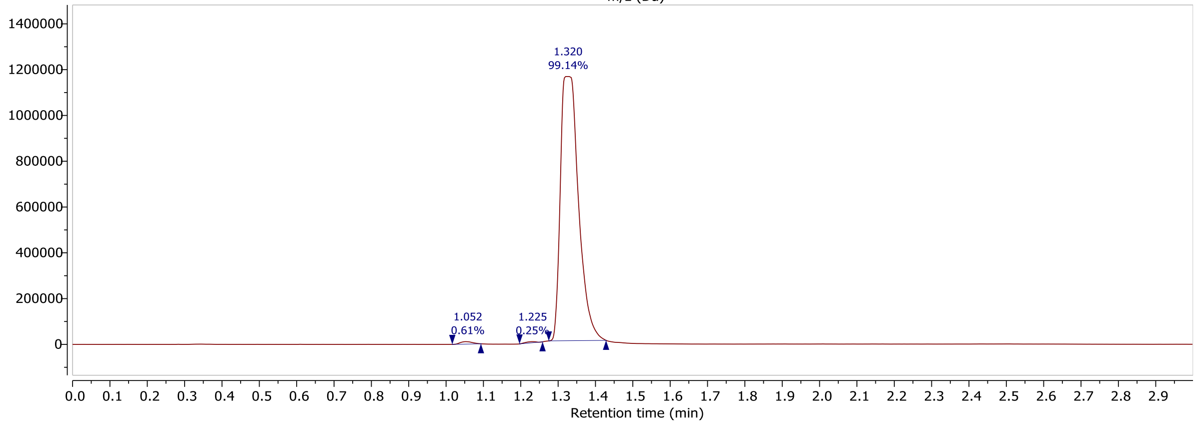

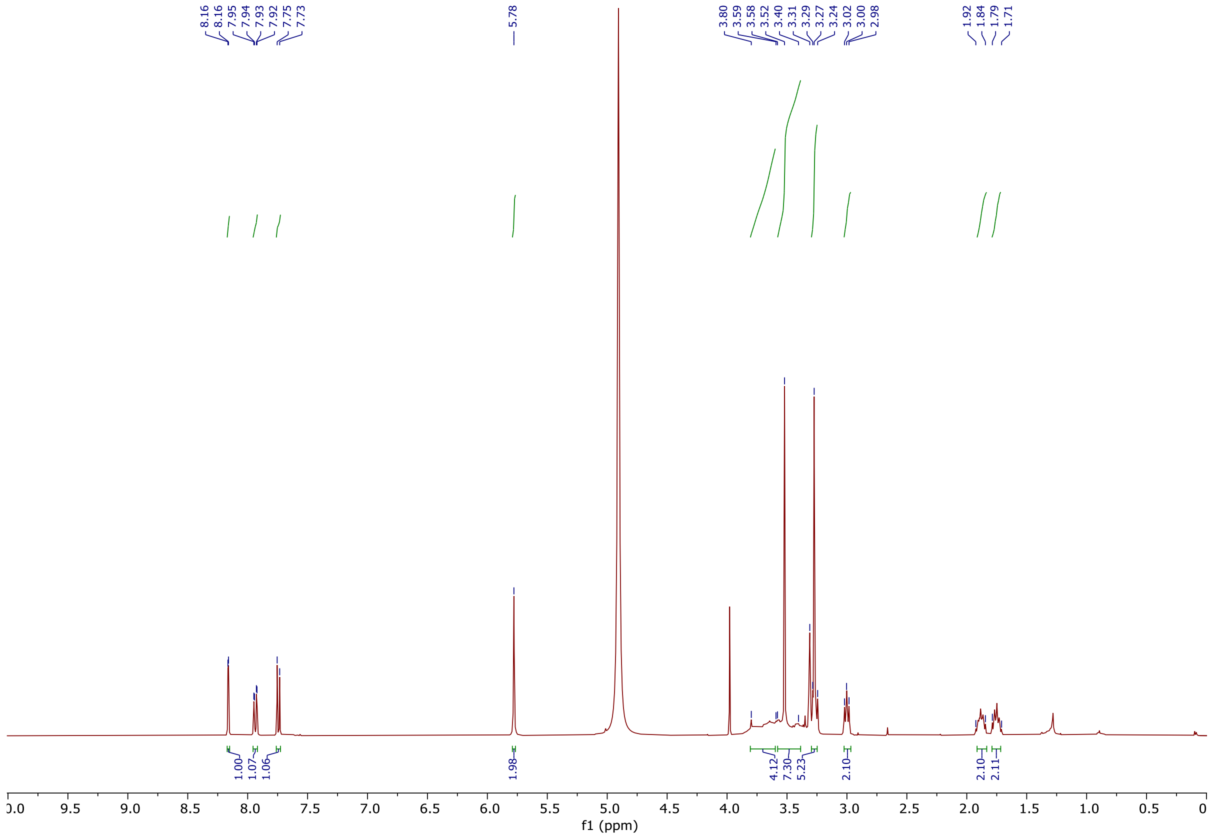

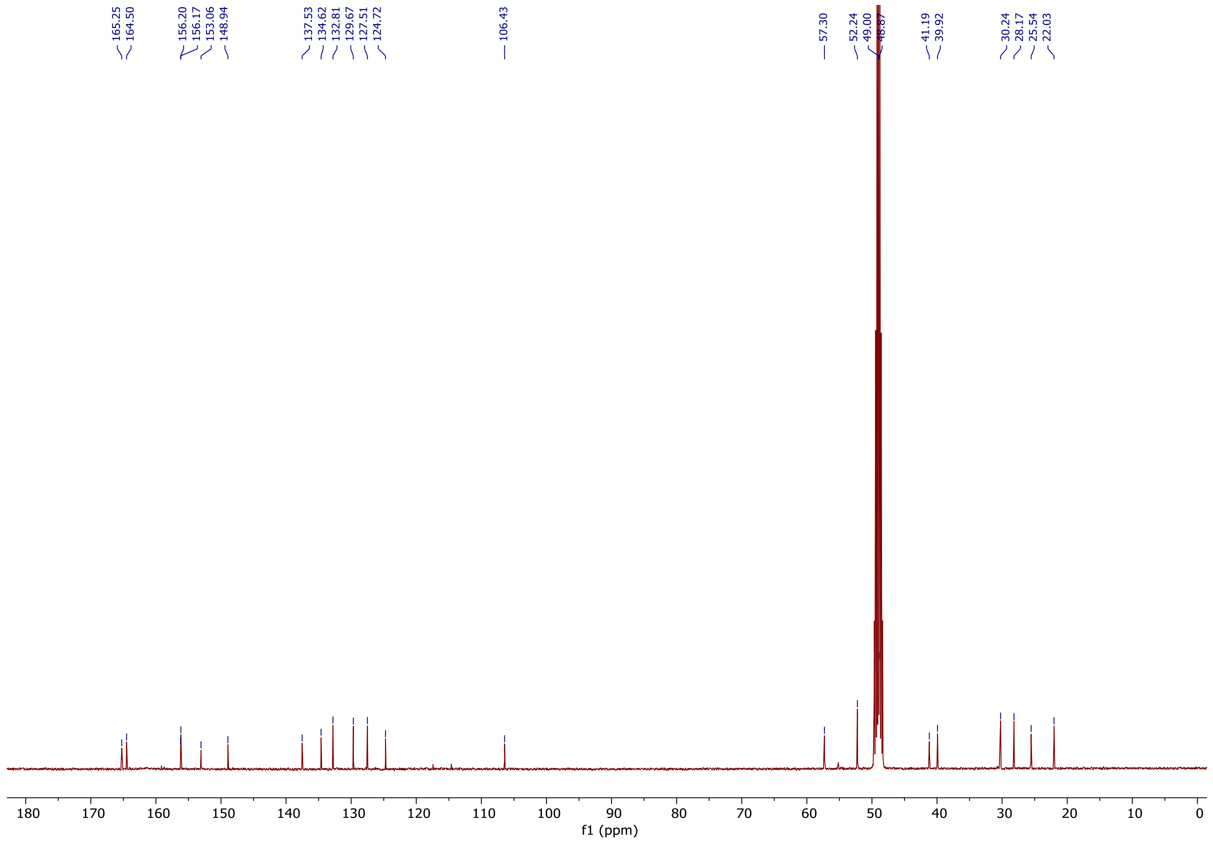

***N*-(4-(4-(7-((5-(3,4-dichlorophenyl)-1,3,4-oxadiazol-2-yl)methyl)-1,3-dimethyl-2,6-dioxo-2,3,6,7-tetrahydro-1*H*-purin-8-yl)piperazin-1-yl)butyl)-3-(5,5-difluoro-7-(1*H*-pyrrol-2-yl)-5*H*-5 λ^4,6 λ^4-dipyrrolo[1,2-*c*:2',1'-*f*][1,3,2]diazaborinin-3-yl)propenamide, CBH-004 (8)**

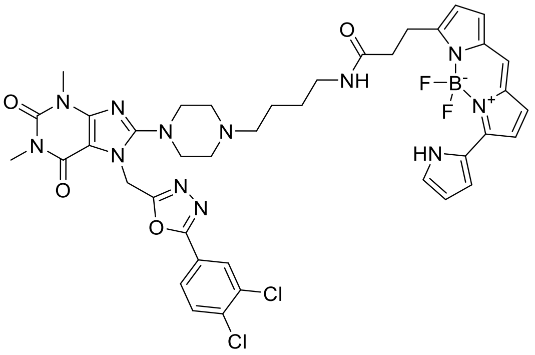

8

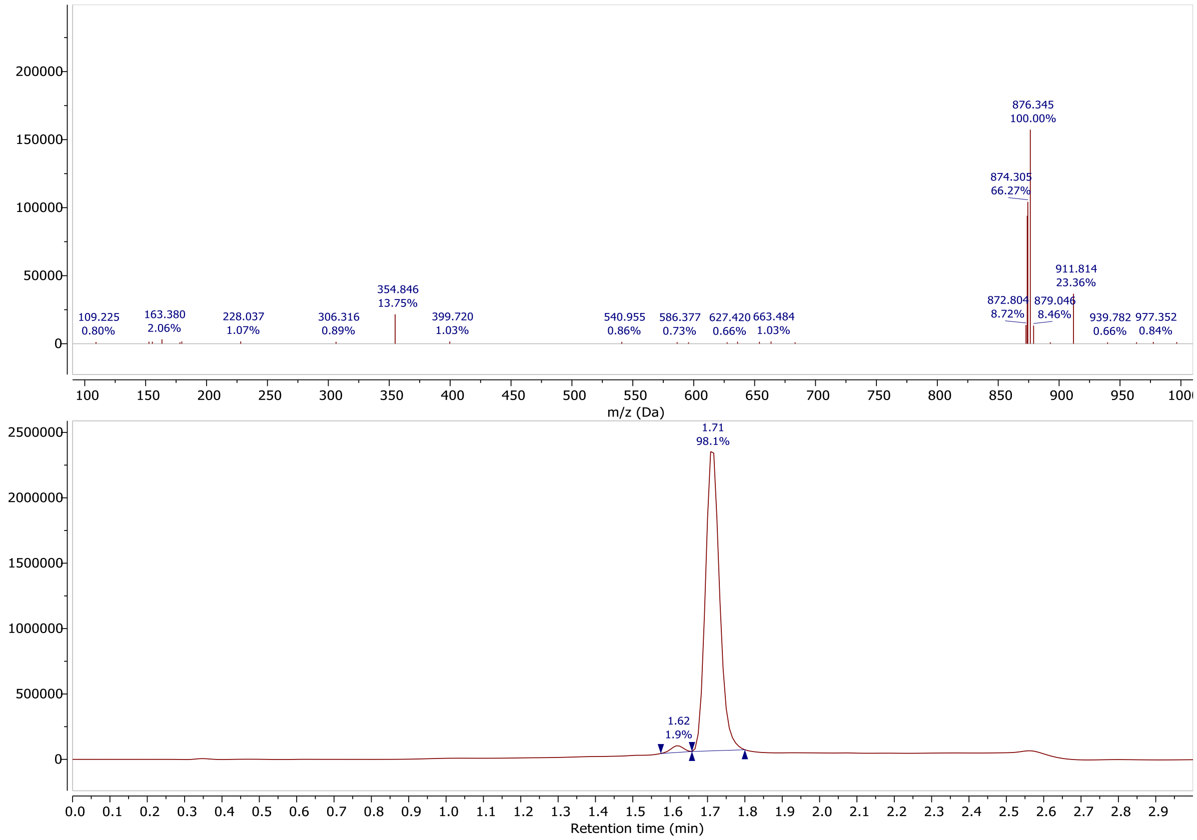

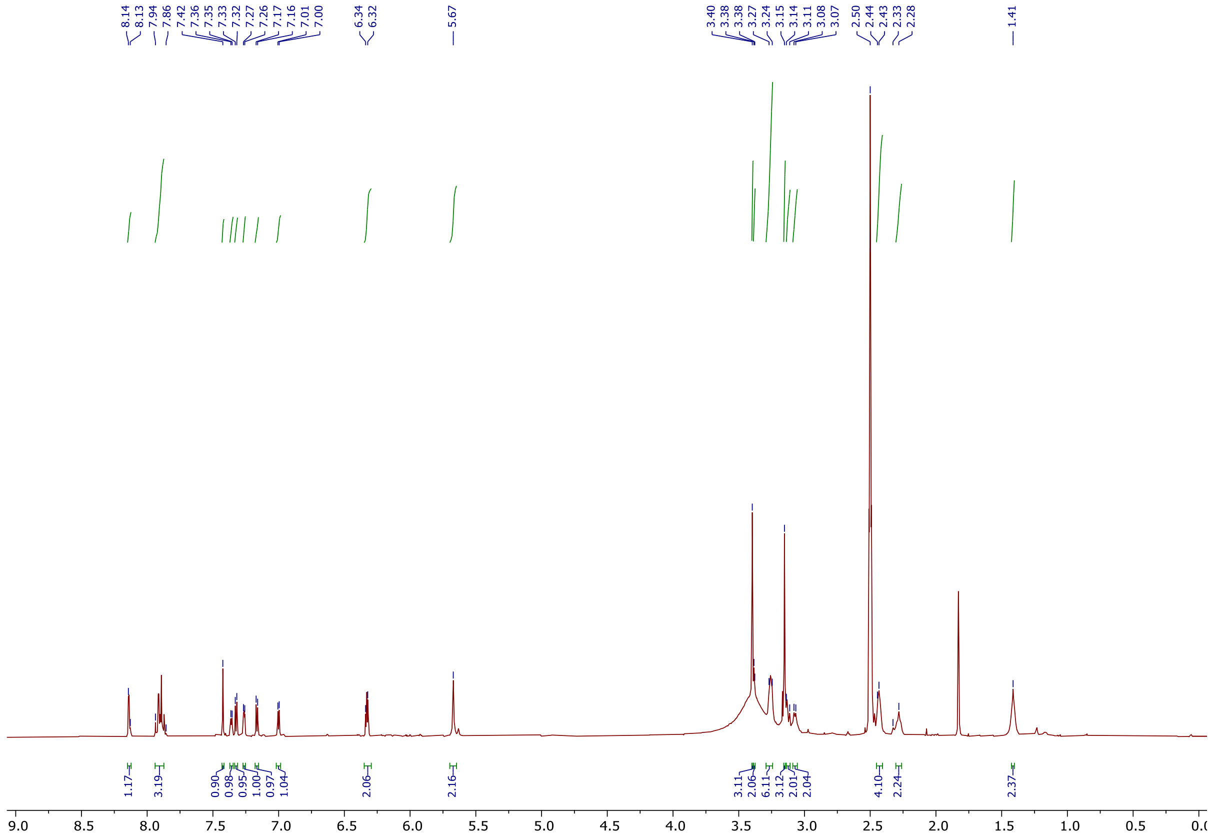
8.14
8.13
7.94
7.86
7.42
7.36
7.35
7.33
7.32
7.27
7.26
7.17
7.16
7.01
7.00
6.34
6.32
5.67
3.40
3.38
3.38
3.27
3.24
3.15
3.14
3.11
3.08
3.07
2.50
2.44
2.43
2.33
2.28
1.41
1.17
3.19
0.90
0.98
0.95
1.00
0.97
1.04
2.06
2.16
3.11
2.06
6.11
3.12
2.01
2.04
4.10
2.24
2.37
9.0
8.5
8.0
7.5
7.0
6.5
6.0
5.5
5.0
4.5
4.0
3.5
3.0
2.5
2.0
1.5
1.0
0.5

Table S2. X-ray data collection and refinement statistics.

Complex	NUDT5-ibrutinib (1)	NUDT5-compound 9	NUDT14-compound 9
**PDB accession codes**	**8RDZ**	**8RIY**	**8OTV**
**Data collection**			
Resolution range	49.51-2.02 (2.09-2.02)	83.77-2.29 (2.65-2.29)	54.05-1.82 (1.89-1.82) *P* 2_1 2_1 2_1
Space group	*P* 1	*P* 6_1 2 2	51.25, 90.97, 108.10
Unit cell a, b, c (Å)	47.71, 59.20, 79.43	60.61, 60.61, 502.61	90, 90, 90
Unit cell α, β, γ (°)	80.65, 82.96, 77.03	90, 90, 120	556659 (38748)
Total reflections	150478 (15280)	329168 (15042)	45867 (4293)
Unique reflections	53078 (5257)	9387 (469)	12.1 (9.0)
Multiplicity	2.8 (2.9)	35.1 (32.1)	98.06 (81.55)
Completeness (%)	96.87 (95.82)	85.8 (85.7) (*)	9.60 (0.33)
Mean I/ sigma (I)	7.35 (1.25)	10.3 (1.8)	0.127 (2.80)
R-merge	0.057 (0.875)	0.335 (2.54)	0.999 (0.382)
CC(1/2)	0.994 (0.243)	0.999 (0.835)	
**Refinement**			
R-work	0.226 (0.346)	0.229 (0.282)	0.206 (0.418)
R-free	0.285 (0.382)	0.295 (n/a)	0.239 (0.443)
R.M.S.D. bonds (Å)	0.462	0.005	0.005
R.M.S.D. angles (°)	5.84	1.37	0.76
**Molprobity Analysis**			
Ramachandran favoured (%)	94.63	94.72	96.85
Ramachandran allowed (%)	4.43	5.28	2.91
Ramachandran outliers (%)	0.94	0	0.24
Rotamers outliers (%)	3.56	3.91	0.29
Average B-factor	46.65	48.6	45.27
Macromolecules	46.27	48.8	45.18
Ligands	66.54	46.2	44.41
solvent	37.45	32.0	46.82

Statistics for the highest-resolution shell are shown in parentheses.

(*) Ellipsoid completeness from Staraniso

Table S3. Primers for cloning.

Primers	Sequence
NUDT5 forward	TACTTCCAATCCATGGAGAGCCAAGAACCAACGG
NUDT5 reverse	TATCCACCTTTACTGTCAATTTGCATGTTTCAGTGCTAGAGC
NUDT14 forward	TACTTCCAATCCATGGAGCGCATCGAGGGGGC
NUDT14 reverse	TATCCACCTTTACTGTCACTGGAGATCCAGGTTGGG
